# Mapping women’s work in India: An application of small area estimation

**DOI:** 10.1371/journal.pone.0317783

**Published:** 2025-02-19

**Authors:** Swati Srivastava, Kaushalendra Kumar, Lotus McDougal, Ashish Kumar Upadhyay, Katherine Hay, Abhishek Singh

**Affiliations:** 1 GENDER Project, International Institute for Population Sciences, Mumbai, Maharashtra, India; 2 Department of Public Health & Mortality Studies, International Institute for Population Sciences, Mumbai, Maharashtra, India; 3 Center of Demography of Gender, International Institute for Population Sciences, Mumbai, Maharashtra, India; 4 Center on Gender Equity and Health, University of California San Diego, La Jolla, CA, United States of America; Northeastern University, UNITED STATES OF AMERICA

## Abstract

**Background:**

Understanding variations in women’s work participation at lower administrative levels, such as districts, is a missing link in identifying trends, patterns and variation that can offer insights into this long-term stagnation. We link data from the 2019–21 Indian National Family Health Survey and the 2011 Indian Population and Housing Census to generate estimates of women’s work within 640 districts of India, and to examine the spatial clustering of women’s work across these districts. We examine women’s work through three outcome variables, namely, district-level estimates of 1) percentage of women who worked in the past 12 months, 2) percentage of women who were self-employed in the past 12 months, and 3) percentage of women who earned cash in the past 12 months.

**Results:**

Diagnostic measures confirm that our model-based estimates are robust enough to provide reliable district-level estimates of women’s work in India. Women’s work and cash earnings were lowest in the districts of the central, eastern, and northern regions, and highest in the southern region. Self-employment rates for women were generally low in Indian districts, except for districts in Himachal Pradesh and the north-eastern region.

**Conclusions:**

Considerable spatial heterogeneity in women’s work has been found across 640 districts of India. Our study demonstrates that estimated percentage of women who worked in the past 12 months, estimated percentage of women who earned cash in the past 12 months and estimated percentage of women who were self-employed in the past 12 months all vary substantially at the district level. Having only state-level estimates may thus be inadequate to inform efforts to remediate low levels of women’s work in India. The insights from our current study may help in the formulation and implementation of targeted policies that increase opportunities for women to expand their paid work in India.

## Introduction

Work, especially among women, is often considered as a measure of economic empowerment [1]. It can provide an opportunity to improve women’s wellbeing and capabilities, which may help empower them [2, 3]. However, women’s work participation has remained low in India in the last three decades, with fewer than a quarter of women working in 2018 [4]. A comparison with other BRICS countries (an intergovernmental organization comprising Brazil, Russia, India, China, South Africa, Iran, Egypt, Ethiopia and United Arab Emirates) suggests that India ranks last among the BRICS countries [5]. India ranked second last among the G-20 countries in terms of women’s work participation [5]. Among south Asian countries, India has the lowest female work participation [5]. Given these statistics, women’s work participation in India is a matter of concern.

Another important concern is that the static and decreasing trend in female work participation in India. Statistics from the Periodic Labour Force Survey (PLFS) shows that female labour force participation remained static between 25% and 27% between 1990 and 2005. Subsequently, there was a further decline in female labour force participation to 25% in 2017–2018 [4]. This trend stands in stark contrast to India’s dramatic rise in female literacy from 34% in 1990 to 69% in 2022 [6], rise in daily wages of women workers from $1.2 in 1990 to $2.8 in 2012 [7], decline in fertility rates from 3.4 children per woman in 2005 to 2.0 children per woman in 2021 [8], and high average annual GDP growth of 8.8% [9]. This was also the period when the largest employment generation programme, Mahatma Gandhi National Rural Employment Guarantee Act (MGNREGA), was successfully rolled-out in India.

These trends have shown some instability in recent years, with an increase in female labour force participation following the COVID-19 pandemic [10]. According to the 2022–2023 PLFS, the female labour force participation rate was 37%, which, though an increase of 12 percentage points from 2017–18 estimates, remains much lower than male labour force participation rate (79%). Female labour force participation varied by urban-rural residence; women in rural areas (42%) were more likely to work compared with women residing in urban areas (25%). Female labour force participation rates also varied across the Indian states; the lowest being in Bihar (11%) and highest in Himachal Pradesh (63%) [10].

Past studies have used female participation in the labour market, the labour force and the workforce, for analysing women’s participation in economic activities. While studies have interchangeably used these terms, these denote different dimensions of work [11]. While labour force includes all types of employment, labour market participation excludes unpaid family workers who do not enter the market [12]. Similarly, labour force consists of both employed and unemployed, whereas workforce captures only those who are employed. A number of studies in India used the term *laborforce or workforce* to examine women’s work [13]. Some studies used *paid employment* as a measure of women employment [14, 15]. A study by Jose (2008) analysed the relationship between women’s paid employment and their autonomy. He disaggregated the employed women into those who are paid and unpaid for their work and finally defined paid employment in the form of either cash or kind or both. Research by Lahoti and Swaminathan (2016) explored the relationship between economic development and women labour force participation; analyzed paid and unpaid work separately. They included any paid (cash or in kind) economic activity including self-employment, wage employment, and casual labour in paid work. On the other hand, unpaid work referred to assistance in the operation of a family farm or enterprise, and that did not receive regular remuneration in cash or kind. In sum, past studies have used women’s work in last 30 days, paid work, unpaid work or self-employment to examine women’s economic participation in India. Taking cue from the past studies, we used three indicators of women’s work—women who worked in the past 12 months, women who earned cash in the past 12 months and women who were self-employed in the past 12 months–in our study. We used these three indicators because women’s work participation in general and self-employment and cash earnings in particular can go a long way in improving the status of women [16].

Estimates from the 2019–21 National Family Health Survey (NFHS–5) indicate that in the past 12 months, only 31% of women worked (excluding household work) and only 4% of women were self-employed. One in four women (26%) earned cash in the past 12 months [8]. However, these estimates varied substantially across Indian states. Estimates of the percentage of women who worked in the past 12 months ranged between 11% in Lakshadweep and 49% in Manipur; women who earned cash in the past 12 months varied from 11% in Lakshadweep to 45% in Telangana and; women who worked as the self-employed varied from 2% in Karnataka to 17% in Sikkim. While a number of recent national and international research has examined the phenomena of women’s work in India, these papers lack detailed analyses of women’s work at lower administrative levels, such as districts, in India [13, 17–19]. States in India are often big comprising of a large number of districts. For example, Uttar Pradesh has 75 districts (Integrated Government Online Directory 2024) [20]. Studies have shown that state averages often mask significant district-level heterogeneity in health and development indicators [21–23].

While there is an array of data sources on women’s work in India, there are substantial limitations to all of them. The most common sources are 1) the decennial population and housing census, 2) nationwide quinquennial surveys on employment and unemployment by the National Sample Survey Organization (NSSO) under the Ministry of Statistics and Programme Implementation (MoSPI) of the Government of India, 3) PLFS, and 4) other large-scale household surveys, such as National Family Health Survey (NFHS) and India Human Development Survey (IHDS). In the Indian census, any member of the household (usually the head of the household) responds to the questions related to work of its members, offering data on women’s work at national, state, and district-levels. Unfortunately, the most recent Indian census was conducted in 2011, and is thus severely outdated. The NSSO data on employment and unemployment is available up to only 2011–12 [24]. The nationwide Employment and Unemployment (E&U) surveys have been replaced by the PLFS, conducted by the National Statistical Office (NSO) of MoSPI. The first PLFS was conducted in 2017–18 [25]. While the most recent round of PLFS was conducted in 2022–23, estimates of women’s work can only be derived at the national and state levels. In PLFS, as with the Indian census, any member of the household responds about the work of the other members. IHDS also provides estimates of women’s work in the country. However, the estimates can be derived only at the national level, and the most recent estimates are available for 2011–12. The NFHS, among others, has emerged as a key source of data providing estimates of women’s work only at the national and state levels, with sample size limitations restricting reliable district-level estimates of women’s work. The most recent round of NFHS was completed 2019–21. Women in the age-group 15–49 directly responded to the questions on work in 2019–21 NFHS (henceforth referred to as NFHS-5). A comparison of NFHS-5 estimates of women’s work in past 12 months (based on women age 15–49) with the PLFS estimates (based on women age 15–59) for the same reference period were similar at 31% (NFHS-5) and 32% (PLFS) [8, 26]. NFHS-5 thus offers a recent, and reliable means of estimating prevalences of women’s work in India.

While NFHS-5 has some advantages over other data sources for recent estimates of women’s work in India, small sample sizes at the district-level render the direct estimation of women’s work at district-level less reliable. Small-area estimation (SAE) techniques have emerged as a methodologically sound way to derive reliable estimates of demographic and health indicators at the district level [21, 22, 27]. We, therefore, used area-level SAE techniques to derive reliable estimates of women who worked in the past 12 months, women who worked as self-employed in the past 12 months, and women who earned cash in the past 12 months for the 640 districts of India. We further used spatial models to identify the potential clusters of districts with high and low levels of women’s work. District-level estimates of women’s work may help in appropriately resourcing and targeting districts with unusually low levels of women’s work.

## Materials and methods

### Data

Our study is based on an analysis of secondary data, the fifth round of the National Family Health Survey (NFHS-5) and the 2011 Indian Population and Housing Census. NFHS is a large-scale, multi-round survey conducted in a representative sample of households throughout India. The NFHS-5 fieldwork was conducted in two phases: phase one from June 17, 2019 to January 30, 2020, and phase two from January 2, 2020 to April 30, 2021. NFHS-5 interviewed a total of 724,115 women aged 15–49, with a 97% response rate [8]. Of these women, only 108,785 were randomly selected for the women’s work module, which included questions related to women’s work in past 12 months and 7 days. The average number of women interviewed per district in the women’s work module was 153, ranging between 14 in Khandwa district and 260 in Nagaur district.

The 2011 Indian Population and Housing Census was conducted between February 9 and 28, 2011, and included information on demography (population characteristics), economic activity, literacy and education, housing & household amenities, urbanization, fertility and mortality, scheduled castes and scheduled tribes, language, religion, migration, disability and many other socio-cultural and demographic data of India [28].

### Outcome variables

The three primary study outcome variables are district-level estimates of 1) percentage of women who worked in the past 12 months, 2) percentage of women who were self-employed in the past 12 months, and 3) percentage of women who earned cash in the past 12 months. The three outcome variables were derived from the following questions asked in NFHS-5:

Aside from your own housework, have you done any work in the last 12 months?

Women were coded as ‘worked in the past 12 months’ if they answered ‘yes to the above question.

NFHS-5 further asked

Do you do this work for a member of your family, for someone else, or are you self-employed?

Women were coded as ‘self-employed in the past 12 months’ if they answered yes for self-employment.

NFHS-5 also asked

Are you paid in cash or kind for this work, or are you not paid at all?

Women who received payment in cash only or in cash and kind were coded as earning cash.

Before asking these three questions, NFHS-5 provided a description of the work that women usually do apart from their own housework. For example, some women take up jobs for which they are paid in cash or kind and others sell things, have a small business or work on the family farm or in the family business.

We finally aggregated these indicators at districts-level to estimate the district-level prevalence of women who worked in the past 12 months, women who were self-employed in the past 12 months and women who earned cash in the past 12 months. The denominator for all the three outcome variables is all women age 15–49 interviewed for the women’s work module.

### Auxiliary information

In SAE analysis, two types of variables are required: outcome and auxiliary variables. Outcome variables measure the outcome of interest, and are usually derived from survey data. Auxiliary variables capture specific sociodemographic factors, and are required from the entire population. Typically, these auxiliary variables are derived from census or administrative records [29]. We included the following district-level information available in the 2011 Indian Population and Housing Census as auxiliary variables: percentage of households who are scheduled caste/ tribes (SC/ST), percentage of Muslim households, percentage of female literate and gender gap in literacy, percentage of female headed households, mean household size, mean age at marriage for females, spousal age gap, percentage of male child born in last one year, percentage of male migration in past 12 months, percentage of women exposed to media, percentage of rural population, socio-economic percentile, and the state of residence. The choice of auxiliary variables for our models is guided by the social, economic, and demographic determinants of women’s work identified in the existing literature [30–32]. We tested for multicollinearity before including these variables in the SAE models. We also tried different combinations of auxiliary variables and selected that combination of variables which explained maximum variation in the outcome variables. Details about the definitions of auxiliary variables has been given in S1 Text.

### Statistical analysis

SAE is a powerful technique for deriving estimates of variables of interest at administrative levels, such as districts in India, for which the sample size is not enough. SAE models combine data from a survey, from which the variables of interest are to be derived, with census or other administrative records that have information for all the residents of the administrative units for deriving small-area estimates. In SAE, the model-based estimates derive strength from the auxiliary variables [33]. Although SAE techniques are well established, its use has been limited in social and public health research [33]. Other details about SAE techniques are given in S2 Text. SAE techniques are classified into two broad types: unit-level and area-level random effects models. Unit-level models are used when auxiliary variables are available at the individual level, whereas area-level models are used when auxiliary variables are only available at aggregate (e.g., district) level [27, 29]. We adopted the area-level SAE approach, as auxiliary data were only available at the district-level.

We first derived district-level estimates of women who worked in the past 12 months, women who worked as self-employed in the past 12 months, and women who earned cash in the past 12 months, directly from the NFHS-5, accounting for survey weights; these estimates are henceforth called as direct survey-based estimates. These direct survey-based estimates were then linked to auxiliary variables using Generalized Linear Mixed Models (GLMM) with logit link functions to derive the model-based district-level estimates of the three outcomes for the 640 districts of India [29, 34]. The GLMM accounted for area-specific random effects, which provided strength to the model-based district-level estimates of the women who worked in the past 12 months, women who worked as self-employed in the past 12 months, and women who earned cash in the past 12 months [33].

### Diagnostic measures

We used two types of diagnostic measures (model diagnostic and diagnostic for the small-area estimates) to assess the validity of the fitted GLMM models and the reliability of the model-based district-level estimates of women who worked in the past 12 months, women who worked as self-employed in the past 12 months, and women who earned cash in the past 12 months. The model diagnostic was used to verify the assumptions of the underlying model. Under the GLMM framework, random area specific effects are assumed to follow a normal distribution with mean zero and a constant variance. If the model assumptions are upheld, then the area (district) level residuals are expected to be randomly distributed and not significantly different from the line y = 0 [34].

The diagnostics for small-area estimates are used to validate the reliability of model-based small area estimates obtained from GLMM models. These diagnostics include a) bias diagnostic, b) coefficient of variation (CV), and c) 95% confidence intervals (CIs) of model-based estimates [34]. The bias diagnostic is used to examine the deviation of the model-based district-level estimates from the direct survey estimates to validate the reliability of the model-based district-level estimates. The CVs are used to assess the improvement in the precision of the model-based estimates over the direct survey-based estimates. Estimates with low CV are considered more reliable. The 95% CIs of the model-based estimates and direct survey-based estimates are compared to validate the robustness of the model-based estimates.

### Spatial clustering of women’s work

We mapped the estimates of three indicators of women’s work to identify clustering of districts of high and low women’s work. We also mapped the estimates to examine whether the spatial distribution of one variable is associated with the spatial distribution of the other. By doing so, the estimates may be effectively used for policy analysis as the focussed interventions may be directed towards a group of neighbouring districts having low levels of women’s work. Mapping of estimates may help identify and target cluster of districts with low coverage of women’s work even in higher prevalence state contexts. Mapping of estimates may also help in identifying clusters of districts with high-level of women’s work in poor performing states. Identification of such clusters of districts may help in understanding what interventions may work for improving women’s work. We used univariate Local Indicator of Spatial Association (LISA) to obtain geographical clustering of women’s work across the 640 districts of India. We used bivariate LISAs to examine spatial association between district-level estimates of a) women who worked in the past 12 months and the women who worked as self-employed in the past 12 months and b) women who worked in the past 12 months and women who earned cash in the past 12 months.

LISA measures the correlation of neighbourhood values around a specific spatial location. It determines the extent of spatial non-stationery and clustering present in the data.

LISA shows high-high clustering (high prevalent districts surrounded by high prevalent neighbourhood), low-low clustering (low prevalent districts surrounded by low prevalent neighbourhood), and spatial outliers (low–high and high-low clusters). The high-high clusters are also called hot spots representing high prevalent district surrounded by high prevalent districts in terms of women’s work. The low-low clusters are termed as cold spots and characterized by low prevalent districts surrounded by low prevalent districts in terms of women’s work. The districts marked as not significant are those which are surrounded by districts with different patterns of women’s work. Bivariate LISA measures the local correlation between a variable and weighted average of another variable in the neighbourhood. Queen’s contiguity weight matrix was used for estimating LISA. District-level women’s work may be influenced by district-level characteristics, such as percentage poor, percentage of rural population, percentage of households who are SC/ST, etc. [35, 36]. These variables were all estimated using NFHS-5 data, and then used to conduct bivariate LISA between a) district-level estimates of poor households and the three estimates of women’s work, b) district-level rural estimates and the three estimates of women’s work, and c) district-level SC/ST estimates and the three estimates of women’s work. We coded the bottom two wealth quintiles (poorest and poorer) in NFHS-5 to estimate percentage of poor households in the district. District-level women’s work may be influenced by gender norms prevailing in the districts of India. To measure gender norms at the district-level, we estimated district-level India Patriarchy Index (IPI), proposed by Singh et al. 2021, for the 640 districts of India using NFHS-5 [37]. IPI included 5 dimensions—1) domination of men over women, 2) domination of older generation over the younger generation, 3) patrilocality, 4) son preference, and 5) socio-economic domination of men over women [37]. Higher index values indicate more rigid and restrictive gender norms. Please refer to Singh et al. (2021) for details about the IPI [37]. To examine the association between patriarchy and district-level women’s work, we estimated bivariate LISA between district-level estimates of IPI and the three estimates of women’s work.

We also estimated ordinary least square (OLS) regression and spatial error regression (SER) to examine the association between district-level characteristics and the three indicators of women’s work. Both the OLS and SER models examine the effects of various predictors on the outcome variables, however, spatial error models incorporate spatial dependence by the error term. The independent variables included in the regression models are percentage poor, percentage of rural population, percentage of households who are SC/ST, IPI, mean age of women, percentage of married women, percentage of women exposed to media, percentage of female headed households, percentage of female literate, percentage of Muslim households, mean household size, and mean number of children under the age of 5 years present in the household.

The LISA, OLS regression and SER were estimated using GeoDa version 1.12.1.161. SAE was carried out in STATA 16.

## Results

### Descriptive summary of women’s work

Direct-survey-based summary measures of women’s work at the national level are presented in Table 1. About 68% (n = 73,175) of sampled women lived in rural areas; 31% (n = 33,336) belonged to the scheduled caste or tribe and 81% (n = 87,179) were Hindu. A total of 18.4% (n = 19,904) belonged to the bottom 20% of wealth quintile; whereas 22.6% (n = 24,352) had no formal schooling. About 30.5% (95% CI: 29.9, 30.9) of women worked in the past 12 months, 3.5% (3.3, 3.7) women were self-employed in the past 12 months, and 25.4% (24.9, 25.8) of women earned cash in the past 12 months.

**Table 1:** Sample characteristics and prevalence of women’s work in India, 2019–21.

	N (Weighted)	% (Weighted)	Women who worked in the past 12 months	Women who were self-employed in the past 12 months	Women who earned cash in the past 12 months
			% (95% CI)		
**India**	1,08,785	100	30.5(29.9,30.9)	3.4(3.3, 3.7)	25.4(24.9, 25.8)
**Residence**					
Urban	34,839	32.3	26.8(25.8,27.9)	3.9(3.5,4.3)	25.0(24.0,26.0)
Rural	73,175	67.8	32.2(31.6,32.8)	3.3(3.1,3.5)	25.6(25.0,26.1)
**Caste**					
Schedule caste	23,427	21.7	33.5(32.5,34.5)	3.2(2.8,3.5)	29.5(28.5,30.5)
Schedule tribes	9,909	9.2	45.7(44.2,47.1)	4.3(3.8,4.8)	37.0(35.5,38.4)
Other backward classes	47,251	43.8	30.1(29.3,30.8)	3.2(3.0,3.5)	24.4(23.7,25.1)
Others	27,427	25.4	23.0(22.1,24.0)	3.8(3.5,4.2)	19.3(18.4,20.2)
**Religion**					
Hindu	87,179	80.7	32.6(32.0,33.2)	3.6(3.4,3.8)	27.0(26.4,27.5)
Muslim	15,138	14.0	16.3(15.1,17.6)	2.5(2.0,2.9)	14.1(13.0,15.3)
Christian	2,495	2.3	41.4(38.8,44.1)	6.5(5.2,7.7)	36.8(34.2,39.4)
Sikh	1,908	1.8	22.3(20.0,24.7)	2.8(2.0,3.7)	20.4(18.2,22.7)
Others	1,294	1.2	41.0(36.6,45.3)	4.2(2.7,5.8)	34.4(30.0,38.8)
**Wealth index**					
Poorest	19,904	18.4	35.1(34.0,36.2)	3.2(2.9,3.6)	27.7(26.7,28.7)
Poorer	22,049	20.4	33.6(32.6,34.5)	3.2(2.9,3.6)	27.6(26.7,28.6)
Middle	22,252	20.6	33.1(32.1,34.1)	3.3(2.9,3.7)	27.5(26.6,28.5)
Richer	22,376	20.7	27.7(26.7,28.7)	3.4(3.1,3.8)	23.7(22.8,24.7)
Richest	21,432	19.8	23.1(22.1,24.2)	4.2(3.8,4.7)	20.4(19.4,21.3)
**Education**					
No education	24,352	22.6	41.3(40.3,42.3)	3.0(2.7,3.3)	33.5(32.6,34.4)
Primary	12,664	11.7	37.3(36.0,38.5)	3.1(2.7,3.5)	31.1(29.8,32.3)
Secondary	54,095	50.1	25.0(24.4,25.7)	3.0(2.7,3.2)	20.6(20.0,21.2)
Higher	16,903	15.7	27.0(25.8,28.2)	6.1(5.6,6.6)	24.6(23.4,25.8)

### SAE model statistic

Auxiliary variables included in the models explained 74%, 76% and 56% of the variation in women worked in the past 12 months, women earned cash in the past 12 months and women were self-employed in the past 12 months respectively across the districts of India (S1 Table).

#### Diagnostics

District-level residuals of women who worked in the past 12 months, women who worked as self-employed in the past 12 months, and women who earned cash in the past 12 months are randomly distributed, confirming the normality assumption. This suggests that the model-based estimates of indicators of women work are robust, with means closer to their expected values (S1 Fig). To compare the level of consistency of model-based estimates and direct survey-based estimates we compare the proximity of the 45^0^ line (y = x) to the fitted regression line for both the estimates. The line of best fit was not significantly different from the line y = x at 5% level for the model-based estimates, indicating the consistency between the model-based and direct survey-based estimates (S2 Fig).

The CVs of the direct survey-based estimates are much larger than the model-based estimates (S3 Fig). Additionally, the fluctuations in the CVs of the direct survey-based estimates are considerably larger compared to those of the model-based estimates. This diagnostic indicates that the model-based estimates are more precise than the direct survey-based estimates. S4 Fig shows the 95% CIs of the direct survey-based estimates and the model-based estimates. The direct survey-based estimates have much wider 95% CIs compared to the model-based estimates, suggesting that the standard errors of the direct survey-based estimates are large and unreliable. The model-based estimates are more robust than the direct survey-based estimates. The diagnostics clearly show the power of the SAE techniques for producing unbiased, consistent, and reliable estimates of women who worked in the past 12 months, women who worked as self-employed in the past 12 months, and women who earned cash in the past 12 months in the 640 districts of India.

**Fig 1:**
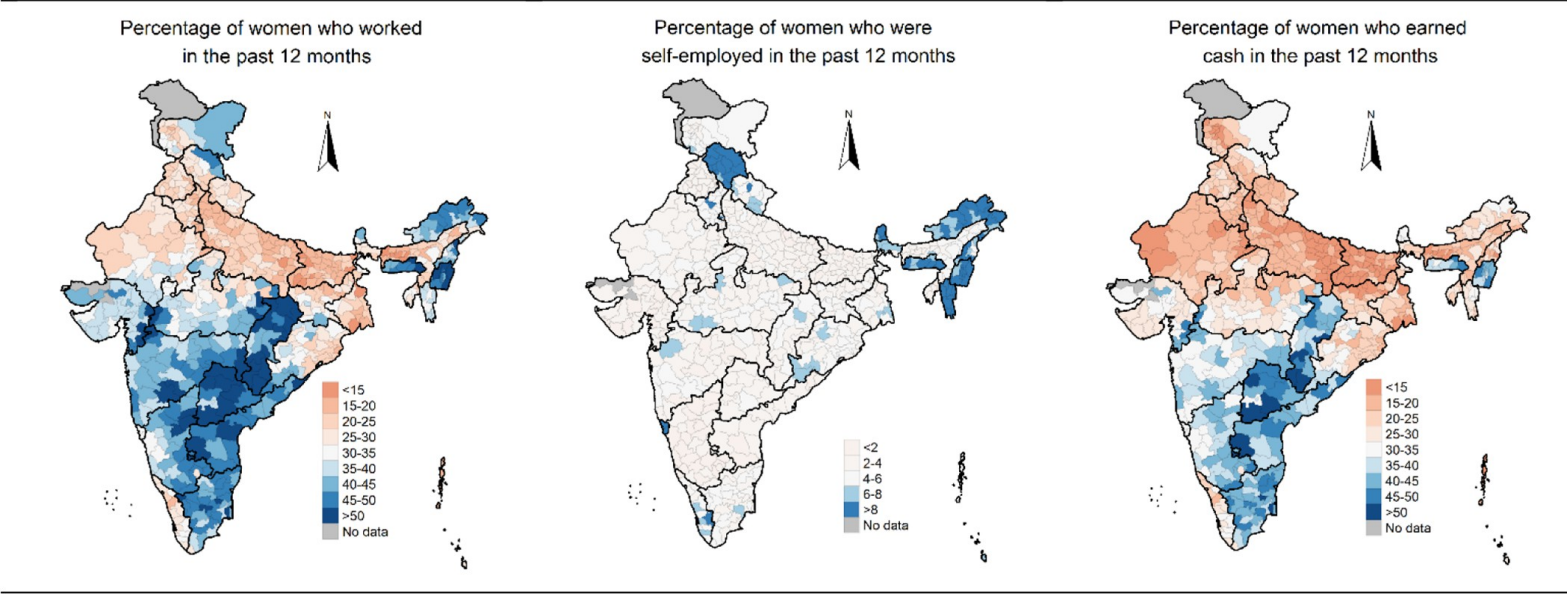
Percentage of women who worked in the past 12 months, women who were self-employed in the past 12 months and women who earned cash in the past 12 months in districts of India, 2019–21. Note: The base map can be found at https://globalsolaratlas.info/download/india.

The model-based estimates of all the three indicators of women’s work varied considerably across the districts of India (Fig 1). Estimates of women who worked in the past 12 months varied from a minimum of 10.9% (95% CI: 10.4%, 11.5%) in Arwal district of Bihar to a maximum of 61.4% (95% CI: 60.0%, 62.7%) in Bijapur district of Chhattisgarh. The estimated prevalence of women who worked as self-employed in the past 12 months ranged between 0.7% (95% CI: 0.6%, 0.8%) in Gopalganj district of Bihar to 19.8% (95% CI: 18.8%, 20.8%) in east district of Sikkim. The estimated prevalence of women who earned cash in the past 12 months varied from as low as 8.4% (95% CI: 8.2%, 8.7%) in Gopalganj district of Bihar to as high as 55.9% (95% CI: 55.5%, 56.3%) in Nizamabad of Telangana.

#### District level estimates of women’s work

We found considerable intra-state variations in the indicators of women’s work. For example, in Bihar, the state with the lowest coverage of women’s work in the past 12 months (17.5% (95% CI:16.5–18.6)), the district-level estimates varied from 10.9% (95% CI: 10.4–11.5) in Arwal to 27.0% (26.5–27.5) in Jamui. Bihar was followed by Uttar Pradesh in terms of prevalence; the prevalence of women who worked in the past 12 months in Uttar Pradesh was 20.7% (95% CI: 16.7–20.6). The lowest and highest prevalence of women’s work in the past 12 months in Uttar Pradesh were estimated for Azamgarh (14.8%, 95% CI: 14.5–15.0) and Aligarh (29.6%, 95% CI: 29.3–30.0) districts, respectively. The highest percentage of women who worked in the past 12 months was observed in Manipur (49.2%, 95% CI: 45.8–52.7), with prevalence ranging from 40.9% (95% CI: 40.0–41.9) in Imphal East to 61.0% (95% CI: 59.3–62.7) in Chandel. Manipur was followed by Telangana, with a prevalence of women’s work in the past 12 months of 48.2% (95% CI: 46.2–50.3), where the district-level prevalence ranged between 36.0% (95% CI: 35.7–36.3) in Hyderabad to 58.7% (58.3–59.1) in Nizamabad (Table 2).

**Table 2:** Direct-survey based estimates and model-based estimates of women who worked in the past 12 months, women who were self-employed in the past 12 months and women who earned cash in the past 12 months in districts of India, 2019–21.

State	Districts	Women who worked in the past 12 months		Women who were self-employed in the past 12 months		Women who earned cash in the past 12 months	
		Direct-survey based estimates	Model-based estimates	Direct-survey based estimates	Model-based estimates	Direct-survey based estimates	Model-based estimates
		Estimates (Lower bound, Upper bound)	Estimates (Lower bound, Upper bound)	Estimates (Lower bound, Upper bound)	Estimates (Lower bound, Upper bound)	Estimates (Lower bound, Upper bound)	Estimates (Lower bound, Upper bound)
Andaman & Nicobar Islands	Nicobars	11.2(6.1,16.2)	28.6(25.4,31.8)	2.3(0.7,7.2)	6.4(4.7,8.1)	9.5(5.6,13.5)	26.1(23.0,29.2)
Andaman & Nicobar Islands	North & Middle Andaman	17.5(11.0,24.1)	15.6(14.1,17.0)	6.9(3.7,12.6)	4.3(3.5,5.1)	15.5(8.6,22.4)	14.2(12.8,15.6)
Andaman & Nicobar Islands	South Andaman	30.7(20.1,41.3)	15.3(14.3,16.2)	6.9(3.7,12.4)	5.4(4.8,6.0)	30.7(20.1,41.3)	15.4(14.4,16.4)
Andhra Pradesh	Anantapur	57.0(46.3,67.7)	56.7(56.3,57.0)	0.7(0.1,4.5)	3.1(3.0,3.2)	52.5(41.6,63.4)	51.4(51.0,51.7)
Andhra Pradesh	Chittoor	51.6(40.3,62.9)	45.1(44.8,45.4)	0.0(0.0,0.0)	0.9(0.8,0.9)	40.8(30.2,51.4)	37.8(37.5,38.1)
Andhra Pradesh	East Godavari	40.0(36.3,43.6)	42.5(42.3,42.8)	4.2(1.7,9.8)	2.1(2.0,2.1)	36.0(30.2,41.8)	39.0(38.7,39.3)
Andhra Pradesh	Guntur	38.1(19.8,56.4)	48.2(47.9,48.5)	0.0(0.0,0.0)	2.5(2.4,2.6)	32.7(15.2,50.2)	40.6(40.3,40.8)
Andhra Pradesh	Krishna	53.8(47.4,60.1)	47.3(47.0,47.6)	1.2(0.3,4.8)	2.1(2.0,2.1)	51.3(41.6,61.1)	41.8(41.6,42.1)
Andhra Pradesh	Kurnool	56.9(49.1,64.7)	48.1(47.7,48.4)	1.4(0.3,5.5)	2.0(1.9,2.1)	45.5(39.6,51.3)	40.4(40.1,40.7)
Andhra Pradesh	Prakasam	50.7(41.7,59.8)	54.0(53.6,54.3)	3.5(1.3,9.0)	3.3(3.2,3.4)	43.3(35.4,51.2)	47.2(46.9,47.6)
Andhra Pradesh	Sri Potti Sriramulu Nellore	44.2(36.7,51.7)	47.2(46.8,47.5)	1.4(0.3,5.3)	2.1(2.0,2.2)	39.1(29.1,49.2)	41.9(41.5,42.2)
Andhra Pradesh	Srikakulam	44.0(34.5,53.5)	51.9(51.5,52.3)	2.9(0.9,8.6)	3.1(3.0,3.2)	41.5(33.0,50.0)	45.5(45.2,45.9)
Andhra Pradesh	Visakhapatnam	48.1(38.8,57.3)	47.2(46.9,47.5)	5.9(2.7,12.7)	2.6(2.5,2.7)	39.8(30.8,48.7)	46.9(46.6,47.3)
Andhra Pradesh	Vizianagaram	43.6(29.1,58.2)	43.0(42.6,43.4)	0.0(0.0,0.0)	1.8(1.7,1.9)	34.5(18.3,50.6)	36.3(35.9,36.7)
Andhra Pradesh	West Godavari	41.3(32.9,49.7)	43.9(43.6,44.2)	8.2(4.4,14.6)	2.9(2.8,3.0)	40.6(31.6,49.6)	38.9(38.6,39.2)
Andhra Pradesh	Y.S.R.	49.5(41.1,58.0)	43.8(43.4,44.1)	0.6(0.1,4.4)	1.6(1.5,1.7)	47.6(37.5,57.7)	37.4(37.1,37.8)
Arunachal Pradesh	Anjaw	59.2(48.8,69.6)	49.3(44.3,54.3)	12.0(7.3,19.2)	10.4(7.3,13.5)	24.4(15.6,33.1)	25.3(20.9,29.7)
Arunachal Pradesh	Changlang	24.7(11.5,38.0)	25.8(24.2,27.4)	4.6(2.3,9.0)	6.3(5.4,7.2)	16.1(4.1,28.2)	15.2(13.9,16.5)
Arunachal Pradesh	Dibang Valley	51.5(40.9,62.2)	49.7(41.7,57.7)	11.7(6.8,19.5)	9.9(5.1,14.6)	33.5(24.4,42.5)	33.0(25.5,40.5)
Arunachal Pradesh	East Kameng	39.6(23.8,55.4)	43.4(40.9,45.9)	9.5(5.1,17.1)	10.5(9.0,12.1)	26.5(16.1,36.9)	25.4(23.2,27.7)
Arunachal Pradesh	East Siang	39.6(25.5,53.6)	37.6(35.6,39.7)	8.0(4.7,13.3)	6.4(5.3,7.4)	26.1(15.5,36.8)	24.5(22.7,26.3)
Arunachal Pradesh	Kurung Kumey	34.6(16.8,52.3)	40.1(37.8,42.4)	7.8(4.5,13.2)	7.5(6.3,8.7)	24.3(12.2,36.4)	21.2(19.3,23.1)
Arunachal Pradesh	Lohit	29.8(19.5,40.2)	36.7(34.9,38.5)	5.4(3.4,8.5)	8.1(7.1,9.1)	16.8(11.6,22.0)	20.1(18.6,21.5)
Arunachal Pradesh	Lower Dibang Valley	57.4(43.3,71.6)	42.5(39.5,45.4)	11.4(7.0,18.1)	10.4(8.6,12.3)	25.6(6.8,44.5)	23.9(21.4,26.5)
Arunachal Pradesh	Lower Subansiri	45.0(34.0,56.0)	47.9(45.6,50.2)	12.6(8.1,18.9)	9.5(8.2,10.9)	26.3(17.4,35.3)	30.4(28.3,32.6)
Arunachal Pradesh	Papum Pare	36.3(25.3,47.2)	38.2(36.6,39.8)	5.2(2.3,11.3)	7.2(6.3,8.0)	28.8(20.6,36.9)	27.3(25.8,28.7)
Arunachal Pradesh	Tawang	36.4(29.2,43.6)	39.9(36.6,43.1)	7.1(4.1,12.0)	6.6(5.0,8.3)	24.2(17.3,31.0)	21.0(18.4,23.7)
Arunachal Pradesh	Tirap	35.9(28.1,43.7)	39.8(37.7,41.9)	6.2(3.9,9.7)	8.7(7.5,9.9)	21.3(15.2,27.3)	22.8(21.0,24.6)
Arunachal Pradesh	Upper Siang	52.3(39.9,64.7)	45.8(42.0,49.5)	15.8(9.7,24.6)	11.1(8.8,13.5)	29.9(24.1,35.6)	33.0(29.5,36.5)
Arunachal Pradesh	Upper Subansiri	53.5(40.3,66.7)	45.3(43.0,47.7)	11.1(6.7,17.8)	10.6(9.1,12.0)	32.0(24.4,39.6)	25.0(23.0,27.1)
Arunachal Pradesh	West Kameng	27.5(15.3,39.7)	38.4(36.0,40.8)	3.9(1.8,8.1)	6.9(5.7,8.2)	19.8(7.8,31.8)	24.7(22.6,26.9)
Arunachal Pradesh	West Siang	43.1(28.3,57.9)	46.2(44.2,48.3)	7.7(4.0,14.1)	9.8(8.5,11.0)	23.8(12.3,35.3)	26.4(24.6,28.2)
Assam	Baksa	8.9(4.3,13.4)	16.3(15.8,16.8)	2.3(0.9,6.0)	2.4(2.2,2.6)	8.9(4.3,13.4)	15.4(14.9,15.9)
Assam	Barpeta	8.5(3.2,13.8)	14.8(14.4,15.2)	3.5(1.6,7.6)	1.5(1.3,1.6)	7.9(2.9,12.9)	9.7(9.4,10.0)
Assam	Bongaigaon	10.9(5.2,16.6)	13.2(12.7,13.8)	5.2(2.6,10.0)	2.8(2.6,3.1)	10.6(4.8,16.4)	13.2(12.6,13.7)
Assam	Cachar	35.3(21.2,49.4)	22.0(21.6,22.4)	2.4(0.9,6.3)	4.3(4.1,4.5)	33.9(19.7,48.1)	19.2(18.8,19.6)
Assam	Chirang	7.9(2.5,13.3)	15.8(15.1,16.5)	0.8(0.2,3.5)	2.3(2.0,2.6)	7.3(1.5,13.1)	15.4(14.6,16.1)
Assam	Darrang	9.7(4.2,15.3)	21.5(20.9,22.1)	0.6(0.1,4.2)	3.8(3.6,4.1)	9.1(3.3,14.9)	18.1(17.5,18.6)
Assam	Dhemaji	18.4(8.5,28.3)	25.8(25.1,26.5)	1.3(0.3,4.9)	2.0(1.8,2.2)	9.8(4.9,14.7)	15.7(15.1,16.3)
Assam	Dhubri	8.1(4.8,11.4)	18.4(18.0,18.8)	0.9(0.3,2.7)	3.2(3.0,3.3)	7.4(4.0,10.7)	16.7(16.4,17.1)
Assam	Dibrugarh	33.6(19.4,47.8)	19.2(18.8,19.7)	5.2(2.6,10.1)	4.3(4.1,4.5)	32.2(17.8,46.7)	19.3(18.9,19.8)
Assam	Dima Hasao	24.9(17.6,32.1)	28.2(26.9,29.6)	5.5(2.8,10.6)	4.8(4.2,5.4)	23.0(13.5,32.5)	23.7(22.4,24.9)
Assam	Goalpara	16.8(9.8,23.8)	20.2(19.6,20.7)	5.2(2.7,9.6)	3.8(3.6,4.1)	14.5(8.7,20.4)	18.9(18.3,19.4)
Assam	Golaghat	35.2(18.5,51.8)	21.4(20.9,21.9)	9.0(5.4,14.7)	4.7(4.4,5.0)	33.9(17.6,50.2)	19.1(18.6,19.6)
Assam	Hailakandi	29.6(19.3,39.9)	23.6(22.9,24.3)	1.3(0.3,4.9)	2.3(2.0,2.5)	29.6(19.3,39.9)	24.8(24.1,25.6)
Assam	Jorhat	26.0(14.3,37.6)	26.5(26.0,27.0)	7.6(4.6,12.2)	5.6(5.3,5.9)	23.1(11.6,34.6)	22.2(21.7,22.7)
Assam	Kamrup	15.1(5.0,25.2)	14.9(14.5,15.3)	0.6(0.1,4.4)	3.1(2.9,3.3)	12.2(4.2,20.1)	14.4(14.0,14.8)
Assam	Kamrup Metropolitan	22.7(15.8,29.6)	22.5(22.0,23.0)	7.0(3.9,12.0)	6.2(5.9,6.5)	17.4(10.9,23.9)	23.9(23.5,24.4)
Assam	Karbi Anglong	25.0(15.0,35.1)	30.5(29.9,31.2)	3.9(2.1,7.0)	5.1(4.8,5.5)	18.4(11.7,25.1)	26.2(25.5,26.8)
Assam	Karimganj	33.3(20.2,46.4)	22.6(22.1,23.1)	3.0(1.2,6.9)	2.1(1.9,2.3)	32.7(19.7,45.7)	23.5(23.0,24.0)
Assam	Kokrajhar	19.1(8.8,29.3)	13.9(13.4,14.4)	3.2(1.3,7.7)	2.1(1.9,2.3)	18.5(8.0,29.0)	13.7(13.2,14.2)
Assam	Lakhimpur	28.2(17.0,39.4)	26.5(25.9,27.1)	5.1(2.5,10.4)	5.5(5.2,5.8)	24.5(12.4,36.7)	23.0(22.4,23.5)
Assam	Morigaon	14.8(6.8,22.9)	24.5(23.9,25.1)	2.3(0.9,5.9)	3.9(3.6,4.2)	14.2(6.2,22.3)	19.9(19.4,20.5)
Assam	Nagaon	12.7(5.3,20.1)	16.6(16.3,16.9)	3.6(1.7,7.6)	2.9(2.7,3.0)	12.2(5.1,19.2)	15.5(15.2,15.8)
Assam	Nalbari	13.7(4.1,23.3)	17.5(16.9,18.0)	2.9(1.1,7.5)	3.3(3.0,3.5)	11.9(3.9,19.9)	15.7(15.2,16.3)
Assam	Sivasagar	33.0(24.6,41.3)	24.1(23.6,24.7)	3.5(1.9,6.4)	4.0(3.8,4.3)	29.4(21.8,37.0)	22.8(22.3,23.3)
Assam	Sonitpur	20.5(13.7,27.2)	20.0(19.7,20.4)	4.4(2.4,7.9)	4.2(4.0,4.4)	18.2(12.1,24.3)	16.4(16.0,16.7)
Assam	Tinsukia	39.6(21.3,57.9)	28.1(27.6,28.6)	5.4(2.9,9.7)	4.5(4.2,4.7)	33.6(15.2,51.9)	24.9(24.4,25.4)
Assam	Udalguri	20.7(11.8,29.7)	23.4(22.7,24.0)	1.6(0.5,5.0)	2.5(2.3,2.8)	19.5(11.1,27.9)	22.7(22.1,23.3)
Bihar	Araria	19.1(10.7,27.4)	20.6(20.3,21.0)	0.5(0.1,3.7)	2.7(2.5,2.8)	17.9(10.3,25.6)	15.9(15.6,16.2)
Bihar	Arwal	14.8(6.5,23.0)	10.9(10.4,11.5)	1.6(0.5,5.0)	1.2(1.0,1.3)	11.0(4.9,17.1)	8.4(7.9,8.9)
Bihar	Aurangabad	13.9(8.5,19.3)	14.9(14.6,15.2)	1.3(0.3,5.3)	2.2(2.0,2.3)	12.7(7.7,17.8)	10.4(10.1,10.7)
Bihar	Banka	15.1(9.4,20.8)	16.2(15.8,16.6)	1.8(0.6,5.5)	2.6(2.4,2.7)	8.7(2.8,14.7)	11.3(10.9,11.6)
Bihar	Begusarai	13.0(6.5,19.5)	16.5(16.2,16.8)	1.2(0.4,3.6)	1.7(1.5,1.8)	9.5(4.4,14.6)	14.7(14.4,15.0)
Bihar	Bhagalpur	15.0(6.3,23.6)	17.7(17.4,18.1)	4.4(2.4,8.0)	3.6(3.5,3.8)	13.2(5.7,20.6)	12.9(12.7,13.2)
Bihar	Bhojpur	14.5(6.6,22.4)	15.4(15.1,15.7)	0.0(0.0,0.0)	2.1(2.0,2.2)	11.3(3.6,19.1)	11.0(10.8,11.3)
Bihar	Buxar	15.4(9.0,21.8)	15.2(14.8,15.6)	1.7(0.5,5.0)	2.0(1.9,2.2)	11.9(4.6,19.2)	11.0(10.7,11.4)
Bihar	Darbhanga	15.2(11.4,19.1)	17.5(17.2,17.8)	2.3(0.7,7.5)	1.4(1.3,1.5)	9.4(5.7,13.1)	9.3(9.0,9.5)
Bihar	Gaya	20.6(14.0,27.3)	19.5(19.2,19.7)	3.8(1.8,7.7)	2.8(2.7,2.9)	14.4(8.3,20.5)	14.2(14.0,14.4)
Bihar	Gopalganj	20.7(7.7,33.6)	14.0(13.7,14.3)	1.1(0.3,4.2)	0.7(0.6,0.8)	2.8(-0.8,6.3)	8.4(8.2,8.7)
Bihar	Jamui	25.7(15.6,35.8)	27.0(26.5,27.5)	3.5(1.6,7.6)	2.6(2.4,2.7)	25.0(15.1,34.9)	20.7(20.2,21.1)
Bihar	Jehanabad	22.8(16.8,28.8)	18.9(18.4,19.5)	3.2(1.4,7.0)	1.8(1.6,2.0)	14.5(8.7,20.3)	12.4(12.0,12.9)
Bihar	Kaimur (Bhabua)	20.3(13.9,26.8)	16.7(16.3,17.1)	3.3(1.4,7.8)	2.2(2.0,2.3)	13.0(9.6,16.5)	10.9(10.6,11.3)
Bihar	Katihar	15.1(7.0,23.2)	14.9(14.6,15.2)	1.5(0.4,5.9)	2.1(2.0,2.3)	13.1(6.2,20.0)	13.9(13.6,14.2)
Bihar	Khagaria	18.7(10.2,27.2)	16.3(15.8,16.7)	0.5(0.1,3.4)	2.2(2.0,2.3)	14.0(6.6,21.4)	12.7(12.3,13.1)
Bihar	Kishanganj	20.8(10.8,30.8)	14.0(13.6,14.4)	4.5(2.2,9.3)	2.0(1.8,2.2)	15.8(6.8,24.9)	11.1(10.7,11.5)
Bihar	Lakhisarai	22.8(15.6,30.1)	20.7(20.1,21.3)	4.5(2.1,9.7)	3.1(2.9,3.4)	18.2(11.4,25.0)	15.6(15.0,16.1)
Bihar	Madhepura	21.5(17.0,25.9)	17.5(17.1,17.9)	6.2(3.2,11.7)	3.7(3.5,3.9)	18.8(13.4,24.2)	14.8(14.4,15.2)
Bihar	Madhubani	10.4(6.2,14.5)	18.4(18.1,18.6)	1.4(0.3,5.4)	1.5(1.4,1.5)	6.9(3.7,10.0)	10.0(9.8,10.2)
Bihar	Munger	9.9(5.3,14.4)	19.9(19.4,20.4)	1.3(0.3,5.1)	2.8(2.6,3.0)	8.8(4.1,13.5)	14.9(14.5,15.3)
Bihar	Muzaffarnagar	9.9(5.2,14.5)	19.1(18.9,19.4)	0.6(0.1,4.1)	3.6(3.4,3.7)	9.3(4.8,13.8)	15.2(15.0,15.5)
Bihar	Nalanda	16.8(9.9,23.7)	17.8(17.5,18.1)	3.2(1.4,7.6)	2.7(2.5,2.8)	13.8(5.6,22.0)	11.9(11.6,12.2)
Bihar	Nawada	17.0(6.3,27.7)	17.0(16.6,17.3)	1.0(0.3,4.1)	1.0(0.9,1.1)	13.7(4.6,22.9)	8.8(8.5,9.0)
Bihar	Pashchim Champaran	17.3(10.3,24.3)	16.1(15.9,16.4)	4.6(2.2,9.3)	2.9(2.8,3.1)	7.5(4.3,10.7)	11.2(10.9,11.4)
Bihar	Patna	23.2(16.1,30.3)	20.5(20.3,20.7)	5.4(2.5,11.2)	3.8(3.7,3.9)	16.9(10.3,23.5)	16.5(16.3,16.8)
Bihar	Purba Champaran	22.9(11.3,34.4)	16.4(16.1,16.6)	1.2(0.3,4.6)	1.4(1.3,1.5)	12.6(8.0,17.2)	8.9(8.7,9.1)
Bihar	Purnia	33.1(21.4,44.9)	20.2(19.9,20.5)	3.1(1.3,7.3)	2.8(2.6,2.9)	27.2(15.8,38.6)	15.8(15.5,16.1)
Bihar	Rohtas	13.5(4.1,22.9)	12.3(12.0,12.6)	2.1(0.8,5.6)	1.8(1.7,1.9)	11.0(4.3,17.6)	9.5(9.3,9.8)
Bihar	Saharsa	14.1(5.3,22.9)	18.3(17.9,18.7)	1.4(0.4,5.5)	2.3(2.1,2.4)	8.7(1.1,16.2)	15.9(15.5,16.3)
Bihar	Samastipur	17.5(8.8,26.1)	16.9(16.7,17.2)	2.7(1.0,6.9)	3.0(2.9,3.1)	10.5(5.0,16.1)	11.4(11.2,11.6)
Bihar	Saran	17.1(9.1,25.1)	12.2(12.0,12.5)	4.7(2.3,9.1)	2.0(1.9,2.1)	13.8(7.7,19.9)	9.6(9.4,9.8)
Bihar	Sheikhpura	18.1(10.9,25.2)	21.2(20.4,21.9)	2.5(1.0,6.0)	3.2(2.9,3.5)	11.4(7.3,15.5)	13.7(13.0,14.3)
Bihar	Sheohar	8.2(-0.7,17.1)	18.1(17.4,18.8)	0.7(0.1,5.0)	2.5(2.2,2.8)	7.4(-1.3,16.2)	14.7(14.0,15.3)
Bihar	Sitamarhi	14.6(9.3,20.0)	17.0(16.7,17.3)	0.6(0.1,4.3)	2.6(2.5,2.8)	7.9(3.7,12.1)	13.2(12.9,13.5)
Bihar	Siwan	17.7(8.9,26.5)	15.5(15.2,15.8)	1.8(0.6,5.6)	2.9(2.8,3.0)	13.5(7.7,19.3)	11.3(11.1,11.6)
Bihar	Supaul	15.7(6.0,25.5)	23.3(22.9,23.7)	0.0(0.0,0.0)	2.8(2.7,3.0)	14.1(4.1,24.0)	17.0(16.6,17.4)
Bihar	Vaishali	19.5(12.2,26.9)	16.8(16.6,17.1)	4.1(2.0,8.4)	1.5(1.5,1.6)	13.0(7.7,18.2)	14.0(13.8,14.3)
Chandigarh	Chandigarh	22.0(15.2,28.9)	22.0(21.5,22.6)	0.7(0.1,4.5)	0.7(0.6,0.8)	22.0(15.2,28.9)	22.0(21.5,22.6)
Chhattisgarh	Bastar	56.9(47.7,66.2)	53.2(52.6,53.7)	3.4(1.8,6.2)	4.3(4.1,4.5)	43.9(33.7,54.1)	42.1(41.6,42.7)
Chhattisgarh	Bijapur	57.2(46.9,67.6)	61.4(60.0,62.7)	0.5(0.1,3.7)	3.0(2.5,3.4)	53.1(41.1,65.1)	50.9(49.5,52.2)
Chhattisgarh	Bilaspur	45.9(38.0,53.8)	51.1(50.6,51.5)	2.5(1.2,5.0)	4.1(4.0,4.3)	39.4(31.6,47.2)	46.1(45.7,46.5)
Chhattisgarh	Dakshin Bastar Dantewada	64.6(54.1,75.0)	61.0(60.1,61.9)	2.0(0.9,4.2)	3.0(2.7,3.3)	59.9(50.1,69.7)	54.8(53.9,55.7)
Chhattisgarh	Dhamtari	53.9(44.6,63.3)	55.3(54.6,56.1)	2.0(0.6,6.0)	3.7(3.4,3.9)	42.6(36.4,48.8)	46.5(45.7,47.2)
Chhattisgarh	Durg	43.9(38.1,49.7)	42.7(42.4,43.1)	4.0(2.7,6.0)	4.6(4.5,4.8)	36.3(31.2,41.5)	36.1(35.8,36.5)
Chhattisgarh	Janjgir—Champa	44.5(35.5,53.5)	54.0(53.5,54.5)	4.2(1.9,9.4)	5.2(5.0,5.5)	35.5(25.6,45.4)	43.4(42.9,43.9)
Chhattisgarh	Jashpur	55.0(44.6,65.5)	50.1(49.4,50.8)	7.0(3.7,13.0)	4.7(4.4,5.0)	45.0(34.7,55.3)	42.3(41.6,43.0)
Chhattisgarh	Kabeerdham	65.7(55.9,75.6)	50.0(49.3,50.8)	9.7(5.7,16.1)	4.0(3.7,4.3)	55.2(41.3,69.2)	41.1(40.3,41.8)
Chhattisgarh	Korba	40.9(29.4,52.3)	47.9(47.3,48.5)	4.9(2.4,9.7)	7.8(7.5,8.1)	31.1(23.8,38.4)	42.7(42.1,43.3)
Chhattisgarh	Koriya	57.2(44.6,69.9)	56.6(55.7,57.4)	8.9(5.0,15.4)	4.5(4.1,4.8)	44.2(32.3,56.2)	46.3(45.4,47.1)
Chhattisgarh	Mahasamund	42.9(32.7,53.0)	58.5(57.8,59.1)	4.9(2.6,8.9)	2.6(2.4,2.8)	35.0(26.6,43.5)	53.5(52.8,54.1)
Chhattisgarh	Narayanpur	59.0(46.8,71.2)	57.8(55.9,59.6)	1.4(0.3,5.6)	2.6(2.0,3.2)	52.0(38.3,65.7)	47.2(45.3,49.0)
Chhattisgarh	Raigarh	48.7(35.3,62.0)	53.7(53.2,54.2)	0.6(0.1,2.2)	3.6(3.4,3.8)	39.7(29.1,50.3)	41.8(41.3,42.4)
Chhattisgarh	Raipur	35.9(29.6,42.2)	42.3(42.0,42.7)	1.7(0.8,3.4)	2.2(2.1,2.3)	29.0(21.2,36.8)	37.6(37.2,37.9)
Chhattisgarh	Rajnandgaon	52.4(43.8,61.0)	37.0(36.5,37.5)	2.3(0.9,6.0)	2.8(2.6,2.9)	49.6(41.0,58.2)	31.1(30.6,31.6)
Chhattisgarh	Surguja	51.5(43.7,59.3)	50.1(49.7,50.6)	2.8(1.5,5.0)	4.3(4.1,4.5)	38.8(32.4,45.2)	38.3(37.8,38.7)
Chhattisgarh	Uttar Bastar Kanker	67.3(49.8,84.9)	60.8(60.0,61.5)	7.0(3.7,12.9)	2.9(2.7,3.2)	61.7(43.2,80.1)	50.4(49.6,51.2)
Dadra & Nagar Haveli	Dadra & Nagar Haveli	35.5(23.0,48.1)	35.6(34.4,36.7)	2.2(0.4,9.8)	1.5(1.2,1.8)	34.9(22.2,47.6)	33.2(32.1,34.4)
Daman & Diu	Daman	26.8(17.0,36.6)	22.6(21.1,24.1)	1.2(0.3,4.6)	0.8(0.5,1.1)	26.0(15.7,36.2)	21.3(19.8,22.7)
Daman & Diu	Diu	19.2(7.2,31.2)	23.4(21.0,25.8)	0.0(0.0,0.0)	1.0(0.5,1.6)	17.0(5.8,28.3)	23.4(21.0,25.8)
Goa	North Goa	38.3(28.6,47.9)	32.9(32.2,33.5)	11.1(6.2,19.0)	8.3(7.9,8.6)	37.6(28.1,47.1)	31.6(30.9,32.2)
Goa	South Goa	28.2(23.1,33.3)	33.6(32.8,34.3)	5.4(2.8,10.3)	8.2(7.8,8.7)	26.3(20.9,31.7)	32.3(31.6,33.0)
Gujarat	Ahmadabad	33.9(21.7,46.2)	37.5(37.3,37.7)	1.3(0.3,4.9)	2.5(2.4,2.6)	33.0(20.7,45.3)	31.1(30.9,31.4)
Gujarat	Amreli	44.9(33.7,56.0)	35.9(35.4,36.4)	0.0(0.0,0.0)	0.7(0.6,0.8)	35.5(25.0,46.0)	27.7(27.2,28.1)
Gujarat	Anand	44.9(30.7,59.1)	37.3(36.9,37.7)	1.2(0.3,4.9)	1.5(1.4,1.6)	32.5(16.0,48.9)	28.6(28.2,29.0)
Gujarat	Banas Kantha	31.7(16.4,47.0)	37.7(37.4,38.1)	0.0(0.0,0.0)	1.0(0.9,1.0)	23.5(9.6,37.4)	27.5(27.1,27.8)
Gujarat	Bharuch	29.1(16.8,41.5)	36.2(35.7,36.8)	0.9(0.1,6.1)	1.4(1.3,1.5)	26.0(14.0,38.0)	30.6(30.1,31.1)
Gujarat	Bhavnagar	48.6(31.6,65.6)	34.3(33.9,34.7)	1.5(0.4,5.9)	2.3(2.2,2.4)	31.8(20.0,43.7)	26.3(26.0,26.7)
Gujarat	Dohad	46.0(33.1,58.8)	53.1(52.6,53.6)	5.2(2.7,9.8)	2.6(2.4,2.7)	40.2(29.6,50.8)	32.8(32.3,33.2)
Gujarat	Gandhinagar	39.0(19.2,58.8)	40.7(40.1,41.2)	0.8(0.1,5.7)	1.8(1.7,2.0)	27.0(9.3,44.7)	30.7(30.2,31.2)
Gujarat	Jamnagar	28.0(17.1,39.0)	32.2(31.8,32.6)	0.0(0.0,0.0)	2.9(2.7,3.0)	16.3(7.5,25.1)	25.2(24.8,25.6)
Gujarat	Junagadh	36.1(28.7,43.5)	36.4(36.0,36.8)	2.6(1.3,5.2)	0.8(0.7,0.8)	25.4(18.9,31.9)	27.7(27.4,28.1)
Gujarat	Kachchh	32.9(23.5,42.2)	41.2(40.7,41.7)	2.9(1.2,6.7)	2.2(2.0,2.3)	23.1(14.1,32.0)	30.2(29.8,30.7)
Gujarat	kheda	49.2(40.2,58.2)	37.1(36.6,37.5)	1.2(0.4,3.5)	1.6(1.5,1.7)	37.3(27.9,46.6)	28.1(27.7,28.5)
Gujarat	Mahesana	34.7(25.7,43.8)	37.1(36.7,37.6)	6.1(2.9,12.2)	2.1(1.9,2.2)	27.8(19.0,36.5)	31.6(31.2,32.0)
Gujarat	Narmada	63.1(55.3,70.9)	58.9(58.1,59.8)	1.3(0.3,5.1)	2.3(2.0,2.6)	57.3(50.3,64.3)	47.3(46.5,48.2)
Gujarat	Navsari	60.1(51.1,69.2)	46.9(46.3,47.4)	0.7(0.1,4.5)	2.3(2.1,2.5)	58.2(47.4,69.0)	43.2(42.7,43.7)
Gujarat	Panch Mahals	40.7(33.5,48.0)	44.0(43.6,44.5)	5.1(2.7,9.5)	1.7(1.6,1.8)	20.6(12.0,29.3)	27.0(26.6,27.4)
Gujarat	Patan	47.2(37.7,56.7)	49.4(48.8,49.9)	2.3(0.7,7.0)	2.6(2.4,2.8)	38.0(28.1,47.9)	40.8(40.3,41.4)
Gujarat	Porbandar	24.0(20.1,27.9)	35.0(34.2,35.8)	1.7(0.5,5.0)	2.0(1.7,2.2)	20.6(16.3,24.9)	29.8(29.0,30.5)
Gujarat	Rajkot	35.8(25.4,46.1)	35.7(35.4,36.1)	0.0(0.0,0.0)	2.1(2.0,2.2)	25.7(15.9,35.5)	29.3(29.0,29.6)
Gujarat	Sabar Kantha	42.0(33.3,50.7)	37.8(37.4,38.2)	3.7(2.2,6.3)	1.6(1.5,1.7)	28.2(21.1,35.4)	27.3(26.9,27.6)
Gujarat	Surat	31.6(19.6,43.6)	37.8(37.6,38.1)	0.9(0.2,3.6)	2.9(2.8,3.0)	29.9(18.0,41.9)	31.0(30.8,31.3)
Gujarat	Surendranagar	35.0(26.5,43.4)	37.9(37.4,38.3)	3.2(1.7,5.9)	1.6(1.5,1.7)	27.6(20.0,35.2)	31.1(30.7,31.6)
Gujarat	Tapi	65.6(56.1,75.1)	60.0(59.3,60.6)	4.6(2.2,9.3)	1.6(1.4,1.8)	48.8(39.0,58.7)	46.5(45.8,47.2)
Gujarat	The Dangs	63.5(54.7,72.2)	59.0(57.6,60.4)	0.7(0.1,4.8)	2.5(2.0,2.9)	46.3(34.5,58.0)	43.5(42.1,45.0)
Gujarat	Vadodara	38.8(30.5,47.0)	37.2(36.9,37.5)	3.8(2.0,7.1)	3.4(3.3,3.5)	33.5(26.1,40.9)	29.4(29.1,29.7)
Gujarat	Valsad	38.7(19.4,58.0)	48.7(48.2,49.2)	0.6(0.1,4.2)	2.4(2.3,2.6)	34.6(18.5,50.6)	44.3(43.8,44.8)
Haryana	Ambala	24.1(15.4,32.8)	23.1(22.6,23.6)	2.9(1.3,6.0)	4.6(4.4,4.9)	22.2(13.5,30.8)	20.1(19.6,20.5)
Haryana	Bhiwani	16.0(11.6,20.5)	22.1(21.7,22.5)	3.6(1.9,6.8)	4.3(4.1,4.5)	12.4(9.0,15.9)	18.9(18.5,19.3)
Haryana	Faridabad	27.6(17.3,37.9)	21.6(21.2,22.0)	7.3(4.2,12.2)	5.4(5.1,5.6)	26.5(17.0,35.9)	19.7(19.3,20.1)
Haryana	Fatehabad	31.4(18.2,44.6)	25.9(25.3,26.5)	4.4(2.1,8.9)	5.2(4.9,5.5)	25.9(17.8,34.1)	23.0(22.5,23.6)
Haryana	Gurgaon	14.6(8.8,20.5)	19.3(18.9,19.8)	6.8(3.2,13.7)	5.2(5.0,5.5)	13.3(7.2,19.4)	16.2(15.8,16.6)
Haryana	Hisar	27.8(23.7,31.9)	25.7(25.3,26.2)	2.2(0.8,5.9)	4.4(4.2,4.6)	18.6(12.8,24.5)	21.0(20.6,21.4)
Haryana	Jhajjar	26.4(16.9,35.9)	26.0(25.4,26.6)	5.4(2.6,11.0)	4.2(4.0,4.5)	22.2(14.5,30.0)	19.7(19.2,20.2)
Haryana	Jind	17.7(11.0,24.5)	26.5(26.0,27.0)	4.7(2.4,9.0)	8.1(7.8,8.4)	16.5(9.3,23.6)	21.5(21.0,21.9)
Haryana	Kaithal	17.3(2.2,32.3)	23.6(23.0,24.1)	4.6(1.9,10.8)	4.5(4.2,4.7)	15.9(0.9,31.0)	22.4(21.9,23.0)
Haryana	Karnal	26.6(19.0,34.3)	18.4(18.0,18.8)	5.1(2.8,8.9)	4.3(4.1,4.5)	25.4(17.7,33.0)	16.2(15.8,16.6)
Haryana	Kurukshetra	18.9(14.6,23.1)	18.8(18.2,19.3)	4.6(2.1,10.0)	5.0(4.7,5.3)	16.2(11.2,21.2)	15.9(15.4,16.4)
Haryana	Mahendragarh	16.5(7.1,25.9)	22.0(21.4,22.6)	4.3(2.0,8.9)	4.5(4.2,4.8)	9.8(5.1,14.5)	16.2(15.7,16.7)
Haryana	Mewat	16.5(7.0,26.0)	18.8(18.2,19.3)	4.4(2.1,9.0)	2.6(2.4,2.8)	11.5(4.3,18.6)	13.5(13.0,14.0)
Haryana	Palwal	25.3(14.1,36.5)	18.0(17.4,18.5)	3.8(1.8,7.7)	2.4(2.2,2.6)	19.6(10.9,28.3)	14.2(13.8,14.7)
Haryana	Panchkula	16.0(8.2,23.7)	20.3(19.6,21.0)	1.8(0.4,7.0)	5.2(4.8,5.6)	16.0(8.2,23.7)	17.6(17.0,18.3)
Haryana	Panipat	28.8(19.7,38.0)	25.4(24.9,26.0)	3.1(1.1,8.2)	4.6(4.3,4.9)	24.7(16.2,33.3)	19.9(19.4,20.4)
Haryana	Rewari	30.9(21.7,40.1)	27.1(26.5,27.7)	5.7(2.9,11.0)	4.2(3.9,4.5)	15.0(9.3,20.6)	19.4(18.9,20.0)
Haryana	Rohtak	23.1(15.4,30.7)	22.3(21.8,22.8)	3.5(1.6,7.6)	5.6(5.3,5.9)	18.2(13.1,23.3)	18.2(17.8,18.7)
Haryana	Sirsa	34.4(-4.1,72.9)	25.3(24.8,25.8)	12.4(4.7,28.8)	4.2(4.0,4.4)	34.4(-4.1,72.9)	21.4(21.0,21.9)
Haryana	Sonipat	16.3(10.8,21.8)	22.6(22.1,23.1)	3.9(1.7,8.5)	5.9(5.6,6.1)	14.7(9.4,20.0)	19.1(18.6,19.5)
Haryana	Yamunanagar	21.3(15.4,27.3)	24.7(24.1,25.2)	4.4(2.1,8.8)	4.3(4.0,4.5)	16.6(10.9,22.2)	21.2(20.7,21.7)
Himanchal Pradesh	Bilaspur	25.6(17.4,33.8)	29.7(29.2,30.1)	8.4(4.8,14.3)	9.4(9.1,9.7)	18.6(14.0,23.1)	22.5(22.1,22.9)
Himanchal Pradesh	Chamba	35.0(25.8,44.1)	29.7(29.1,30.3)	15.6(9.2,25.1)	9.6(9.2,10.0)	28.4(22.7,34.1)	19.5(19.0,20.0)
Himanchal Pradesh	Hamirpur	43.6(24.2,63.1)	45.1(41.4,48.7)	14.7(8.9,23.3)	13.3(10.9,15.8)	34.3(18.0,50.5)	32.7(29.3,36.1)
Himanchal Pradesh	Kangra	30.6(19.2,42.0)	22.6(22.0,23.2)	12.0(6.9,19.9)	9.9(9.4,10.3)	23.8(14.9,32.8)	17.8(17.3,18.4)
Himanchal Pradesh	Kinnaur	30.4(19.8,41.1)	30.3(29.4,31.2)	9.4(5.3,16.2)	9.8(9.2,10.4)	15.1(7.6,22.5)	20.2(19.4,21.0)
Himanchal Pradesh	Kullu	44.2(33.6,54.8)	42.7(40.4,45.0)	10.1(6.0,16.7)	12.1(10.6,13.6)	27.9(20.3,35.4)	28.2(26.1,30.2)
Himanchal Pradesh	Lahul & Spiti	17.5(10.4,24.7)	28.8(28.0,29.7)	9.5(5.7,15.3)	8.4(7.9,8.9)	14.1(7.6,20.7)	21.3(20.5,22.0)
Himanchal Pradesh	Mandi	26.6(16.0,37.2)	23.4(22.6,24.2)	8.0(4.5,13.7)	8.3(7.8,8.8)	17.1(10.7,23.5)	17.6(16.9,18.3)
Himanchal Pradesh	Shimla	19.8(9.3,30.4)	21.8(21.1,22.6)	6.1(3.1,11.7)	8.8(8.3,9.3)	12.0(5.9,18.1)	16.8(16.2,17.5)
Himanchal Pradesh	Sirmaur	21.9(13.0,30.9)	26.3(25.6,27.1)	5.2(2.2,11.9)	7.7(7.3,8.2)	17.6(8.7,26.6)	20.8(20.1,21.5)
Himanchal Pradesh	Solan	28.8(19.4,38.2)	21.2(20.5,22.0)	8.8(4.6,15.9)	10.0(9.4,10.5)	24.9(15.6,34.3)	15.7(15.1,16.4)
Himanchal Pradesh	Una	24.7(15.3,34.1)	27.3(26.4,28.2)	11.3(7.0,17.8)	11.8(11.1,12.4)	19.3(15.2,23.3)	20.0(19.2,20.8)
Jammu & Kashmir	Anantnag	30.7(18.0,43.4)	23.8(23.2,24.4)	3.0(1.1,7.7)	3.3(3.1,3.6)	23.5(14.5,32.5)	13.7(13.2,14.2)
Jammu & Kashmir	Badgam	37.9(20.9,54.9)	22.5(21.8,23.2)	3.1(1.4,6.9)	2.5(2.3,2.8)	19.4(10.0,28.8)	14.3(13.7,14.8)
Jammu & Kashmir	Bandipore	25.7(15.4,36.0)	27.2(26.2,28.2)	3.6(1.6,7.9)	3.7(3.3,4.2)	19.6(12.2,27.1)	15.1(14.2,15.9)
Jammu & Kashmir	Baramula	39.9(26.4,53.4)	24.6(24.0,25.2)	5.4(2.9,9.8)	4.0(3.7,4.2)	24.7(15.9,33.4)	17.3(16.8,17.9)
Jammu & Kashmir	Doda	10.8(2.3,19.3)	24.0(23.1,25.0)	1.9(0.6,5.8)	4.3(3.8,4.7)	6.9(3.9,10.0)	13.7(12.9,14.4)
Jammu & Kashmir	Ganderbal	32.2(20.1,44.3)	24.4(23.3,25.5)	3.7(1.8,7.7)	3.8(3.3,4.3)	18.0(7.6,28.3)	13.5(12.6,14.4)
Jammu & Kashmir	Jammu	32.9(18.8,46.9)	32.8(32.3,33.3)	8.1(4.6,13.8)	7.4(7.1,7.7)	21.0(11.2,30.7)	21.3(20.9,21.8)
Jammu & Kashmir	Kargil	34.5(19.3,49.7)	39.0(37.2,40.9)	8.3(5.0,13.5)	5.1(4.2,5.9)	18.0(10.0,26.1)	22.3(20.8,23.9)
Jammu & Kashmir	Kathua	18.3(2.9,33.7)	29.2(28.4,30.0)	0.7(0.1,5.1)	4.1(3.8,4.5)	18.3(2.9,33.7)	21.5(20.8,22.2)
Jammu & Kashmir	Kishtwar	20.2(12.9,27.6)	29.8(28.5,31.1)	2.9(1.2,6.8)	5.5(4.9,6.2)	14.9(8.7,21.1)	18.1(17.0,19.2)
Jammu & Kashmir	Kulgam	16.3(5.7,26.8)	21.7(20.8,22.5)	8.1(4.8,13.2)	4.3(3.8,4.7)	10.0(3.5,16.5)	13.3(12.6,14.0)
Jammu & Kashmir	Kupwara	30.2(9.5,50.8)	26.7(25.9,27.4)	1.4(0.3,5.6)	5.4(5.0,5.7)	15.7(4.3,27.1)	14.5(13.9,15.1)
Jammu & Kashmir	Leh(Ladakh)	46.2(32.0,60.3)	43.1(41.2,45.0)	5.3(2.8,9.6)	5.8(4.9,6.7)	37.6(21.3,53.9)	33.4(31.6,35.2)
Jammu & Kashmir	Pulwama	11.4(7.5,15.4)	20.8(20.0,21.5)	2.3(0.9,5.9)	2.5(2.3,2.8)	9.1(6.0,12.2)	14.6(14.0,15.3)
Jammu & Kashmir	Punch	46.1(31.4,60.8)	33.8(32.9,34.8)	3.9(1.9,8.0)	4.1(3.7,4.5)	16.3(7.0,25.7)	19.1(18.3,19.9)
Jammu & Kashmir	Rajouri	39.2(23.5,55.0)	34.1(33.2,34.9)	7.9(4.7,12.8)	4.6(4.2,4.9)	26.0(12.5,39.4)	22.3(21.6,23.1)
Jammu & Kashmir	Ramban	34.7(21.6,47.7)	31.1(29.8,32.3)	4.3(2.2,8.4)	4.9(4.3,5.5)	18.5(12.5,24.5)	18.4(17.3,19.5)
Jammu & Kashmir	Reasi	21.0(6.5,35.5)	34.4(33.2,35.6)	9.8(5.8,16.2)	5.5(4.9,6.1)	8.0(4.3,11.8)	20.1(19.1,21.2)
Jammu & Kashmir	Samba	14.6(7.8,21.4)	28.3(27.2,29.3)	2.0(0.6,6.1)	4.6(4.2,5.1)	13.6(7.0,20.2)	20.3(19.3,21.2)
Jammu & Kashmir	Shupiyan	23.2(6.8,39.5)	25.2(24.1,26.4)	2.8(1.1,7.2)	3.7(3.2,4.2)	19.2(6.6,31.7)	17.3(16.3,18.3)
Jammu & Kashmir	Srinagar	23.4(12.7,34.1)	17.8(17.3,18.2)	4.6(2.2,9.4)	3.8(3.6,4.0)	14.4(9.0,19.8)	12.2(11.8,12.6)
Jammu & Kashmir	Udhampur	41.0(24.8,57.3)	36.1(35.2,37.0)	4.6(2.3,8.9)	4.8(4.4,5.2)	25.9(10.9,40.8)	22.4(21.6,23.1)
Jharkhand	Bokaro	22.6(12.5,32.6)	30.9(30.4,31.3)	2.6(1.1,6.2)	5.9(5.7,6.1)	18.0(8.6,27.4)	23.1(22.7,23.5)
Jharkhand	Chatra	25.7(13.2,38.2)	23.7(23.1,24.3)	1.7(0.6,5.2)	3.7(3.4,3.9)	19.8(10.4,29.3)	14.5(14.0,14.9)
Jharkhand	Deoghar	29.8(17.4,42.1)	26.4(25.9,26.9)	3.8(1.8,7.7)	3.3(3.1,3.6)	16.6(7.9,25.3)	15.5(15.1,16.0)
Jharkhand	Dhanbad	17.7(9.5,25.9)	23.7(23.4,24.1)	2.6(1.0,6.4)	4.1(4.0,4.3)	16.9(8.8,25.0)	18.8(18.4,19.1)
Jharkhand	Dumka	24.2(15.2,33.1)	28.6(28.0,29.1)	3.8(1.6,9.0)	5.7(5.4,6.0)	17.7(10.3,25.1)	20.9(20.4,21.4)
Jharkhand	Garhwa	14.0(8.4,19.6)	27.1(26.5,27.6)	3.4(1.5,7.4)	2.6(2.4,2.8)	10.7(6.3,15.1)	17.4(16.9,17.9)
Jharkhand	Giridih	18.3(11.5,25.1)	17.0(16.7,17.4)	0.0(0.0,0.0)	2.8(2.7,3.0)	12.8(6.8,18.8)	11.4(11.1,11.7)
Jharkhand	Godda	16.4(7.4,25.4)	24.5(24.0,25.1)	3.7(1.4,9.5)	3.2(2.9,3.4)	14.8(6.5,23.1)	19.0(18.5,19.4)
Jharkhand	Gumla	33.4(18.4,48.4)	28.2(27.5,28.8)	5.5(3.0,9.9)	5.0(4.7,5.3)	22.7(12.5,32.9)	20.6(20.0,21.1)
Jharkhand	Hazaribagh	36.2(21.2,51.3)	24.0(23.6,24.5)	1.6(0.5,4.9)	3.1(3.0,3.3)	15.7(9.1,22.4)	14.0(13.6,14.4)
Jharkhand	Jamtara	19.0(11.8,26.3)	17.4(16.8,18.0)	1.6(0.4,6.5)	2.2(1.9,2.4)	15.2(10.8,19.6)	12.7(12.2,13.2)
Jharkhand	Khunti	36.5(23.0,50.0)	31.7(30.8,32.6)	2.5(0.9,6.4)	2.6(2.3,2.9)	11.4(4.1,18.8)	21.3(20.5,22.0)
Jharkhand	Kodarma	11.5(5.6,17.3)	19.4(18.8,20.1)	1.4(0.5,4.4)	3.0(2.7,3.3)	8.8(4.1,13.6)	12.2(11.6,12.7)
Jharkhand	Latehar	33.0(18.6,47.5)	31.1(30.3,31.9)	3.3(1.5,7.3)	2.7(2.4,2.9)	27.1(15.9,38.3)	20.7(20.0,21.4)
Jharkhand	Lohardaga	46.6(35.3,57.9)	39.8(38.8,40.8)	5.1(2.6,10.0)	4.1(3.7,4.5)	40.8(28.1,53.6)	29.4(28.4,30.3)
Jharkhand	Pakur	23.1(14.5,31.6)	25.9(25.2,26.5)	1.9(0.7,5.0)	2.5(2.3,2.8)	21.1(12.6,29.6)	18.4(17.8,19.0)
Jharkhand	Palamu	26.3(17.9,34.7)	24.7(24.3,25.2)	6.7(3.8,11.6)	4.2(4.0,4.4)	12.9(6.9,19.0)	14.9(14.6,15.3)
Jharkhand	Pashchimi Singhbhum	52.5(35.6,69.4)	42.4(41.9,43.0)	8.8(4.9,15.3)	5.6(5.3,5.9)	31.1(13.4,48.9)	27.0(26.5,27.5)
Jharkhand	Purbi Singhbhum	22.0(10.3,33.8)	28.8(28.4,29.2)	2.5(1.0,6.0)	5.2(5.1,5.4)	18.6(8.2,29.1)	21.5(21.1,21.8)
Jharkhand	Ramgarh	28.5(21.3,35.8)	24.8(24.2,25.4)	5.0(2.3,10.7)	3.3(3.0,3.5)	22.1(14.9,29.2)	19.4(18.8,20.0)
Jharkhand	Ranchi	27.3(18.0,36.6)	26.8(26.5,27.2)	5.6(2.9,10.4)	5.1(4.9,5.3)	20.5(14.4,26.7)	21.6(21.3,21.9)
Jharkhand	Sahibganj	22.5(14.9,30.1)	22.4(21.8,22.9)	8.0(4.5,13.8)	5.0(4.7,5.3)	21.1(13.7,28.6)	17.6(17.1,18.1)
Jharkhand	Saraikela-Kharsawan	21.4(10.5,32.3)	31.2(30.6,31.8)	5.1(2.8,9.3)	4.3(4.1,4.6)	15.7(8.8,22.6)	21.0(20.4,21.5)
Jharkhand	Simdega	43.4(34.7,52.0)	31.3(30.4,32.1)	5.6(2.8,10.8)	2.7(2.4,3.0)	21.6(14.5,28.7)	21.2(20.5,22.0)
Karnataka	Bagalkot	36.8(30.2,43.3)	46.9(46.4,47.4)	1.8(0.6,5.5)	1.6(1.5,1.7)	35.2(27.8,42.6)	41.5(41.0,41.9)
Karnataka	Bangalore	28.7(15.7,41.7)	28.4(28.2,28.6)	2.4(0.8,7.1)	1.5(1.5,1.6)	27.9(15.0,40.7)	27.6(27.4,27.8)
Karnataka	Bangalore Rural	39.4(30.4,48.5)	45.9(45.2,46.5)	0.6(0.1,4.0)	1.7(1.5,1.8)	36.0(27.2,44.9)	42.4(41.8,43.0)
Karnataka	Belgaum	38.7(24.8,52.5)	44.5(44.2,44.8)	1.3(0.3,4.9)	2.1(2.1,2.2)	30.0(20.8,39.2)	38.0(37.7,38.2)
Karnataka	Bellary	48.2(35.7,60.7)	44.6(44.2,45.1)	0.6(0.1,4.2)	1.6(1.5,1.7)	46.3(33.5,59.1)	39.4(39.0,39.9)
Karnataka	Bidar	41.9(30.8,52.9)	44.2(43.7,44.7)	1.1(0.3,4.4)	2.1(2.0,2.3)	39.5(28.1,50.9)	36.9(36.4,37.4)
Karnataka	Bijapur	46.6(38.0,55.2)	46.5(46.0,46.9)	1.3(0.3,5.0)	1.6(1.5,1.7)	42.6(32.6,52.7)	41.4(40.9,41.8)
Karnataka	Chamarajanagar	44.3(32.2,56.5)	49.2(48.6,49.9)	1.1(0.3,4.3)	2.2(2.0,2.4)	41.8(29.6,54.0)	47.7(47.0,48.3)
Karnataka	Chikkaballapura	56.6(44.7,68.5)	46.5(45.9,47.1)	2.4(0.8,7.2)	1.5(1.4,1.6)	53.6(43.8,63.4)	44.2(43.7,44.8)
Karnataka	Chikmagalur	53.6(40.6,66.6)	42.6(42.0,43.1)	2.1(0.7,6.5)	1.5(1.3,1.6)	53.0(39.5,66.6)	41.6(41.1,42.2)
Karnataka	Chitradurga	52.6(37.8,67.5)	47.1(46.6,47.6)	3.1(1.2,7.9)	2.2(2.1,2.3)	41.2(30.5,52.0)	40.5(40.0,41.0)
Karnataka	Dakshina Kannada	39.4(26.5,52.3)	36.4(36.0,36.8)	0.5(0.1,3.7)	1.4(1.3,1.5)	36.0(23.8,48.3)	34.7(34.3,35.1)
Karnataka	Davanagere	38.5(29.7,47.3)	43.1(42.6,43.5)	4.2(1.7,9.6)	2.3(2.2,2.5)	29.9(20.3,39.5)	38.2(37.7,38.6)
Karnataka	Dharwad	36.0(29.8,42.3)	35.2(34.8,35.7)	3.7(1.6,8.1)	2.8(2.6,2.9)	31.8(26.5,37.0)	33.3(32.9,33.8)
Karnataka	Gadag	53.0(45.4,60.6)	42.1(41.5,42.8)	1.8(0.6,5.5)	1.8(1.7,2.0)	50.2(41.1,59.2)	39.3(38.7,39.9)
Karnataka	Gulbarga	43.0(38.3,47.6)	41.5(41.0,41.9)	5.4(2.7,10.5)	1.9(1.8,2.1)	32.1(22.6,41.6)	34.7(34.3,35.1)
Karnataka	Hassan	41.5(32.0,51.0)	38.6(38.2,39.1)	1.3(0.3,5.0)	1.6(1.5,1.7)	33.9(25.5,42.4)	38.0(37.5,38.4)
Karnataka	Haveri	37.0(28.3,45.8)	38.3(37.8,38.8)	1.7(0.5,5.0)	2.0(1.8,2.1)	31.4(22.8,40.1)	33.2(32.7,33.7)
Karnataka	Kodagu	36.6(25.5,47.7)	41.0(40.2,41.8)	1.4(0.4,5.5)	2.1(1.8,2.3)	36.6(25.5,47.7)	38.1(37.3,38.9)
Karnataka	Kolar	48.3(34.5,62.1)	49.5(49.0,50.0)	0.6(0.1,4.1)	3.5(3.3,3.7)	47.0(33.1,60.9)	45.8(45.2,46.3)
Karnataka	Koppal	47.5(38.4,56.6)	50.2(49.6,50.8)	0.7(0.1,4.8)	1.6(1.5,1.8)	43.1(33.1,53.1)	44.6(44.0,45.1)
Karnataka	Mandya	38.4(26.4,50.3)	42.6(42.2,43.1)	2.1(0.7,6.3)	2.4(2.2,2.5)	37.0(25.5,48.5)	38.1(37.7,38.6)
Karnataka	Mysore	41.1(33.8,48.3)	45.8(45.4,46.1)	0.0(0.0,0.0)	1.9(1.8,2.0)	39.8(33.0,46.6)	43.5(43.1,43.8)
Karnataka	Raichur	55.3(40.5,70.2)	50.8(50.3,51.3)	6.4(3.5,11.6)	2.0(1.9,2.2)	48.7(34.5,62.9)	39.9(39.4,40.3)
Karnataka	Ramanagara	37.6(27.4,47.9)	47.9(47.3,48.5)	0.8(0.1,5.7)	1.7(1.5,1.8)	35.3(24.7,45.8)	43.5(42.9,44.1)
Karnataka	Shimoga	39.3(33.0,45.5)	34.7(34.3,35.2)	0.7(0.1,4.7)	1.2(1.1,1.3)	39.3(33.0,45.5)	34.3(33.8,34.7)
Karnataka	Tumkur	42.3(29.6,54.9)	46.4(46.0,46.7)	3.2(1.2,8.2)	1.7(1.6,1.8)	35.9(24.0,47.7)	43.3(42.9,43.7)
Karnataka	Udupi	46.6(38.7,54.4)	39.3(38.7,39.8)	1.7(0.5,5.0)	1.8(1.6,1.9)	45.9(37.5,54.4)	39.1(38.6,39.6)
Karnataka	Uttara Kannada	32.4(21.2,43.7)	34.8(34.3,35.3)	1.5(0.5,4.8)	1.2(1.1,1.3)	25.7(19.4,32.0)	32.7(32.2,33.2)
Karnataka	Yadgir	56.6(44.2,69.0)	53.7(53.0,54.3)	0.7(0.1,5.0)	1.5(1.4,1.7)	52.4(38.7,66.1)	43.9(43.3,44.5)
Kerala	Alappuzha	30.8(24.1,37.5)	29.0(28.6,29.3)	5.8(2.4,13.3)	4.0(3.9,4.2)	30.8(24.1,37.5)	28.2(27.8,28.6)
Kerala	Ernakulam	35.0(25.3,44.8)	29.3(29.0,29.6)	4.9(2.0,11.2)	6.0(5.8,6.1)	35.0(25.3,44.8)	30.2(29.9,30.5)
Kerala	Idukki	45.1(33.1,57.0)	30.1(29.6,30.7)	9.8(5.5,16.9)	8.5(8.2,8.9)	42.3(28.9,55.7)	30.0(29.5,30.5)
Kerala	Kannur	19.2(12.8,25.5)	25.3(25.0,25.7)	6.7(3.4,12.8)	5.3(5.1,5.5)	19.2(12.8,25.5)	23.7(23.3,24.0)
Kerala	Kasaragod	23.0(17.7,28.4)	27.0(26.5,27.5)	0.6(0.1,4.0)	1.6(1.5,1.8)	23.0(17.7,28.4)	24.2(23.7,24.6)
Kerala	Kollam	34.4(20.0,48.9)	30.9(30.6,31.3)	7.4(3.7,14.0)	7.2(7.0,7.4)	34.4(20.0,48.9)	30.9(30.6,31.2)
Kerala	Kottayam	37.3(28.1,46.6)	27.4(27.1,27.8)	5.1(2.3,11.0)	6.2(6.0,6.4)	37.3(28.1,46.6)	27.1(26.7,27.4)
Kerala	Kozhikode	10.1(2.7,17.5)	24.4(24.1,24.7)	0.0(0.0,0.0)	4.4(4.3,4.5)	10.1(2.7,17.5)	24.8(24.5,25.1)
Kerala	Malappuram	13.8(5.9,21.8)	18.4(18.1,18.6)	1.2(0.3,4.7)	2.3(2.2,2.4)	13.8(5.9,21.8)	18.0(17.8,18.3)
Kerala	Palakkad	34.1(25.5,42.7)	28.4(28.1,28.7)	6.0(3.0,11.6)	4.3(4.2,4.5)	34.1(25.5,42.7)	28.0(27.6,28.3)
Kerala	Pathanamthitta	26.5(15.5,37.6)	33.7(33.2,34.3)	6.2(2.6,13.9)	4.7(4.5,4.9)	25.4(14.1,36.7)	33.9(33.4,34.4)
Kerala	Thiruvananthapuram	23.2(13.8,32.6)	25.0(24.7,25.3)	5.9(2.8,12.0)	5.2(5.1,5.3)	22.2(12.6,31.7)	25.6(25.3,25.9)
Kerala	Thrissur	31.6(23.1,40.1)	27.7(27.4,28.0)	9.5(5.2,16.9)	6.3(6.1,6.5)	30.7(20.9,40.4)	26.3(26.0,26.6)
Kerala	Wayanad	23.9(15.8,32.0)	31.5(30.9,32.2)	0.8(0.1,5.6)	3.8(3.5,4.0)	23.9(15.8,32.0)	31.5(30.8,32.1)
Lakshadweep	Lakshadweep	10.9(5.9,15.9)	10.9(9.3,12.5)	2.9(1.2,6.9)	2.9(2.1,3.8)	10.9(5.9,15.9)	10.9(9.3,12.5)
Madhya Pradesh	Alirajpur	73.0(62.2,83.8)	60.8(60.0,61.6)	3.9(1.7,8.5)	4.8(4.4,5.2)	45.5(36.1,55.0)	48.4(47.6,49.3)
Madhya Pradesh	Anuppur	51.2(38.8,63.6)	45.9(45.1,46.7)	5.8(3.0,10.7)	4.6(4.3,4.9)	30.7(20.3,41.1)	32.8(32.1,33.5)
Madhya Pradesh	Ashoknagar	17.4(8.1,26.7)	27.8(27.2,28.5)	0.9(0.1,6.3)	3.4(3.1,3.7)	15.4(6.7,24.2)	20.6(20.0,21.2)
Madhya Pradesh	Balaghat	56.8(48.9,64.7)	41.7(41.3,42.2)	0.0(0.0,0.0)	3.3(3.1,3.4)	53.9(47.5,60.2)	31.0(30.5,31.4)
Madhya Pradesh	Barwani	48.4(28.5,68.3)	44.8(44.2,45.4)	4.9(2.5,9.4)	5.5(5.3,5.8)	24.7(15.1,34.3)	30.2(29.7,30.8)
Madhya Pradesh	Betul	35.6(25.5,45.6)	40.4(39.9,41.0)	4.5(2.2,9.1)	3.8(3.6,4.0)	31.3(22.0,40.6)	29.5(29.0,30.0)
Madhya Pradesh	Bhind	14.6(10.4,18.9)	26.5(26.1,27.0)	3.1(1.2,8.0)	6.0(5.7,6.2)	8.5(6.0,10.9)	20.1(19.7,20.5)
Madhya Pradesh	Bhopal	22.0(15.5,28.4)	31.3(30.9,31.7)	6.1(1.5,22.2)	5.3(5.1,5.5)	22.0(15.5,28.4)	27.6(27.2,28.0)
Madhya Pradesh	Burhanpur	28.5(20.4,36.5)	34.5(33.8,35.3)	6.1(3.2,11.3)	3.3(3.0,3.6)	26.1(18.6,33.6)	22.8(22.1,23.4)
Madhya Pradesh	Chhatarpur	33.3(24.9,41.7)	25.6(25.1,26.1)	5.3(2.6,10.3)	3.3(3.1,3.5)	20.6(15.3,25.9)	19.3(18.8,19.7)
Madhya Pradesh	Chhindwara	48.1(35.3,60.8)	35.0(34.6,35.4)	8.9(5.2,14.7)	4.5(4.3,4.7)	32.0(22.5,41.4)	25.1(24.7,25.5)
Madhya Pradesh	Damoh	41.4(31.9,51.0)	44.6(44.0,45.2)	1.8(0.6,5.4)	2.8(2.6,3.1)	37.5(28.3,46.7)	37.5(36.9,38.1)
Madhya Pradesh	Datia	42.0(29.0,55.0)	37.3(36.6,38.1)	6.1(3.2,11.3)	2.5(2.3,2.7)	22.8(16.3,29.3)	27.0(26.3,27.7)
Madhya Pradesh	Dewas	34.5(25.8,43.1)	37.1(36.6,37.6)	2.8(1.1,6.7)	4.8(4.5,5.0)	30.2(20.0,40.3)	30.7(30.2,31.2)
Madhya Pradesh	Dhar	42.5(23.3,61.6)	45.7(45.2,46.1)	0.0(0.0,0.0)	3.0(2.9,3.2)	37.3(22.0,52.5)	34.0(33.5,34.4)
Madhya Pradesh	Dindori	53.7(40.4,66.9)	53.3(52.5,54.1)	7.8(4.7,12.6)	5.1(4.7,5.4)	40.5(30.2,50.8)	39.2(38.4,40.0)
Madhya Pradesh	East Nimar(Khandwa)	42.9(42.9,42.9)	35.2(34.6,35.7)	22.7(7.6,51.4)	7.9(7.6,8.2)	42.9(42.9,42.9)	27.9(27.4,28.5)
Madhya Pradesh	Guna	31.0(17.2,44.7)	31.1(30.5,31.7)	1.5(0.5,4.7)	7.1(6.7,7.4)	23.7(12.7,34.8)	24.4(23.8,24.9)
Madhya Pradesh	Gwalior	19.3(11.2,27.4)	28.5(28.1,29.0)	2.4(0.8,7.1)	4.1(3.9,4.3)	14.7(8.1,21.3)	24.5(24.1,24.9)
Madhya Pradesh	Harda	28.3(17.0,39.6)	38.8(37.9,39.7)	3.1(1.4,6.7)	4.8(4.4,5.2)	27.2(16.1,38.4)	32.9(32.1,33.8)
Madhya Pradesh	Hoshangabad	25.7(17.4,33.9)	38.6(38.1,39.2)	4.5(2.1,9.1)	4.3(4.1,4.6)	20.6(13.2,28.0)	31.0(30.4,31.5)
Madhya Pradesh	Indore	31.1(19.1,43.2)	30.9(30.6,31.3)	5.1(2.5,9.9)	4.3(4.2,4.5)	25.1(15.2,34.9)	25.6(25.3,25.9)
Madhya Pradesh	Jabalpur	49.4(31.4,67.4)	35.7(35.3,36.1)	0.0(0.0,0.0)	4.8(4.7,5.0)	37.7(18.2,57.2)	27.6(27.2,28.0)
Madhya Pradesh	Jhabua	63.0(51.2,74.8)	58.2(57.5,58.9)	1.5(0.4,5.8)	3.6(3.4,3.9)	43.3(38.2,48.4)	42.6(41.9,43.3)
Madhya Pradesh	Katni	24.1(12.7,35.4)	34.8(34.2,35.4)	1.3(0.2,8.7)	4.2(4.0,4.5)	17.1(6.9,27.2)	25.4(24.9,26.0)
Madhya Pradesh	Mandla	45.3(32.8,57.8)	42.7(42.1,43.3)	1.8(0.6,5.6)	2.9(2.7,3.2)	41.4(28.6,54.2)	34.2(33.6,34.8)
Madhya Pradesh	Mandsaur	47.6(31.4,63.8)	33.9(33.4,34.5)	4.7(2.3,9.6)	3.8(3.6,4.1)	36.2(22.9,49.5)	25.2(24.7,25.7)
Madhya Pradesh	Morena	24.9(16.0,33.8)	25.6(25.2,26.1)	6.0(3.0,11.6)	3.9(3.8,4.1)	10.4(5.5,15.4)	17.7(17.3,18.1)
Madhya Pradesh	Narsimhapur	35.8(24.9,46.6)	32.9(32.3,33.5)	3.8(1.8,7.8)	5.2(4.9,5.5)	27.3(18.8,35.8)	24.6(24.0,25.1)
Madhya Pradesh	Neemuch	44.4(33.5,55.4)	34.4(33.7,35.1)	3.1(1.3,7.1)	5.1(4.8,5.4)	34.1(24.1,44.0)	24.7(24.1,25.4)
Madhya Pradesh	Panna	27.8(19.0,36.6)	24.6(24.0,25.2)	1.8(0.6,5.5)	2.3(2.1,2.6)	20.9(14.1,27.6)	19.0(18.5,19.6)
Madhya Pradesh	Raisen	48.3(25.5,71.2)	35.8(35.2,36.4)	4.8(1.2,17.7)	3.9(3.7,4.2)	42.5(17.0,68.0)	29.7(29.1,30.2)
Madhya Pradesh	Rajgarh	33.5(23.2,43.8)	29.9(29.4,30.4)	0.0(0.0,0.0)	1.3(1.1,1.4)	21.7(15.9,27.5)	17.8(17.4,18.2)
Madhya Pradesh	Ratlam	21.8(16.6,27.0)	37.6(37.1,38.1)	3.3(1.4,7.8)	4.2(4.0,4.4)	18.5(13.5,23.5)	28.9(28.4,29.4)
Madhya Pradesh	Rewa	32.4(17.6,47.2)	40.4(40.0,40.8)	2.8(0.9,8.3)	6.2(6.0,6.4)	28.1(16.4,39.9)	29.9(29.4,30.3)
Madhya Pradesh	Sagar	27.1(20.3,33.8)	27.4(27.0,27.8)	6.4(3.5,11.5)	3.9(3.7,4.0)	18.1(13.1,23.0)	19.4(19.1,19.8)
Madhya Pradesh	Satna	21.1(8.5,33.7)	27.9(27.4,28.3)	5.3(2.0,13.2)	3.6(3.4,3.7)	15.9(6.9,24.9)	21.2(20.8,21.6)
Madhya Pradesh	Sehore	24.7(15.1,34.4)	34.3(33.7,34.9)	3.1(1.3,7.3)	3.7(3.5,3.9)	15.5(9.2,21.8)	25.4(24.9,25.9)
Madhya Pradesh	Seoni	41.1(30.8,51.4)	40.2(39.6,40.8)	1.9(0.6,5.8)	3.7(3.5,3.9)	36.1(24.4,47.8)	27.8(27.3,28.3)
Madhya Pradesh	Shahdol	55.8(43.3,68.3)	50.2(49.5,50.8)	3.7(1.5,8.9)	3.1(2.9,3.4)	49.1(34.7,63.6)	42.7(42.1,43.4)
Madhya Pradesh	Shajapur	31.6(23.2,40.0)	35.8(35.3,36.3)	4.4(2.4,7.8)	5.2(5.0,5.5)	21.1(14.3,28.0)	25.7(25.2,26.1)
Madhya Pradesh	Sheopur	53.0(45.1,60.8)	44.7(43.8,45.5)	9.0(5.2,14.9)	5.9(5.5,6.3)	33.0(22.3,43.6)	31.9(31.1,32.7)
Madhya Pradesh	Shivpuri	38.2(25.0,51.4)	35.7(35.2,36.3)	4.6(1.5,13.3)	5.4(5.2,5.7)	22.3(13.9,30.8)	25.4(24.9,25.8)
Madhya Pradesh	Sidhi	25.6(15.6,35.5)	24.1(23.6,24.7)	3.1(1.3,7.4)	2.2(2.0,2.4)	21.3(10.4,32.2)	18.4(17.9,19.0)
Madhya Pradesh	Singrauli	43.4(23.5,63.3)	46.9(46.3,47.6)	1.1(0.2,7.2)	2.6(2.4,2.8)	35.3(17.9,52.7)	37.2(36.6,37.9)
Madhya Pradesh	Tikamgarh	42.9(31.2,54.6)	36.8(36.2,37.3)	8.1(4.2,15.1)	5.3(5.1,5.6)	16.7(8.2,25.2)	24.4(23.9,24.9)
Madhya Pradesh	Ujjain	33.3(22.0,44.6)	36.2(35.7,36.6)	3.4(1.4,8.0)	3.5(3.3,3.7)	30.2(19.5,41.0)	29.1(28.6,29.5)
Madhya Pradesh	Umaria	32.0(15.9,48.2)	38.7(37.9,39.6)	8.2(4.3,15.2)	4.6(4.2,4.9)	19.5(10.9,28.0)	27.1(26.4,27.9)
Madhya Pradesh	Vidisha	23.3(14.9,31.7)	30.8(30.2,31.3)	3.5(1.5,8.2)	3.6(3.3,3.8)	21.9(14.2,29.5)	22.1(21.6,22.6)
Madhya Pradesh	West Nimar (Khargone)	34.4(23.6,45.2)	34.9(34.5,35.4)	6.2(3.3,11.1)	7.7(7.5,8.0)	26.4(18.4,34.3)	27.4(27.0,27.9)
Maharashtra	Ahmadnagar	35.1(25.7,44.6)	43.8(43.5,44.1)	4.4(1.9,9.8)	3.0(2.9,3.1)	27.2(17.1,37.3)	33.3(33.0,33.6)
Maharashtra	Akola	32.1(25.7,38.5)	40.0(39.5,40.5)	1.5(0.5,4.6)	3.1(2.9,3.3)	28.6(21.5,35.6)	36.2(35.7,36.7)
Maharashtra	Amravati	40.6(23.4,57.8)	43.2(42.9,43.6)	0.7(0.1,4.6)	3.6(3.5,3.8)	40.6(23.4,57.8)	39.3(38.9,39.7)
Maharashtra	Aurangabad	40.6(31.0,50.3)	41.7(41.3,42.0)	2.9(1.0,8.3)	4.0(3.8,4.1)	31.9(24.5,39.3)	37.2(36.8,37.5)
Maharashtra	Bhandara	57.2(42.5,71.9)	41.7(41.2,42.3)	1.5(0.4,5.7)	1.6(1.4,1.7)	52.2(36.7,67.7)	35.9(35.4,36.5)
Maharashtra	Bid	41.1(31.5,50.7)	37.5(37.1,37.9)	0.0(0.0,0.0)	1.4(1.3,1.5)	30.4(18.9,41.9)	30.8(30.4,31.2)
Maharashtra	Buldana	37.9(28.8,47.0)	38.2(37.8,38.6)	0.0(0.0,0.0)	1.4(1.3,1.5)	33.6(24.7,42.6)	31.9(31.5,32.3)
Maharashtra	Chandrapur	24.6(18.8,30.5)	47.8(47.4,48.2)	0.6(0.1,4.0)	3.9(3.7,4.1)	23.1(17.3,28.9)	41.7(41.3,42.1)
Maharashtra	Dhule	54.0(45.5,62.5)	47.0(46.6,47.5)	6.6(3.4,12.2)	3.3(3.2,3.5)	41.7(35.0,48.5)	40.1(39.7,40.6)
Maharashtra	Gadchiroli	55.6(44.1,67.1)	54.3(53.7,54.9)	2.0(0.7,6.2)	3.2(3.0,3.4)	46.1(37.0,55.2)	41.7(41.1,42.3)
Maharashtra	Gondiya	46.2(35.4,57.0)	36.0(35.5,36.6)	0.0(0.0,0.0)	3.1(2.9,3.3)	40.8(32.2,49.4)	28.1(27.6,28.6)
Maharashtra	Hingoli	42.9(29.9,55.9)	43.1(42.4,43.7)	1.7(0.6,5.2)	3.5(3.3,3.7)	35.8(23.3,48.3)	33.5(32.9,34.1)
Maharashtra	Jalgaon	47.5(36.2,58.8)	36.3(36.0,36.6)	3.0(1.1,7.7)	2.3(2.2,2.4)	46.9(35.6,58.2)	31.4(31.1,31.6)
Maharashtra	Jalna	59.4(46.2,72.6)	47.6(47.1,48.1)	2.3(0.7,6.9)	2.8(2.7,3.0)	45.5(32.4,58.6)	40.0(39.5,40.4)
Maharashtra	Kolhapur	40.6(30.2,51.0)	38.2(37.8,38.5)	2.4(0.9,6.3)	2.9(2.8,3.0)	36.2(24.8,47.6)	34.3(34.0,34.6)
Maharashtra	Latur	37.9(26.5,49.3)	43.6(43.2,44.0)	0.7(0.1,5.0)	2.6(2.5,2.7)	35.6(25.1,46.1)	35.7(35.3,36.2)
Maharashtra	Mumbai	37.4(25.1,49.6)	37.3(36.9,37.7)	3.0(1.2,7.2)	3.5(3.3,3.6)	37.4(25.1,49.6)	35.7(35.3,36.0)
Maharashtra	Mumbai Suburban	40.4(28.8,51.9)	37.2(37.0,37.4)	6.1(2.3,15.3)	4.3(4.2,4.4)	40.4(28.8,51.9)	34.7(34.5,34.9)
Maharashtra	Nagpur	21.1(10.7,31.5)	37.0(36.8,37.3)	1.3(0.3,5.0)	4.2(4.1,4.4)	18.3(10.2,26.5)	33.1(32.9,33.4)
Maharashtra	Nanded	38.5(28.8,48.2)	48.6(48.2,48.9)	0.0(0.0,0.0)	2.9(2.8,3.0)	30.9(22.3,39.4)	38.4(38.0,38.7)
Maharashtra	Nandurbar	54.5(45.9,63.1)	54.8(54.3,55.3)	10.4(6.3,16.9)	4.3(4.1,4.6)	36.8(26.2,47.3)	42.8(42.3,43.3)
Maharashtra	Nashik	39.4(26.6,52.3)	41.6(41.4,41.9)	6.2(3.1,12.1)	6.1(5.9,6.2)	33.1(21.7,44.5)	34.9(34.7,35.2)
Maharashtra	Osmanabad	56.6(46.9,66.3)	50.2(49.7,50.7)	10.2(6.0,16.8)	5.6(5.4,5.8)	37.6(31.3,43.9)	38.9(38.4,39.4)
Maharashtra	Parbhani	37.8(29.3,46.2)	45.0(44.5,45.5)	3.7(1.7,8.0)	3.3(3.1,3.5)	30.8(24.4,37.2)	32.6(32.1,33.1)
Maharashtra	Pune	37.8(30.2,45.4)	42.9(42.7,43.1)	3.0(1.1,7.5)	4.2(4.1,4.3)	30.6(25.3,35.9)	37.1(36.9,37.3)
Maharashtra	Raigarh	32.1(24.0,40.2)	37.4(37.0,37.7)	8.0(4.4,14.0)	4.5(4.3,4.7)	29.3(21.3,37.2)	31.5(31.1,31.8)
Maharashtra	Ratnagiri	39.6(25.4,53.8)	46.4(45.9,46.9)	7.0(3.5,13.4)	4.4(4.2,4.6)	31.9(19.7,44.1)	40.6(40.2,41.1)
Maharashtra	Sangli	32.5(25.1,39.9)	39.0(38.6,39.4)	2.4(0.9,6.3)	1.1(1.0,1.2)	26.7(18.0,35.4)	32.1(31.7,32.4)
Maharashtra	Satara	53.5(42.9,64.1)	44.3(43.9,44.6)	10.9(6.7,17.1)	5.4(5.3,5.6)	40.0(29.4,50.6)	38.4(38.0,38.7)
Maharashtra	Sindhudurg	38.0(28.7,47.4)	44.7(44.1,45.4)	6.1(2.5,14.0)	3.4(3.2,3.7)	30.8(26.6,35.1)	34.6(33.9,35.2)
Maharashtra	Solapur	50.5(36.0,65.0)	51.7(51.4,52.0)	5.3(2.5,10.8)	4.2(4.1,4.3)	44.3(30.8,57.8)	44.7(44.4,45.0)
Maharashtra	Thane	43.3(34.8,51.9)	38.1(37.9,38.3)	2.8(1.3,5.8)	3.5(3.4,3.5)	34.7(27.8,41.6)	34.6(34.4,34.8)
Maharashtra	Wardha	51.0(40.2,61.7)	42.4(41.9,43.0)	0.8(0.1,5.2)	3.0(2.8,3.2)	42.5(32.3,52.7)	37.4(36.9,38.0)
Maharashtra	Washim	55.6(47.8,63.3)	42.2(41.6,42.8)	0.7(0.1,4.7)	2.3(2.2,2.5)	39.1(31.7,46.6)	29.4(28.9,30.0)
Maharashtra	Yavatmal	53.7(43.3,64.2)	46.1(45.7,46.5)	0.0(0.0,0.0)	2.0(1.9,2.1)	48.1(36.3,59.8)	36.8(36.5,37.2)
Manipur	Bishnupur	57.6(47.1,68.0)	45.2(43.9,46.6)	18.2(12.7,25.3)	11.4(10.5,12.2)	47.3(37.6,56.9)	39.5(38.2,40.8)
Manipur	Chandel	53.6(44.6,62.6)	61.0(59.3,62.7)	11.0(6.0,19.4)	13.9(12.7,15.1)	45.8(34.6,57.0)	49.9(48.2,51.6)
Manipur	Churachandpur	41.8(32.1,51.5)	47.2(46.0,48.5)	10.5(6.1,17.4)	9.9(9.2,10.7)	29.7(21.0,38.4)	37.3(36.1,38.6)
Manipur	Imphal East	51.2(37.2,65.3)	40.9(40.0,41.9)	11.8(7.7,17.7)	11.6(11.0,12.2)	47.4(35.1,59.7)	35.1(34.2,36.0)
Manipur	Imphal West	41.9(34.9,49.0)	47.8(46.9,48.7)	7.6(4.3,13.0)	11.3(10.7,11.8)	36.7(31.6,41.8)	40.4(39.5,41.3)
Manipur	Senapati	39.6(30.7,48.5)	55.9(54.9,56.9)	6.6(3.0,14.0)	10.9(10.3,11.5)	31.0(20.8,41.2)	42.1(41.1,43.0)
Manipur	Tamenglong	52.5(45.2,59.8)	55.7(53.9,57.5)	8.0(4.3,14.3)	6.3(5.4,7.2)	39.8(31.9,47.8)	47.3(45.5,49.1)
Manipur	Thoubal	50.6(37.6,63.6)	41.2(40.2,42.2)	14.7(10.0,21.1)	10.3(9.7,11.0)	43.8(32.7,54.9)	34.8(33.8,35.8)
Manipur	Ukhrul	64.4(50.6,78.3)	58.2(56.6,59.7)	5.7(2.5,12.3)	8.5(7.6,9.3)	48.1(32.6,63.7)	43.3(41.7,44.8)
Meghalaya	East Garo Hills	41.5(33.3,49.8)	41.7(40.5,43.0)	5.4(3.5,8.3)	7.4(6.7,8.1)	30.7(24.8,36.6)	33.1(31.9,34.3)
Meghalaya	East Khasi Hills	47.9(41.0,54.8)	46.3(45.5,47.0)	15.1(10.4,21.5)	8.6(8.2,9.1)	42.3(35.3,49.3)	40.4(39.7,41.2)
Meghalaya	Jaintia Hills	53.5(47.0,59.9)	53.2(52.1,54.4)	9.1(6.3,12.9)	13.1(12.3,13.9)	46.0(38.8,53.3)	47.0(45.9,48.2)
Meghalaya	Ribhoi	60.1(47.3,72.8)	53.7(52.2,55.1)	9.6(6.0,15.0)	7.6(6.8,8.4)	52.7(40.9,64.6)	46.3(44.9,47.8)
Meghalaya	South Garo Hills	40.7(27.1,54.4)	48.3(46.3,50.2)	9.8(5.8,16.2)	6.3(5.4,7.3)	28.1(16.0,40.3)	35.6(33.8,37.5)
Meghalaya	West Garo Hills	40.4(30.6,50.3)	44.4(43.5,45.2)	6.9(3.6,12.9)	8.9(8.4,9.4)	32.0(20.4,43.6)	37.1(36.2,37.9)
Meghalaya	West Khasi Hills	51.9(44.8,59.0)	48.5(47.3,49.7)	5.6(3.4,9.1)	9.5(8.8,10.3)	44.8(40.6,49.0)	37.1(35.9,38.2)
Mizoram	Aizawl	33.6(25.1,42.0)	33.8(32.8,34.7)	20.1(14.2,27.7)	13.6(12.9,14.3)	31.5(23.9,39.1)	31.6(30.7,32.6)
Mizoram	Champhai	44.1(34.7,53.5)	38.1(36.3,40.0)	16.4(11.3,23.2)	15.8(14.4,17.1)	31.4(16.0,46.7)	30.6(28.9,32.4)
Mizoram	Kolasib	30.1(18.7,41.6)	36.6(34.3,38.8)	15.4(10.3,22.5)	15.8(14.1,17.5)	20.5(14.2,26.8)	28.9(26.8,31.1)
Mizoram	Lawngtlai	31.1(20.0,42.3)	39.5(37.5,41.6)	13.5(9.2,19.5)	12.7(11.3,14.1)	21.6(13.6,29.6)	28.5(26.6,30.4)
Mizoram	Lunglei	30.2(20.9,39.5)	32.1(30.5,33.7)	11.5(7.0,18.2)	12.2(11.1,13.3)	22.8(15.0,30.7)	27.4(25.9,29.0)
Mizoram	Mamit	42.9(34.0,51.8)	37.5(35.2,39.8)	7.8(4.5,13.0)	14.5(12.8,16.2)	36.8(25.7,48.0)	28.8(26.6,30.9)
Mizoram	Saiha	27.1(13.7,40.6)	35.7(32.9,38.4)	12.1(7.5,18.8)	12.6(10.7,14.6)	25.7(12.1,39.4)	23.9(21.5,26.4)
Mizoram	Serchhip	52.7(37.0,68.3)	38.5(35.9,41.0)	17.9(12.1,25.5)	17.5(15.5,19.5)	43.9(29.5,58.2)	34.4(31.9,36.9)
Nagaland	Dimapur	27.1(18.9,35.4)	36.0(35.0,37.1)	7.4(4.2,12.8)	9.9(9.2,10.5)	26.5(19.1,33.8)	23.4(22.4,24.3)
Nagaland	Kiphire	31.5(19.9,43.2)	38.9(36.3,41.5)	2.0(0.6,6.1)	3.8(2.7,4.8)	12.4(6.2,18.6)	14.4(12.5,16.2)
Nagaland	Kohima	42.8(35.4,50.1)	42.2(40.9,43.5)	9.6(5.6,15.9)	11.7(10.8,12.5)	32.6(23.7,41.5)	27.3(26.1,28.5)
Nagaland	Longleng	42.2(24.3,60.1)	39.4(36.3,42.5)	3.9(1.6,9.3)	4.1(2.8,5.3)	13.8(5.9,21.7)	15.1(12.8,17.4)
Nagaland	Mokokchung	33.5(23.4,43.7)	39.8(38.4,41.3)	10.3(5.9,17.4)	9.9(9.0,10.8)	23.9(16.7,31.1)	25.6(24.3,26.9)
Nagaland	Mon	41.6(27.0,56.2)	51.4(50.0,52.8)	7.4(4.0,13.4)	7.8(7.0,8.6)	23.4(11.0,35.7)	29.4(28.1,30.7)
Nagaland	Peren	46.2(33.3,59.1)	41.8(39.5,44.0)	5.8(2.6,12.3)	6.8(5.6,7.9)	20.2(14.3,26.1)	19.0(17.2,20.8)
Nagaland	Phek	54.4(45.9,62.9)	42.7(41.0,44.4)	10.1(6.0,16.5)	8.6(7.6,9.5)	25.4(16.8,33.9)	22.3(20.8,23.7)
Nagaland	Tuensang	42.6(24.3,60.8)	47.8(46.2,49.4)	7.9(4.4,13.8)	6.0(5.2,6.8)	11.2(6.1,16.3)	25.7(24.3,27.1)
Nagaland	Wokha	41.0(30.9,51.1)	30.9(29.4,32.4)	13.2(8.4,20.1)	8.1(7.2,8.9)	25.9(18.1,33.7)	16.0(14.8,17.2)
Nagaland	Zunheboto	43.4(35.6,51.3)	35.5(33.8,37.2)	6.1(3.1,11.6)	7.2(6.3,8.2)	20.0(8.0,32.0)	17.1(15.7,18.5)
NCT of Delhi	Central	19.4(11.5,27.4)	26.7(25.9,27.4)	4.1(1.8,8.8)	7.2(6.8,7.7)	18.8(11.2,26.4)	26.8(26.0,27.5)
NCT of Delhi	East	23.7(16.1,31.4)	26.5(26.0,26.9)	5.3(2.7,10.4)	7.9(7.6,8.1)	23.7(16.1,31.4)	26.1(25.7,26.5)
NCT of Delhi	New Delhi	22.1(13.7,30.6)	29.7(28.1,31.3)	5.7(2.6,12.1)	8.2(7.2,9.1)	22.1(13.7,30.6)	28.6(27.0,30.1)
NCT of Delhi	North	35.3(21.9,48.7)	23.1(22.5,23.7)	3.7(1.4,9.6)	3.7(3.4,4.0)	34.7(21.3,48.0)	23.6(23.0,24.1)
NCT of Delhi	North East	25.8(20.8,30.8)	26.0(25.6,26.4)	5.0(3.0,8.0)	5.9(5.7,6.1)	25.2(20.2,30.2)	24.6(24.2,25.0)
NCT of Delhi	North West	17.8(8.3,27.3)	23.6(23.3,23.9)	5.4(2.8,10.1)	4.0(3.9,4.2)	17.8(8.3,27.3)	23.2(22.9,23.5)
NCT of Delhi	South	16.6(11.3,21.9)	22.8(22.5,23.1)	3.5(2.0,6.1)	3.9(3.8,4.1)	16.3(10.9,21.7)	22.7(22.4,23.1)
NCT of Delhi	South West	36.4(27.5,45.4)	27.3(27.0,27.7)	15.6(10.5,22.7)	8.3(8.0,8.5)	34.1(24.1,44.1)	26.3(25.9,26.7)
NCT of Delhi	West	34.6(26.7,42.5)	26.2(25.8,26.6)	8.7(5.1,14.4)	7.9(7.7,8.2)	34.6(26.7,42.5)	25.7(25.4,26.1)
Odisha	Anugul	23.1(15.9,30.3)	20.4(19.9,20.8)	5.8(3.1,10.5)	3.7(3.5,3.9)	22.5(15.6,29.5)	18.3(17.8,18.7)
Odisha	Balangir	20.8(9.4,32.3)	37.4(36.9,37.9)	0.7(0.1,4.6)	3.8(3.6,4.0)	17.5(9.0,26.0)	32.3(31.8,32.8)
Odisha	Baleshwar	25.4(12.7,38.0)	25.7(25.4,26.1)	2.9(1.1,7.6)	5.3(5.1,5.5)	22.4(10.1,34.6)	21.0(20.7,21.4)
Odisha	Bargarh	41.7(30.2,53.2)	27.9(27.4,28.4)	4.0(1.7,9.3)	3.7(3.5,3.9)	35.3(22.9,47.7)	24.9(24.4,25.4)
Odisha	Baudh	37.2(29.1,45.3)	32.8(31.8,33.7)	6.0(3.3,10.9)	4.1(3.7,4.5)	26.0(17.5,34.6)	27.4(26.5,28.3)
Odisha	Bhadrak	11.8(3.9,19.6)	23.3(22.8,23.7)	3.6(1.5,8.4)	3.2(3.0,3.4)	9.5(4.5,14.6)	18.5(18.1,18.9)
Odisha	Cuttack	24.3(12.7,36.0)	24.4(24.0,24.7)	5.0(2.3,10.7)	4.0(3.9,4.2)	22.8(12.1,33.6)	24.5(24.1,24.8)
Odisha	Debagarh	37.6(27.7,47.6)	42.3(41.1,43.4)	2.8(1.1,7.3)	4.9(4.4,5.4)	34.5(23.9,45.1)	38.7(37.5,39.8)
Odisha	Dhenkanal	14.8(2.4,27.2)	29.4(28.9,30.0)	3.7(1.4,9.4)	5.9(5.7,6.2)	12.8(2.7,22.8)	23.4(22.9,23.9)
Odisha	Gajapati	42.6(32.9,52.3)	35.7(34.9,36.6)	2.2(0.7,6.7)	4.1(3.7,4.4)	40.1(31.0,49.3)	28.2(27.4,29.0)
Odisha	Ganjam	27.7(23.5,31.9)	23.8(23.5,24.1)	2.6(1.0,6.7)	5.2(5.1,5.4)	24.5(19.9,29.1)	21.3(21.0,21.5)
Odisha	Jagatsinghapur	15.0(6.0,24.0)	24.2(23.7,24.7)	2.2(0.7,6.5)	2.5(2.3,2.7)	14.4(5.9,22.9)	23.3(22.8,23.8)
Odisha	Jajapur	17.9(7.0,28.8)	24.8(24.4,25.3)	4.5(2.2,9.2)	4.8(4.6,5.0)	16.6(5.9,27.3)	22.7(22.3,23.1)
Odisha	Jharsuguda	26.6(18.4,34.7)	27.3(26.5,28.0)	0.0(0.0,0.0)	4.8(4.4,5.1)	21.2(14.8,27.6)	27.0(26.3,27.8)
Odisha	Kalahandi	36.7(23.0,50.3)	30.5(30.0,30.9)	10.5(6.4,16.7)	4.3(4.1,4.5)	33.8(20.9,46.8)	25.5(25.1,26.0)
Odisha	Kandhamal	27.9(17.1,38.7)	34.2(33.5,35.0)	6.0(3.0,11.5)	3.9(3.6,4.2)	25.3(15.5,35.2)	31.7(30.9,32.4)
Odisha	Kendrapara	12.7(6.9,18.4)	16.5(16.2,16.9)	4.3(2.1,8.9)	3.0(2.8,3.2)	10.2(5.0,15.4)	16.2(15.8,16.6)
Odisha	Kendujhar	35.1(21.3,49.0)	28.2(27.8,28.7)	6.2(3.2,11.8)	4.6(4.4,4.8)	32.4(19.8,45.1)	23.2(22.8,23.6)
Odisha	Khordha	29.4(19.6,39.2)	22.8(22.4,23.2)	4.4(1.8,10.1)	4.3(4.1,4.4)	27.1(17.3,36.9)	21.8(21.4,22.1)
Odisha	Koraput	48.3(32.8,63.7)	42.9(42.3,43.5)	8.0(4.6,13.6)	7.8(7.5,8.1)	42.3(27.6,57.0)	38.0(37.4,38.5)
Odisha	Malkangiri	46.5(35.4,57.7)	45.0(44.1,45.9)	7.3(4.2,12.4)	5.1(4.7,5.5)	37.0(28.7,45.4)	38.1(37.3,39.0)
Odisha	Mayurbhanj	35.9(23.7,48.1)	31.9(31.5,32.3)	5.8(2.9,11.2)	3.8(3.6,3.9)	35.9(23.7,48.1)	29.8(29.4,30.2)
Odisha	Nabarangapur	23.9(11.6,36.3)	33.9(33.3,34.4)	0.0(0.0,0.0)	6.4(6.1,6.7)	22.1(11.9,32.2)	31.2(30.7,31.8)
Odisha	Nayagarh	20.2(7.2,33.1)	26.5(25.9,27.1)	8.9(5.2,14.7)	5.4(5.1,5.7)	17.2(6.0,28.5)	21.1(20.5,21.6)
Odisha	Nuapada	46.0(30.4,61.6)	31.5(30.8,32.3)	0.8(0.1,5.2)	3.9(3.6,4.3)	42.3(28.4,56.3)	29.9(29.1,30.7)
Odisha	Puri	20.9(9.6,32.2)	22.7(22.3,23.1)	5.7(2.9,11.0)	3.7(3.5,3.9)	18.0(7.4,28.7)	24.0(23.6,24.4)
Odisha	Rayagada	55.0(46.3,63.7)	43.7(43.1,44.4)	13.5(8.8,20.1)	8.0(7.6,8.3)	43.6(35.8,51.4)	34.9(34.3,35.6)
Odisha	Sambalpur	44.6(36.5,52.7)	33.0(32.4,33.6)	6.6(3.5,12.2)	7.1(6.7,7.4)	41.7(33.4,50.1)	30.6(30.0,31.2)
Odisha	Subarnapur	25.7(19.5,31.8)	26.8(26.0,27.5)	1.4(0.4,5.6)	2.0(1.8,2.3)	20.5(14.9,26.2)	17.7(17.1,18.3)
Odisha	Sundargarh	31.1(21.5,40.6)	36.8(36.3,37.2)	2.8(1.1,7.3)	5.0(4.8,5.2)	29.3(20.6,38.0)	34.1(33.7,34.6)
Puducherry	Karaikal	28.3(20.6,35.9)	28.9(27.6,30.2)	4.5(1.0,18.4)	3.5(3.0,4.0)	28.3(20.6,35.9)	28.5(27.2,29.7)
Puducherry	Mahe	21.7(18.2,25.3)	18.1(15.8,20.3)	2.9(1.2,6.9)	2.1(1.2,2.9)	21.7(18.2,25.3)	17.8(15.5,20.0)
Puducherry	Puducherry	44.9(32.2,57.7)	37.4(36.8,38.0)	3.6(1.5,8.4)	3.5(3.2,3.7)	43.3(29.9,56.8)	36.3(35.7,36.9)
Puducherry	Yanam	21.0(13.5,28.4)	31.5(29.0,34.0)	0.5(0.1,3.5)	2.5(1.6,3.3)	20.2(12.8,27.6)	31.1(28.6,33.5)
Punjab	Amritsar	22.3(15.0,29.6)	21.2(20.9,21.6)	2.3(0.9,6.0)	1.7(1.6,1.8)	22.3(15.0,29.6)	21.5(21.1,21.8)
Punjab	Barnala	29.4(19.3,39.5)	22.9(22.1,23.6)	2.2(0.8,5.8)	1.5(1.3,1.7)	28.2(19.4,37.0)	21.2(20.5,21.8)
Punjab	Bathinda	25.3(15.5,35.2)	20.1(19.6,20.5)	3.9(1.9,8.1)	2.0(1.9,2.2)	21.9(12.0,31.9)	18.9(18.5,19.3)
Punjab	Faridkot	25.8(15.8,35.8)	24.8(24.1,25.6)	2.3(0.7,6.9)	1.6(1.4,1.8)	22.8(14.6,30.9)	22.5(21.8,23.2)
Punjab	Fatehgarh Sahib	20.6(7.5,33.7)	24.3(23.6,25.0)	1.5(0.3,6.4)	2.8(2.5,3.1)	20.1(6.9,33.2)	20.5(19.8,21.1)
Punjab	Firozpur	21.3(16.1,26.5)	24.1(23.7,24.5)	1.2(0.5,2.8)	2.2(2.0,2.3)	19.7(14.6,24.9)	22.2(21.8,22.6)
Punjab	Gurdaspur	22.6(11.1,34.1)	22.1(21.7,22.4)	4.1(1.6,9.9)	2.6(2.4,2.7)	16.0(6.6,25.4)	19.7(19.3,20.0)
Punjab	Hoshiarpur	21.1(14.3,27.8)	27.4(27.0,27.9)	4.6(2.3,9.0)	3.2(3.0,3.3)	19.3(12.4,26.2)	22.5(22.0,22.9)
Punjab	Jalandhar	30.5(21.8,39.3)	28.2(27.8,28.6)	2.2(0.7,6.7)	2.7(2.6,2.9)	28.3(19.6,37.0)	26.8(26.4,27.2)
Punjab	Kapurthala	21.2(15.2,27.2)	28.0(27.4,28.7)	0.6(0.1,4.2)	2.2(2.0,2.4)	20.0(14.2,25.8)	26.3(25.7,27.0)
Punjab	Ludhiana	30.2(23.5,36.8)	25.1(24.8,25.4)	1.1(0.3,4.4)	2.4(2.3,2.5)	23.9(17.8,30.0)	23.5(23.2,23.8)
Punjab	Mansa	20.2(15.0,25.5)	23.3(22.7,24.0)	3.5(1.6,7.5)	3.5(3.2,3.7)	20.2(15.0,25.5)	21.7(21.1,22.3)
Punjab	Moga	30.4(23.3,37.5)	27.4(26.8,28.0)	4.3(2.2,8.4)	2.0(1.8,2.2)	29.8(22.3,37.4)	24.0(23.4,24.6)
Punjab	Muktsar	29.5(20.8,38.2)	27.4(26.8,28.0)	2.5(0.9,6.5)	3.0(2.8,3.2)	28.1(18.5,37.7)	25.4(24.8,26.0)
Punjab	Patiala	32.5(25.3,39.7)	24.1(23.7,24.5)	3.0(1.2,7.0)	3.1(2.9,3.2)	28.9(24.2,33.7)	20.1(19.7,20.5)
Punjab	Rupnagar	27.2(18.4,35.9)	24.8(24.1,25.5)	2.3(0.7,7.0)	2.8(2.6,3.1)	20.1(10.8,29.4)	20.5(19.8,21.1)
Punjab	Sahibzada Ajit Singh Nagar	20.2(11.3,29.2)	16.6(16.1,17.1)	0.0(0.0,0.0)	1.3(1.1,1.4)	20.2(11.3,29.2)	15.7(15.2,16.2)
Punjab	Sangrur	15.4(6.6,24.1)	19.4(19.0,19.8)	1.7(0.4,6.7)	1.9(1.8,2.1)	14.7(5.8,23.6)	18.0(17.6,18.4)
Punjab	Shahid Bhagat Singh Nagar	16.8(10.9,22.7)	28.9(28.2,29.6)	1.2(0.3,4.6)	2.3(2.0,2.5)	16.8(10.9,22.7)	26.5(25.8,27.2)
Punjab	Taran Taran	21.4(15.3,27.5)	23.7(23.1,24.2)	2.1(0.7,6.3)	2.0(1.8,2.2)	19.2(13.2,25.2)	23.2(22.7,23.8)
Rajasthan	Ajmer	25.6(14.2,37.0)	24.8(24.5,25.2)	2.5(0.9,6.5)	3.2(3.0,3.3)	12.1(5.2,19.1)	17.1(16.8,17.4)
Rajasthan	Alwar	18.3(10.9,25.6)	26.1(25.8,26.4)	4.2(2.0,8.6)	3.2(3.1,3.3)	15.5(8.6,22.3)	16.9(16.6,17.1)
Rajasthan	Banswara	54.7(44.1,65.3)	37.1(36.6,37.6)	1.0(0.3,4.1)	1.7(1.6,1.8)	12.7(6.8,18.6)	15.9(15.5,16.3)
Rajasthan	Baran	39.8(32.3,47.3)	34.1(33.5,34.7)	5.1(2.9,8.8)	2.9(2.7,3.1)	22.7(14.1,31.3)	20.6(20.1,21.1)
Rajasthan	Barmer	16.6(7.5,25.8)	20.8(20.4,21.2)	1.3(0.4,3.8)	2.5(2.3,2.6)	8.9(1.2,16.6)	11.0(10.8,11.3)
Rajasthan	Bharatpur	27.9(16.0,39.8)	22.9(22.6,23.3)	1.6(0.5,4.8)	2.6(2.5,2.8)	21.9(10.0,33.8)	14.1(13.8,14.4)
Rajasthan	Bhilwara	46.7(35.8,57.6)	36.1(35.7,36.6)	5.6(3.0,10.2)	4.0(3.9,4.2)	33.2(22.3,44.0)	24.9(24.5,25.3)
Rajasthan	Bikaner	21.8(10.4,33.2)	22.9(22.5,23.3)	3.3(1.7,6.2)	3.2(3.0,3.4)	14.0(4.1,23.9)	16.3(16.0,16.6)
Rajasthan	Bundi	34.8(23.8,45.8)	37.2(36.6,37.8)	6.5(3.9,10.6)	5.9(5.6,6.2)	27.9(19.2,36.5)	21.2(20.6,21.7)
Rajasthan	Chittaurgarh	45.1(32.7,57.5)	31.1(30.6,31.6)	1.9(0.6,5.9)	3.4(3.2,3.6)	18.1(11.6,24.6)	16.9(16.5,17.3)
Rajasthan	Churu	21.6(10.2,33.0)	25.3(24.9,25.7)	2.1(0.8,5.5)	2.2(2.0,2.3)	9.7(4.6,14.8)	15.3(14.9,15.6)
Rajasthan	Dausa	26.6(17.9,35.3)	30.2(29.7,30.7)	2.1(0.8,5.4)	3.4(3.2,3.6)	16.6(7.7,25.4)	17.0(16.6,17.4)
Rajasthan	Dhaulpur	14.6(7.2,21.9)	17.4(16.8,17.9)	3.0(1.2,7.1)	1.9(1.8,2.1)	8.3(3.4,13.3)	9.1(8.7,9.5)
Rajasthan	Dungarpur	24.2(13.1,35.2)	38.6(38.1,39.2)	1.9(0.7,4.9)	2.8(2.7,3.0)	10.0(4.5,15.4)	20.4(20.0,20.9)
Rajasthan	Ganganagar	23.1(16.2,30.1)	28.7(28.3,29.1)	2.8(1.3,6.1)	2.7(2.5,2.9)	17.5(11.2,23.7)	18.8(18.4,19.2)
Rajasthan	Hanumangarh	28.4(19.7,37.1)	25.8(25.4,26.3)	1.7(0.6,4.5)	2.4(2.2,2.5)	21.5(16.0,27.1)	17.4(17.0,17.8)
Rajasthan	Jaipur	33.4(20.5,46.2)	23.4(23.2,23.6)	3.0(1.4,6.2)	3.8(3.7,3.9)	23.9(13.1,34.8)	15.3(15.1,15.5)
Rajasthan	Jaisalmer	4.4(-0.1,8.9)	21.9(21.1,22.6)	1.2(0.4,3.7)	2.3(2.1,2.6)	3.9(-0.5,8.4)	13.3(12.7,14.0)
Rajasthan	Jalor	28.7(18.0,39.3)	34.9(34.4,35.4)	12.0(6.7,20.6)	5.6(5.4,5.8)	13.9(4.4,23.4)	18.8(18.4,19.3)
Rajasthan	Jhalawar	31.3(20.4,42.2)	31.9(31.4,32.4)	4.3(2.2,8.5)	2.7(2.5,2.9)	22.2(14.1,30.4)	19.1(18.6,19.5)
Rajasthan	Jhunjhunun	10.8(5.1,16.4)	21.3(20.9,21.6)	1.6(0.5,4.7)	3.9(3.8,4.1)	5.4(0.2,10.6)	12.8(12.5,13.1)
Rajasthan	Jodhpur	25.7(19.7,31.6)	26.1(25.8,26.4)	2.5(1.1,5.5)	5.5(5.4,5.7)	12.6(6.4,18.9)	16.9(16.7,17.2)
Rajasthan	Karauli	32.6(18.5,46.7)	25.2(24.7,25.7)	5.3(2.8,10.0)	4.6(4.3,4.8)	20.4(6.0,34.8)	14.3(13.9,14.7)
Rajasthan	Kota	23.0(14.0,32.0)	24.8(24.4,25.3)	5.8(3.2,10.1)	3.2(3.0,3.4)	19.9(10.3,29.5)	19.7(19.4,20.1)
Rajasthan	Nagaur	28.0(18.8,37.1)	24.9(24.5,25.2)	1.5(0.5,3.9)	2.3(2.2,2.4)	16.0(10.2,21.7)	15.7(15.4,15.9)
Rajasthan	Pali	20.6(13.8,27.5)	30.5(30.1,31.0)	4.1(2.0,8.4)	2.8(2.6,2.9)	15.8(8.8,22.9)	19.2(18.8,19.6)
Rajasthan	Pratapgarh	46.3(29.7,62.8)	38.3(37.6,39.0)	7.7(4.5,12.9)	2.9(2.6,3.1)	16.4(9.4,23.3)	20.8(20.2,21.4)
Rajasthan	Rajsamand	33.5(23.6,43.4)	28.1(27.5,28.7)	1.8(0.6,5.6)	3.0(2.8,3.2)	22.6(10.3,34.9)	18.2(17.7,18.7)
Rajasthan	Sawai Madhopur	28.7(13.7,43.7)	34.9(34.3,35.5)	2.2(0.8,5.7)	3.7(3.5,3.9)	13.1(5.2,21.1)	18.1(17.7,18.6)
Rajasthan	Sikar	24.7(19.1,30.3)	24.5(24.1,24.8)	3.3(1.6,6.7)	4.3(4.1,4.5)	18.7(12.8,24.6)	14.9(14.6,15.2)
Rajasthan	Sirohi	50.1(41.9,58.4)	37.6(37.0,38.3)	1.4(0.4,4.2)	4.8(4.5,5.1)	35.8(25.4,46.3)	24.6(24.0,25.1)
Rajasthan	Tonk	28.2(21.0,35.5)	32.4(31.9,33.0)	4.7(2.5,8.8)	2.8(2.6,3.0)	16.3(10.5,22.2)	20.4(19.9,20.9)
Rajasthan	Udaipur	37.8(23.6,52.0)	37.7(37.3,38.1)	1.6(0.5,4.8)	4.6(4.4,4.7)	28.3(17.3,39.4)	20.8(20.5,21.2)
Sikkim	East District	42.7(29.2,56.3)	35.7(34.5,36.9)	22.2(13.9,33.4)	19.8(18.8,20.8)	35.2(26.4,44.0)	33.1(31.9,34.2)
Sikkim	North	37.5(27.2,47.7)	42.3(38.9,45.7)	14.3(8.9,22.3)	16.7(14.1,19.2)	32.9(25.0,40.8)	32.4(29.2,35.6)
Sikkim	South District	37.1(28.1,46.1)	34.7(33.0,36.3)	9.0(4.6,17.0)	11.1(10.0,12.2)	29.8(22.5,37.2)	30.9(29.3,32.5)
Sikkim	West District	30.0(19.6,40.5)	34.6(32.9,36.4)	9.4(5.7,15.0)	7.2(6.3,8.2)	28.6(17.9,39.3)	30.1(28.5,31.8)
Tamil Nadu	Ariyalur	42.3(33.0,51.6)	43.5(42.8,44.2)	3.0(1.1,7.8)	3.0(2.7,3.2)	39.7(30.0,49.4)	41.3(40.6,42.0)
Tamil Nadu	Chennai	26.9(15.2,38.6)	31.0(30.8,31.3)	2.1(0.5,7.9)	2.5(2.5,2.6)	26.9(15.2,38.6)	31.1(30.8,31.3)
Tamil Nadu	Coimbatore	44.6(32.6,56.7)	46.6(46.3,46.9)	4.4(1.7,10.9)	4.5(4.4,4.6)	43.6(31.4,55.8)	45.5(45.2,45.8)
Tamil Nadu	Cuddalore	40.3(29.0,51.6)	46.4(46.0,46.8)	4.9(2.0,11.2)	4.8(4.6,4.9)	37.5(24.0,50.9)	42.3(42.0,42.7)
Tamil Nadu	Dharmapuri	45.7(36.2,55.2)	41.8(41.3,42.4)	1.8(0.5,6.0)	3.1(2.9,3.3)	42.8(31.8,53.8)	40.7(40.2,41.3)
Tamil Nadu	Dindigul	49.3(38.6,60.1)	42.3(41.8,42.7)	5.6(2.7,11.3)	3.9(3.7,4.0)	48.4(38.3,58.6)	41.4(41.0,41.8)
Tamil Nadu	Erode	38.9(29.8,48.1)	47.0(46.6,47.5)	0.3(0.0,2.4)	5.6(5.4,5.8)	38.9(29.8,48.1)	43.9(43.5,44.3)
Tamil Nadu	Kancheepuram	41.2(33.1,49.3)	44.5(44.2,44.8)	10.8(5.9,18.9)	4.1(4.0,4.3)	37.0(28.5,45.5)	41.7(41.4,42.0)
Tamil Nadu	Kanniyakumari	28.2(20.6,35.7)	29.3(28.9,29.7)	7.6(3.8,14.5)	4.8(4.6,5.0)	24.2(18.9,29.5)	29.8(29.4,30.2)
Tamil Nadu	Karur	55.8(44.1,67.4)	51.5(50.9,52.1)	0.0(0.0,0.0)	3.9(3.6,4.1)	53.7(41.1,66.2)	48.2(47.6,48.8)
Tamil Nadu	Krishnagiri	43.3(31.1,55.5)	39.8(39.3,40.2)	3.7(1.6,8.0)	3.4(3.2,3.5)	41.2(28.2,54.1)	39.0(38.5,39.4)
Tamil Nadu	Madurai	33.2(22.5,43.9)	37.1(36.7,37.4)	5.1(2.4,10.3)	4.5(4.4,4.7)	29.5(19.8,39.1)	35.8(35.5,36.2)
Tamil Nadu	Nagapattinam	47.5(39.9,55.1)	42.3(41.8,42.7)	0.9(0.1,6.1)	3.2(3.0,3.4)	46.8(39.4,54.1)	41.9(41.5,42.4)
Tamil Nadu	Namakkal	41.2(33.7,48.6)	48.2(47.7,48.6)	9.0(4.7,16.5)	5.3(5.1,5.5)	38.1(29.9,46.3)	44.1(43.6,44.5)
Tamil Nadu	Perambalur	54.1(43.0,65.2)	52.4(51.6,53.2)	3.5(1.3,9.0)	5.1(4.7,5.4)	50.2(39.7,60.7)	51.1(50.3,51.9)
Tamil Nadu	Pudukkottai	50.1(42.1,58.0)	49.2(48.7,49.6)	11.9(7.0,19.7)	7.0(6.7,7.2)	48.8(40.0,57.6)	46.1(45.6,46.6)
Tamil Nadu	Ramanathapuram	40.7(34.8,46.5)	36.6(36.1,37.1)	3.3(1.3,7.9)	2.2(2.1,2.4)	38.9(32.3,45.4)	35.9(35.4,36.4)
Tamil Nadu	Salem	41.0(32.1,50.0)	40.9(40.5,41.2)	4.0(1.7,9.3)	5.9(5.7,6.0)	39.4(30.6,48.2)	37.4(37.1,37.7)
Tamil Nadu	Sivaganga	39.6(28.3,51.0)	47.3(46.8,47.8)	0.8(0.1,5.2)	3.9(3.7,4.1)	37.6(27.1,48.1)	41.9(41.3,42.4)
Tamil Nadu	Thanjavur	33.1(25.6,40.5)	37.4(37.0,37.8)	2.2(0.7,7.1)	2.5(2.4,2.6)	32.3(25.5,39.1)	36.9(36.5,37.3)
Tamil Nadu	The Nilgiris	59.3(50.7,67.9)	50.2(49.5,50.9)	3.0(1.1,7.8)	4.2(3.9,4.5)	52.9(42.4,63.3)	44.9(44.2,45.6)
Tamil Nadu	Theni	43.7(34.3,53.0)	48.2(47.7,48.8)	2.3(0.8,5.9)	3.8(3.5,4.0)	41.4(30.9,51.8)	45.5(44.9,46.0)
Tamil Nadu	Thiruvallur	39.9(32.4,47.5)	45.9(45.6,46.2)	5.7(2.8,11.1)	5.0(4.8,5.1)	39.1(31.2,46.9)	43.4(43.1,43.8)
Tamil Nadu	Thiruvarur	51.8(41.2,62.4)	52.5(52.0,53.1)	5.6(2.7,11.4)	4.8(4.6,5.0)	49.0(38.2,59.7)	51.2(50.7,51.8)
Tamil Nadu	Thoothukkudi	39.2(23.8,54.7)	42.3(41.9,42.8)	1.3(0.3,5.5)	2.9(2.8,3.1)	36.7(22.5,50.9)	40.5(40.1,41.0)
Tamil Nadu	Tiruchirappalli	50.3(38.8,61.9)	46.6(46.3,47.0)	5.3(2.2,12.2)	3.7(3.5,3.8)	48.4(37.2,59.5)	44.3(44.0,44.7)
Tamil Nadu	Tirunelveli	47.4(35.3,59.5)	38.5(38.2,38.9)	3.8(1.4,9.7)	5.6(5.4,5.8)	46.4(33.1,59.7)	35.8(35.5,36.2)
Tamil Nadu	Tiruppur	60.0(48.3,71.7)	45.8(45.4,46.2)	1.7(0.4,6.8)	4.2(4.1,4.4)	60.0(48.3,71.7)	43.5(43.1,43.9)
Tamil Nadu	Tiruvannamalai	49.1(39.0,59.2)	49.2(48.8,49.6)	8.9(4.3,17.5)	4.9(4.8,5.1)	49.1(39.0,59.2)	47.7(47.3,48.1)
Tamil Nadu	Vellore	47.4(40.2,54.5)	45.8(45.5,46.1)	3.0(0.7,11.0)	3.6(3.5,3.7)	44.8(36.7,52.9)	42.6(42.3,42.9)
Tamil Nadu	Viluppuram	46.1(37.4,54.7)	47.4(47.0,47.7)	3.3(1.2,8.4)	3.2(3.1,3.3)	40.9(30.8,51.0)	45.1(44.7,45.4)
Tamil Nadu	Virudhunagar	43.8(39.0,48.6)	47.4(47.0,47.9)	6.1(2.9,12.4)	5.8(5.6,6.0)	42.3(38.3,46.4)	45.6(45.2,46.1)
Telangana	Adilabad	48.9(42.1,55.7)	51.7(51.3,52.1)	2.3(1.3,4.0)	3.3(3.1,3.4)	44.4(37.7,51.1)	49.3(48.9,49.7)
Telangana	Hyderabad	18.7(9.3,28.1)	36.0(35.7,36.3)	0.9(0.1,5.8)	2.5(2.4,2.6)	16.6(9.5,23.7)	37.0(36.7,37.3)
Telangana	Karimnagar	52.1(46.7,57.6)	50.8(50.5,51.1)	4.6(3.1,6.8)	3.1(3.0,3.3)	49.8(44.4,55.2)	45.1(44.7,45.4)
Telangana	Khammam	48.2(38.9,57.5)	47.9(47.5,48.3)	3.0(1.4,6.2)	2.4(2.3,2.5)	46.4(37.3,55.4)	43.0(42.6,43.4)
Telangana	Mahbubnagar	59.6(54.4,64.9)	54.6(54.2,54.9)	2.4(1.4,4.2)	2.8(2.7,2.9)	55.2(50.1,60.3)	50.2(49.8,50.5)
Telangana	Medak	47.4(40.2,54.7)	46.8(46.5,47.2)	2.8(1.5,5.2)	2.7(2.6,2.8)	43.4(36.0,50.7)	42.5(42.1,42.9)
Telangana	Nalgonda	57.8(52.9,62.7)	54.9(54.5,55.2)	3.4(2.0,5.7)	4.1(3.9,4.2)	52.9(46.1,59.7)	50.4(50.1,50.7)
Telangana	Nizamabad	54.6(45.2,64.1)	58.7(58.3,59.1)	3.6(1.6,7.8)	3.7(3.5,3.8)	51.6(42.6,60.6)	55.9(55.5,56.3)
Telangana	Rangareddy	45.6(39.6,51.6)	36.5(36.2,36.7)	5.3(3.2,8.8)	3.2(3.1,3.3)	42.9(37.3,48.4)	34.9(34.6,35.2)
Telangana	Warangal	56.1(50.3,61.9)	51.3(50.9,51.6)	3.0(1.8,4.7)	3.4(3.3,3.5)	53.9(47.6,60.3)	48.8(48.4,49.1)
Tripura	Dhalai	33.6(27.4,39.8)	33.1(32.1,34.2)	0.6(0.1,4.1)	1.3(1.1,1.6)	32.4(25.7,39.1)	27.8(26.8,28.8)
Tripura	North Tripura	21.9(16.6,27.3)	24.7(24.0,25.4)	2.6(1.3,5.1)	1.9(1.7,2.1)	18.6(13.4,23.7)	20.1(19.5,20.8)
Tripura	South Tripura	33.1(24.0,42.2)	29.6(29.0,30.2)	0.9(0.2,3.7)	2.1(1.9,2.3)	23.5(14.9,32.1)	23.3(22.7,23.8)
Tripura	West Tripura	32.7(25.2,40.1)	33.8(33.4,34.3)	4.1(2.3,7.4)	3.0(2.8,3.1)	22.4(17.0,27.9)	25.7(25.3,26.2)
Uttar Pradesh	Agra	24.8(19.4,30.3)	19.1(18.8,19.3)	5.1(2.5,9.9)	3.1(3.0,3.2)	23.0(16.7,29.2)	16.5(16.2,16.7)
Uttar Pradesh	Aligarh	26.1(17.1,35.0)	29.6(29.3,30.0)	4.5(2.1,9.3)	4.4(4.2,4.5)	22.8(13.2,32.4)	24.6(24.3,25.0)
Uttar Pradesh	Allahabad	31.0(21.5,40.6)	21.0(20.7,21.2)	4.0(1.7,9.0)	3.5(3.4,3.6)	25.8(15.5,36.0)	16.4(16.2,16.6)
Uttar Pradesh	Ambedkar Nagar	24.6(13.9,35.4)	20.6(20.2,20.9)	2.8(1.2,6.5)	2.2(2.1,2.3)	15.2(8.7,21.8)	13.7(13.4,14.0)
Uttar Pradesh	Auraiya	23.1(12.5,33.6)	21.7(21.2,22.2)	1.1(0.3,4.2)	2.4(2.2,2.6)	18.1(10.2,26.1)	15.7(15.2,16.1)
Uttar Pradesh	Azamgarh	22.7(12.8,32.7)	14.8(14.5,15.0)	2.4(0.9,6.3)	2.2(2.1,2.2)	12.7(4.6,20.7)	10.0(9.8,10.2)
Uttar Pradesh	Baghpat	16.8(9.0,24.6)	19.8(19.3,20.3)	3.3(1.5,7.1)	2.0(1.9,2.2)	13.3(7.1,19.5)	15.4(14.9,15.8)
Uttar Pradesh	Bahraich	31.5(15.9,47.1)	17.5(17.2,17.7)	1.2(0.3,4.6)	1.3(1.2,1.4)	16.3(5.8,26.7)	9.1(8.9,9.3)
Uttar Pradesh	Ballia	12.2(3.3,21.2)	20.5(20.2,20.8)	1.7(0.5,5.1)	2.4(2.3,2.5)	10.0(1.9,18.0)	16.5(16.2,16.8)
Uttar Pradesh	Balrampur	24.2(13.0,35.5)	19.8(19.4,20.2)	0.5(0.1,3.8)	2.1(1.9,2.2)	14.7(6.4,23.0)	12.3(12.0,12.7)
Uttar Pradesh	Banda	36.0(22.5,49.6)	26.7(26.2,27.2)	2.6(1.1,6.1)	4.6(4.4,4.9)	17.1(8.7,25.4)	16.7(16.3,17.1)
Uttar Pradesh	Bara Banki	24.9(13.2,36.7)	23.1(22.7,23.4)	3.7(1.8,7.6)	2.9(2.7,3.0)	19.0(8.2,29.8)	16.2(15.9,16.5)
Uttar Pradesh	Bareilly	13.3(7.2,19.5)	21.5(21.2,21.8)	2.9(1.2,6.8)	3.7(3.6,3.8)	9.6(5.0,14.2)	15.1(14.9,15.3)
Uttar Pradesh	Basti	12.1(7.7,16.6)	15.1(14.7,15.4)	1.9(0.7,5.0)	2.6(2.4,2.7)	10.2(6.0,14.4)	10.7(10.4,10.9)
Uttar Pradesh	Bijnor	19.7(14.1,25.4)	19.5(19.2,19.8)	3.6(1.7,7.4)	3.7(3.5,3.8)	16.7(10.8,22.5)	15.1(14.8,15.4)
Uttar Pradesh	Budaun	24.5(11.4,37.7)	18.4(18.1,18.7)	3.1(1.4,6.7)	2.9(2.7,3.0)	17.1(9.4,24.9)	13.3(13.1,13.6)
Uttar Pradesh	Bulandshahr	21.1(13.8,28.3)	22.1(21.8,22.4)	2.5(0.9,6.6)	3.5(3.3,3.6)	18.2(11.9,24.6)	17.6(17.4,17.9)
Uttar Pradesh	Chandauli	12.0(4.8,19.2)	20.6(20.2,21.0)	1.4(0.4,4.2)	2.1(2.0,2.2)	8.2(4.2,12.1)	12.5(12.2,12.8)
Uttar Pradesh	Chitrakoot	24.9(13.7,36.1)	22.8(22.2,23.4)	1.1(0.3,4.4)	2.3(2.1,2.6)	19.9(11.3,28.4)	16.2(15.6,16.7)
Uttar Pradesh	Deoria	16.5(8.5,24.5)	17.9(17.6,18.2)	2.1(0.8,5.5)	2.5(2.4,2.6)	9.9(5.0,14.8)	13.6(13.3,13.9)
Uttar Pradesh	Etah	21.4(13.9,28.9)	20.5(20.0,20.9)	0.6(0.1,4.1)	2.3(2.1,2.4)	6.6(2.9,10.4)	12.5(12.2,12.9)
Uttar Pradesh	Etawah	14.5(9.7,19.3)	18.7(18.3,19.1)	3.8(1.8,7.8)	2.7(2.6,2.9)	12.2(7.2,17.1)	15.2(14.8,15.6)
Uttar Pradesh	Faizabad	17.7(9.8,25.6)	15.7(15.4,16.0)	1.3(0.4,3.9)	2.8(2.7,3.0)	11.0(7.0,15.1)	11.6(11.3,11.8)
Uttar Pradesh	Farrukhabad	29.1(14.6,43.7)	20.6(20.1,21.0)	3.6(1.7,7.3)	3.8(3.6,4.0)	15.9(5.4,26.4)	15.3(14.9,15.7)
Uttar Pradesh	Fatehpur	21.3(11.7,30.9)	24.0(23.6,24.4)	0.7(0.1,4.5)	2.9(2.8,3.1)	15.2(10.5,19.9)	16.6(16.3,16.9)
Uttar Pradesh	Firozabad	26.0(12.6,39.3)	23.4(23.1,23.8)	5.6(3.1,10.0)	4.1(3.9,4.3)	22.1(9.7,34.5)	19.2(18.9,19.6)
Uttar Pradesh	Gautam Buddha Nagar	28.8(17.6,40.0)	23.9(23.5,24.4)	9.0(3.8,20.1)	3.6(3.4,3.8)	26.3(14.1,38.5)	20.8(20.3,21.2)
Uttar Pradesh	Ghaziabad	18.7(10.6,26.9)	18.5(18.3,18.8)	0.8(0.4,1.9)	2.9(2.8,3.0)	18.1(9.8,26.4)	17.2(17.0,17.4)
Uttar Pradesh	Ghazipur	22.2(14.0,30.4)	19.9(19.6,20.2)	3.4(1.6,6.9)	2.1(2.0,2.2)	14.7(7.9,21.4)	13.4(13.2,13.7)
Uttar Pradesh	Gonda	17.3(11.4,23.2)	16.1(15.9,16.4)	1.6(0.5,5.0)	0.8(0.7,0.9)	9.5(4.5,14.4)	9.4(9.2,9.6)
Uttar Pradesh	Gorakhpur	24.5(11.6,37.4)	23.6(23.3,23.8)	0.9(0.2,3.6)	3.4(3.3,3.5)	12.8(6.1,19.5)	15.6(15.3,15.8)
Uttar Pradesh	Hamirpur	26.2(16.3,36.2)	23.8(23.2,24.4)	2.8(1.3,6.1)	2.6(2.4,2.9)	14.2(8.0,20.4)	16.8(16.3,17.3)
Uttar Pradesh	Hardoi	24.9(15.0,34.7)	24.3(24.0,24.6)	5.1(2.8,9.2)	2.8(2.7,2.9)	17.5(9.8,25.2)	15.7(15.4,16.0)
Uttar Pradesh	Jalaun	17.4(10.4,24.5)	23.8(23.3,24.2)	0.9(0.2,3.3)	1.8(1.7,2.0)	14.0(7.7,20.3)	17.5(17.1,17.9)
Uttar Pradesh	Jaunpur	21.7(11.0,32.5)	21.0(20.7,21.2)	1.3(0.4,3.9)	1.5(1.4,1.6)	14.3(5.3,23.3)	14.4(14.2,14.6)
Uttar Pradesh	Jhansi	24.1(15.2,33.0)	22.4(22.0,22.8)	2.9(1.4,5.9)	2.6(2.4,2.7)	15.3(5.2,25.4)	17.9(17.5,18.2)
Uttar Pradesh	Jyotiba Phule Nagar	20.7(13.6,27.8)	19.7(19.2,20.1)	5.8(3.4,9.7)	2.8(2.6,2.9)	15.4(8.1,22.7)	15.3(14.9,15.7)
Uttar Pradesh	Kannauj	19.4(8.1,30.6)	21.7(21.3,22.2)	2.0(0.7,5.2)	2.8(2.6,3.0)	17.4(7.6,27.3)	14.9(14.5,15.3)
Uttar Pradesh	Kanpur Dehat	23.1(12.3,34.0)	22.2(21.8,22.6)	4.9(2.4,9.5)	3.5(3.3,3.7)	16.8(7.9,25.6)	16.4(16.0,16.8)
Uttar Pradesh	Kanpur Nagar	15.9(7.9,23.9)	18.4(18.2,18.7)	4.2(2.0,8.5)	4.2(4.1,4.3)	15.3(8.0,22.6)	16.0(15.8,16.2)
Uttar Pradesh	Kanshiram Nagar	26.0(17.1,34.9)	21.6(21.1,22.1)	5.3(2.9,9.7)	2.5(2.3,2.6)	18.3(11.6,25.1)	16.7(16.2,17.1)
Uttar Pradesh	Kaushambi	25.7(16.2,35.2)	24.7(24.2,25.1)	0.7(0.1,4.6)	3.4(3.2,3.6)	15.5(9.4,21.6)	16.2(15.7,16.6)
Uttar Pradesh	Kheri	24.2(16.7,31.6)	21.8(21.5,22.1)	7.1(4.0,12.1)	3.6(3.5,3.7)	21.4(15.5,27.3)	14.6(14.3,14.8)
Uttar Pradesh	Kushinagar	15.2(10.5,19.9)	16.8(16.6,17.1)	2.2(0.8,5.9)	0.8(0.8,0.9)	12.6(8.0,17.3)	9.6(9.4,9.8)
Uttar Pradesh	Lalitpur	21.6(12.1,31.1)	24.0(23.4,24.5)	1.7(0.6,4.5)	2.5(2.3,2.7)	16.4(8.9,24.0)	16.8(16.4,17.3)
Uttar Pradesh	Lucknow	29.2(18.7,39.7)	22.5(22.3,22.8)	5.1(2.6,9.6)	3.4(3.3,3.5)	27.6(18.2,37.0)	20.0(19.8,20.3)
Uttar Pradesh	Mahamaya Nagar	22.0(15.9,28.2)	23.2(22.8,23.7)	4.3(2.1,8.3)	3.3(3.1,3.5)	18.6(14.0,23.2)	19.2(18.7,19.6)
Uttar Pradesh	Mahoba	26.1(14.6,37.6)	22.4(21.7,23.0)	2.0(0.7,5.1)	2.9(2.6,3.1)	18.7(10.3,27.1)	15.9(15.4,16.5)
Uttar Pradesh	Mahrajganj	10.9(3.2,18.6)	17.6(17.3,17.9)	0.5(0.1,3.7)	0.8(0.7,0.9)	6.1(3.0,9.2)	10.0(9.7,10.2)
Uttar Pradesh	Mainpuri	21.3(10.9,31.6)	26.4(26.0,26.9)	4.3(2.3,8.0)	2.7(2.6,2.9)	9.7(5.2,14.3)	19.0(18.6,19.4)
Uttar Pradesh	Mathura	27.2(16.1,38.3)	18.8(18.4,19.1)	5.6(3.0,10.2)	2.5(2.4,2.6)	21.6(12.4,30.8)	15.6(15.3,15.9)
Uttar Pradesh	Mau	10.5(4.9,16.2)	16.1(15.7,16.4)	1.0(0.2,3.8)	2.2(2.0,2.3)	7.3(2.5,12.2)	10.9(10.7,11.2)
Uttar Pradesh	Meerut	23.9(16.7,31.1)	17.5(17.3,17.8)	4.9(2.5,9.3)	3.0(2.9,3.1)	22.6(14.8,30.5)	16.0(15.7,16.2)
Uttar Pradesh	Mirzapur	23.0(11.6,34.5)	18.5(18.2,18.9)	2.1(0.8,5.5)	4.9(4.7,5.1)	18.3(11.4,25.2)	14.3(14.0,14.6)
Uttar Pradesh	Moradabad	15.1(9.2,21.1)	17.9(17.6,18.1)	2.0(0.9,4.2)	4.0(3.9,4.2)	10.9(6.6,15.2)	14.1(13.9,14.3)
Uttar Pradesh	Muzaffarpur	15.5(9.5,21.4)	19.5(19.2,19.8)	1.1(0.4,2.8)	3.1(3.0,3.2)	12.4(6.3,18.6)	14.8(14.5,15.0)
Uttar Pradesh	Pilibhit	16.5(8.4,24.5)	22.4(22.0,22.8)	0.6(0.1,4.4)	3.0(2.8,3.1)	13.9(4.7,23.0)	19.1(18.7,19.5)
Uttar Pradesh	Pratapgarh	14.5(4.6,24.5)	28.8(28.4,29.1)	0.9(0.2,3.5)	2.9(2.8,3.0)	10.0(3.4,16.5)	20.4(20.1,20.7)
Uttar Pradesh	Rae Bareli	19.7(9.8,29.6)	26.6(26.3,27.0)	6.3(3.6,10.8)	3.5(3.4,3.7)	15.0(7.4,22.6)	18.2(17.9,18.5)
Uttar Pradesh	Rampur	18.4(10.3,26.6)	18.3(17.9,18.7)	3.8(1.6,8.8)	2.8(2.7,3.0)	12.7(4.9,20.5)	15.3(15.0,15.6)
Uttar Pradesh	Saharanpur	7.6(2.2,12.9)	16.2(15.9,16.5)	1.6(0.5,4.8)	2.3(2.2,2.4)	7.6(2.2,12.9)	14.2(14.0,14.5)
Uttar Pradesh	Sant Kabir Nagar	21.4(11.2,31.7)	20.2(19.8,20.7)	1.9(0.7,4.9)	2.0(1.8,2.1)	11.3(4.6,17.9)	13.8(13.5,14.2)
Uttar Pradesh	Sant Ravidas Nagar (Bhadohi)	24.9(11.2,38.5)	26.4(25.9,26.9)	1.0(0.3,4.1)	4.6(4.3,4.8)	16.5(6.9,26.1)	17.1(16.7,17.5)
Uttar Pradesh	Shahjahanpur	9.0(3.6,14.5)	15.2(14.9,15.5)	2.0(0.7,5.1)	2.5(2.3,2.6)	5.7(2.1,9.2)	12.0(11.8,12.3)
Uttar Pradesh	Shrawasti	12.0(6.5,17.5)	19.9(19.3,20.4)	0.0(0.0,0.0)	1.2(1.1,1.4)	6.5(1.8,11.1)	10.6(10.2,11.1)
Uttar Pradesh	Siddharth nagar	11.8(5.5,18.0)	17.4(17.1,17.8)	1.2(0.4,3.7)	2.1(2.0,2.2)	7.4(3.9,10.9)	11.9(11.6,12.2)
Uttar Pradesh	Sitapur	21.1(14.1,28.2)	24.3(24.0,24.6)	2.0(0.7,5.2)	3.9(3.8,4.1)	18.6(12.5,24.8)	17.7(17.5,18.0)
Uttar Pradesh	Sonbhadra	19.9(10.6,29.2)	22.9(22.5,23.3)	0.0(0.0,0.0)	1.1(1.0,1.2)	16.1(7.5,24.8)	13.6(13.3,14.0)
Uttar Pradesh	Sultanpur	20.5(14.9,26.2)	19.1(18.8,19.3)	3.1(1.7,5.4)	2.4(2.3,2.5)	15.7(11.2,20.2)	12.9(12.7,13.1)
Uttar Pradesh	Unnao	26.5(20.0,33.0)	21.9(21.5,22.2)	8.9(5.2,15.0)	3.3(3.2,3.4)	21.0(14.8,27.2)	16.3(16.0,16.5)
Uttar Pradesh	Varanasi	21.9(11.0,32.9)	17.3(17.0,17.5)	1.2(0.3,4.7)	1.0(0.9,1.1)	20.5(10.6,30.5)	13.3(13.1,13.5)
Uttarakhand	Almora	34.0(21.7,46.2)	32.2(31.4,32.9)	7.0(3.5,13.4)	6.1(5.7,6.5)	28.5(22.1,34.8)	23.6(22.9,24.2)
Uttarakhand	Bageshwar	16.4(8.2,24.6)	26.3(25.2,27.4)	0.7(0.1,4.6)	5.2(4.6,5.7)	10.8(4.5,17.1)	17.3(16.3,18.2)
Uttarakhand	Chamoli	20.3(10.9,29.7)	24.5(23.6,25.3)	5.1(2.4,10.4)	5.0(4.6,5.5)	15.0(10.8,19.3)	17.4(16.6,18.2)
Uttarakhand	Champawat	29.1(16.7,41.5)	23.8(22.6,24.9)	5.9(3.2,10.7)	5.2(4.6,5.7)	14.9(7.7,22.2)	16.0(15.0,16.9)
Uttarakhand	Dehradun	26.9(15.5,38.4)	23.3(22.8,23.7)	9.6(5.6,16.0)	7.6(7.4,7.9)	19.3(14.5,24.0)	17.5(17.2,17.9)
Uttarakhand	Garhwal	19.0(6.7,31.2)	28.3(27.6,29.0)	2.2(0.5,8.2)	2.8(2.6,3.1)	10.6(4.8,16.3)	19.7(19.1,20.3)
Uttarakhand	Hardwar	30.4(19.6,41.2)	21.1(20.7,21.6)	7.3(3.3,15.2)	6.0(5.7,6.2)	28.9(18.8,39.0)	16.2(15.9,16.6)
Uttarakhand	Nainital	25.9(15.2,36.5)	29.0(28.3,29.6)	8.0(4.4,13.9)	6.8(6.4,7.1)	19.3(10.1,28.6)	20.7(20.1,21.2)
Uttarakhand	Pithoragarh	28.4(15.4,41.3)	29.0(28.2,29.8)	9.3(5.6,15.0)	6.0(5.5,6.4)	15.9(9.5,22.4)	20.3(19.5,21.0)
Uttarakhand	Rudraprayag	29.1(14.2,44.0)	29.4(28.3,30.6)	2.5(0.9,6.5)	8.7(8.0,9.5)	20.1(13.0,27.1)	19.7(18.7,20.8)
Uttarakhand	Tehri Garhwal	26.9(11.4,42.3)	25.1(24.4,25.8)	8.7(4.9,15.1)	5.6(5.2,6.0)	19.9(10.4,29.4)	18.5(17.8,19.1)
Uttarakhand	Udham Singh Nagar	25.7(15.0,36.5)	24.9(24.4,25.3)	4.5(2.1,9.4)	6.6(6.3,6.9)	25.7(15.0,36.5)	21.7(21.3,22.1)
Uttarakhand	Uttarkashi	30.6(16.4,44.9)	25.8(24.8,26.8)	5.9(3.1,11.0)	5.1(4.6,5.6)	18.1(8.4,27.8)	18.3(17.4,19.2)
West Bengal	Bankura	19.5(10.0,29.0)	26.1(25.8,26.4)	3.5(1.4,8.1)	4.0(3.9,4.1)	16.9(8.3,25.5)	23.0(22.7,23.2)
West Bengal	Barddhaman	19.4(13.7,25.1)	21.1(20.9,21.3)	5.0(3.2,7.8)	2.8(2.7,2.9)	18.2(12.9,23.5)	21.7(21.5,21.9)
West Bengal	Birbhum	24.2(18.7,29.7)	28.7(28.4,29.0)	6.0(3.3,10.9)	3.4(3.3,3.6)	20.8(15.2,26.4)	26.3(26.0,26.6)
West Bengal	Dakshin Dinajpur	29.3(21.4,37.2)	31.4(31.0,31.9)	5.3(2.8,10.0)	5.3(5.1,5.5)	26.7(19.8,33.6)	26.7(26.2,27.1)
West Bengal	Darjiling	33.8(19.2,48.4)	29.0(28.6,29.4)	7.3(4.2,12.2)	6.2(5.9,6.4)	33.8(19.2,48.4)	29.0(28.5,29.4)
West Bengal	Haora	22.1(14.3,29.9)	18.1(17.9,18.3)	5.9(3.2,10.8)	6.3(6.2,6.5)	22.1(14.3,29.9)	19.2(19.0,19.4)
West Bengal	Hugli	21.6(11.2,31.9)	18.6(18.4,18.8)	6.5(3.6,11.4)	4.1(4.0,4.2)	21.0(10.2,31.8)	19.1(18.9,19.3)
West Bengal	Jalpaiguri	22.1(13.2,30.9)	29.7(29.4,30.0)	3.8(1.8,7.8)	7.4(7.3,7.6)	22.1(13.2,30.9)	24.9(24.6,25.2)
West Bengal	Koch Bihar	20.1(10.9,29.3)	23.2(22.9,23.6)	0.6(0.1,4.0)	3.9(3.8,4.1)	19.1(9.7,28.4)	20.9(20.5,21.2)
West Bengal	Kolkata	17.5(9.2,25.9)	18.3(18.1,18.5)	7.0(3.4,13.9)	7.3(7.1,7.4)	15.1(8.9,21.3)	19.7(19.4,19.9)
West Bengal	Maldah	32.8(16.0,49.5)	24.8(24.5,25.1)	7.9(4.4,13.6)	4.7(4.6,4.9)	25.5(13.4,37.6)	21.3(21.1,21.6)
West Bengal	Murshidabad	13.8(2.7,25.0)	14.5(14.4,14.7)	4.3(2.2,8.5)	1.9(1.8,1.9)	13.2(1.8,24.6)	10.5(10.4,10.7)
West Bengal	Nadia	26.1(17.9,34.3)	20.0(19.7,20.2)	6.8(3.7,12.1)	5.9(5.8,6.0)	26.1(17.9,34.3)	19.7(19.5,19.9)
West Bengal	North Twenty Four Parganas	25.7(14.0,37.4)	23.1(22.9,23.3)	5.4(2.7,10.5)	6.8(6.7,6.9)	25.7(14.0,37.4)	23.6(23.4,23.7)
West Bengal	Paschim Medinipur	25.2(18.5,31.8)	22.1(21.9,22.4)	3.8(1.7,8.2)	3.9(3.8,4.0)	22.2(15.8,28.6)	20.1(19.9,20.3)
West Bengal	Purba Medinipur	12.0(3.7,20.2)	14.7(14.5,14.9)	2.2(0.7,6.5)	3.8(3.7,3.9)	9.9(1.7,18.1)	13.2(13.0,13.4)
West Bengal	Puruliya	38.5(30.9,46.2)	33.0(32.7,33.4)	4.0(1.8,8.6)	5.5(5.3,5.7)	31.3(21.9,40.6)	25.0(24.6,25.3)
West Bengal	South Twenty Four Parganas	11.8(5.4,18.3)	15.6(15.4,15.8)	1.0(0.3,4.0)	4.4(4.3,4.5)	11.3(4.7,17.9)	14.5(14.4,14.7)
West Bengal	Uttar Dinajpur	26.7(11.4,42.0)	30.0(29.6,30.4)	5.8(3.2,10.3)	4.4(4.3,4.6)	26.2(11.3,41.0)	28.8(28.5,29.2)

Sikkim had the highest percentage of women who were self-employed (16.7% (95% CI: 12.9–20.6)). The estimates of women who were self-employed in Sikkim ranged between 7.2% (6.3–8.2) in west district of Sikkim and 19.8% (18.8–20.8) in east district of Sikkim. The state with lowest percentage of women who were self-employed in the past 12 months was Karnataka (1.9%, 95% CI: 1.3–2.6), with prevalence ranging from 1.2% (95% CI: 1.1–1.3) in Uttar Kannada to 3.5% (95% CI: 3.3–3.7) in Kolar. The past year prevalence of self-employed women in Gujarat was 2.0% (95% CI: 1.4–2.5). The lowest and highest prevalence women who were self-employed in the past 12 months in Gujarat were estimated for Amreli (0.7%, 95% CI: 0.6–0.8) and Vadodara (3.4%, 95% CI: 3.3–3.5) districts, respectively.

Intra-state heterogeneity was also observed in the prevalence of women who earned cash in the past 12 months. In Telangana, the state with the highest prevalence of women who earned cash in the past 12 months was 45.1% (95% CI: 42.7–47.5), the prevalence of women who earned cash in the past 12 months ranged between 34.9% (34.6–35.2) in Rangareddy and 55.9% (55.5–56.3) in Nizamabad. Manipur was next to Telangana in terms of women who earned cash in the past 12 months; the district prevalence ranged between 34.8% (95% CI: 33.8–35.8) in Thoubal to 49.9% (48.2–51.6) in Chandel. The lowest percentage of women who earned cash in the past 12 months was in Bihar (12.6%, 95% CI: 11.5–13.8), with prevalence ranging from 8.4% (95% CI: 8.2–8.7) in Gopalganj to 20.7% (95% CI: 20.2–21.1) in Jamui.

#### Spatial patterns of women’s work in India

**Fig 2:**
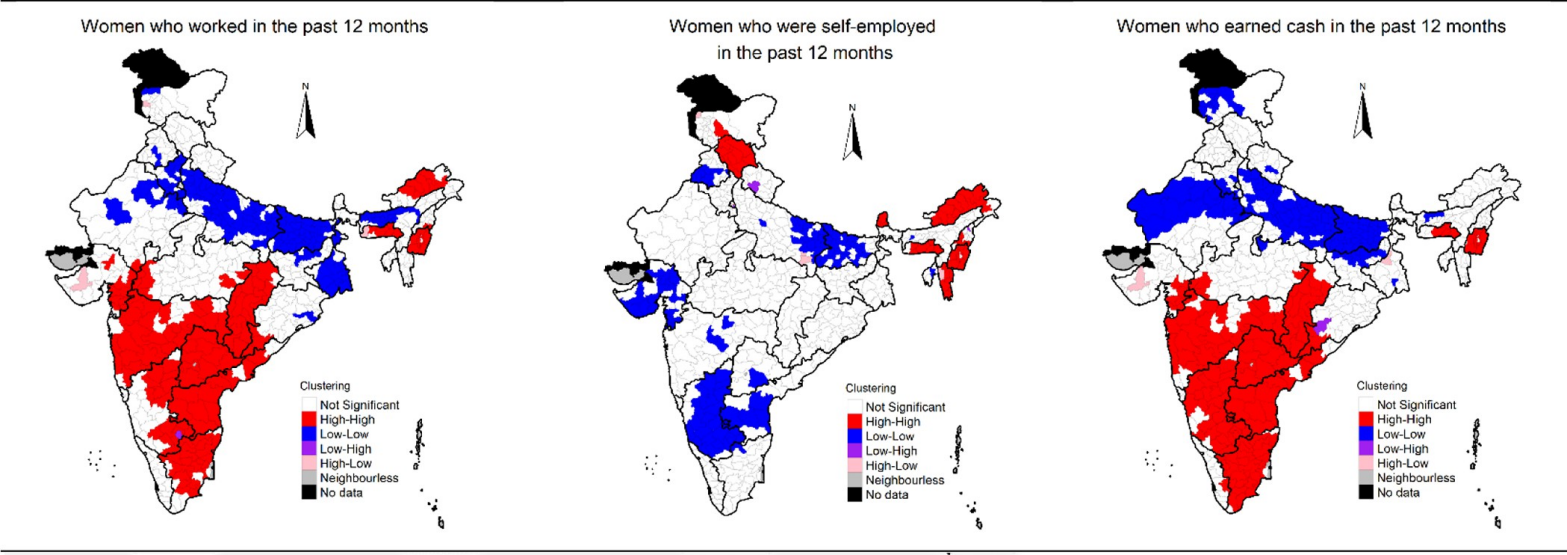
Univariate LISA maps depicting women who worked in the past 12 months, women who were self-employed in the past 12 months and women who earned cash in the past 12 months in districts of India, 2019–21. Note: The base map can be found at https://globalsolaratlas.info/download/india.

Univariate LISA maps shown in Fig 2 depict considerable spatial heterogeneity in the district-level estimates of women who worked in the past 12 months, women who worked as self-employed in the past 12 months, and women who earned cash in the past 12 months. High-high spatial clusters of women worked in the past 12 months were observed primarily in the districts of Andhra Pradesh, Chhattisgarh, Gujarat, Karnataka, Maharashtra, Telangana, Tamil Nadu and north-eastern states like- Arunachal Pradesh, Manipur, Meghalaya, Mizoram and Nagaland. A few districts of Madhya Pradesh, Jharkhand, Odisha and Rajasthan also formed high-high spatial clusters of women who worked in the past 12 months. In contrast, low-low clusters of districts of women who worked in the past 12 months were located primarily in Bihar, Haryana, Uttar Pradesh, West Bengal, and few districts of Assam, NCT of Delhi, Chandigarh, Jammu & Kashmir, Jharkhand, Odisha, Punjab and Rajasthan.

High-high spatial clusters of women who worked as self-employed in the past 12 months were found in the districts of Arunachal Pradesh, Himachal Pradesh, Manipur, Meghalaya, Mizoram, Nagaland and Sikkim. In contrast, low-low spatial clusters of women who worked as self-employed in the past 12 months were located in 105 districts primarily from Bihar, Gujarat, Karnataka, Punjab, Uttar Pradesh and few districts of Andhra Pradesh and Maharashtra.

High-high spatial clusters of women who earned cash in the past 12 months were found in the districts of Andhra Pradesh, Chhattisgarh, Karnataka, Maharashtra, Manipur, Puducherry, Tamil Nadu, Telangana, and a few districts of Gujarat, Kerala, Madhya Pradesh, Meghalaya and Odisha. In contrast, low-low spatial clusters of women who earned cash were located in 144 districts primarily from Bihar, Haryana, Jammu & Kashmir, Jharkhand, Rajasthan, Uttar Pradesh, and few districts of Assam, Himachal Pradesh and Uttarakhand.

**Fig 3:**
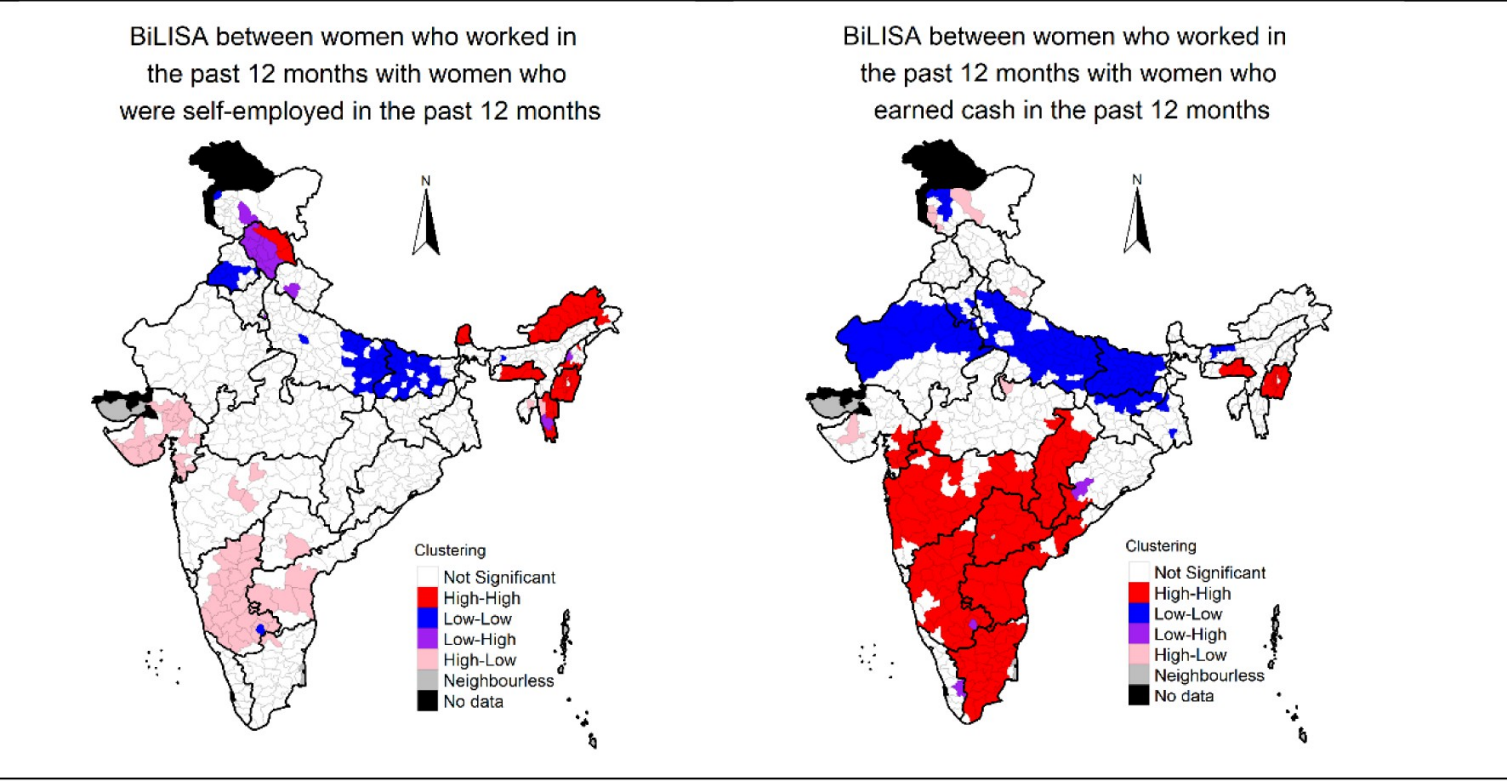
Bivariate LISA maps between women who worked in the past 12 months with women who were self-employed in the past 12 months; and women who worked in the past 12 months with women who earned cash in the past 12 months, India, 2019–21. Note: The base map can be found at https://globalsolaratlas.info/download/india.

Fig 3 shows bivariate association between district-level estimates of a) women who worked in the past 12 months and the women who worked as self-employed in the past 12 months and b) women who worked in the past 12 months and women who earned cash in the past 12 months. Districts with high estimated percentage of women who worked in the past 12 months and high percentage of women who worked as self-employed in the same reference period were found in Himachal Pradesh, and states from the north-eastern part of the country—Arunachal Pradesh, Manipur, Meghalaya, Mizoram, Nagaland and Sikkim. In contrast, districts with low estimated percentage of women who worked in the past 12 months and low percentage of women who worked as self-employed in the same reference period were found in Bihar, Punjab and Uttar Pradesh.

Districts with high estimated percentage of women who worked in the past 12 months and high percentage of women who earned cash in the same reference period were found in Madhya Pradesh, Chhattisgarh, Manipur, Gujarat, Maharashtra, Telangana, Andhra Pradesh, Karnataka and Tamil Nadu. On the contrary, districts with low estimated percentage of women who worked in the past 12 months and low percentage of women who earned cash in the same reference period were found in Bihar, Haryana, Jammu & Kashmir, Jharkhand, Rajasthan and Uttar Pradesh.

We also found cluster of districts in which percentage of women who worked in the past 12 months was high but the percentage of women who worked as self-employed was low. These clusters were found in Gujarat, Andhra Pradesh, Karnataka, three districts of Maharashtra (Parbhani, Hingoli and Jalna), Krishnagiri district of Tamil Nadu, and Nalgonda district of Telangana. In contrast, low percentages of women who worked in the past 12 months but high percentages of women who worked as self-employed in the past 12 months were found in the Hailakandi and Dima Hasao districts of Assam, Himachal Pradesh, Kishtwar district of Jammu & Kashmir, Lunglei district of Mizoram, Wokha district of Nagaland, Garhwal district of Uttarakhand, and central, south and north districts of NCT of Delhi. High percentage of women who worked in the past 12 months was associated with low percentage of women who earned cash in the past 12 months in Almora district of Uttarakhand, Jammu, Rajouri, Punch and Kargil districts of Jammu & Kashmir, and Rajkot and Kachchh districts of Gujarat. Low percentage of women who worked in the past 12 months was associated with high percentage of women who earned cash in the last 12 months in Bangalore district of Karnataka, Idukki, Ernakulum and Wayanad districts of Kerala, Sidhi district of Madhya Pradesh, Kalahandi district of Odisha, Karaikal district of Puducherry, and Chennai district of Tamil Nadu.

**Fig 4:**
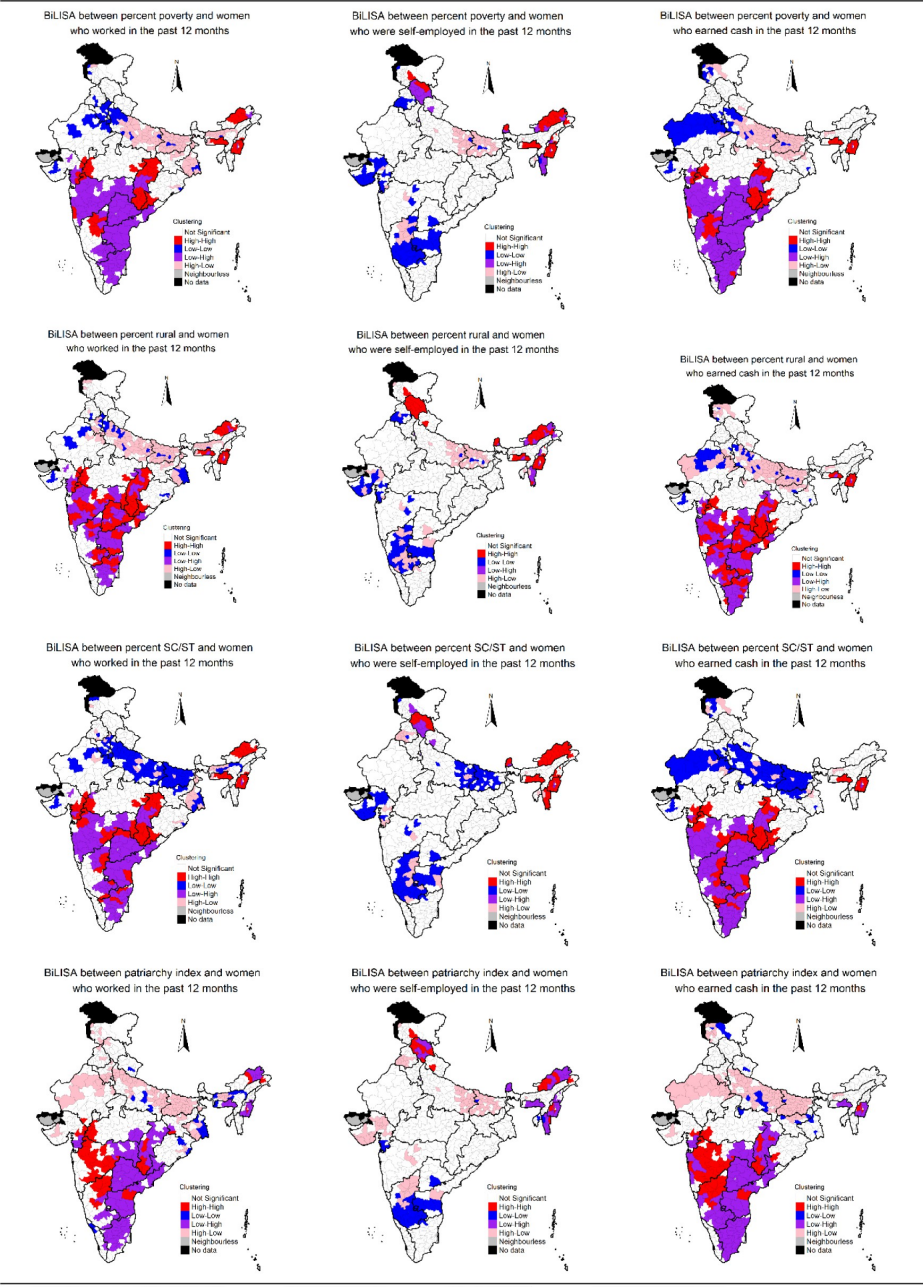
BiLISA maps to access association between demographic characteristics and women’s work in India, 2019–21. Note: The base map can be found at https://globalsolaratlas.info/download/india.

Fig 4 shows the bivariate LISA examining spatial association of the selected district-level characteristics with the three indicators of women’s work across the districts of India. Districts that had high poverty and high women’s work (measured in terms of both past year work and past year cash work) were found in Madhya Pradesh, Chhattisgarh, Gujarat, Karnataka, Maharashtra, Odisha, Meghalaya, Arunachal Pradesh, and Manipur. Districts that had low poverty and low women’s work were found in Jammu Kashmir, Punjab, Haryana, few districts of Rajasthan, West Bengal and Uttar Pradesh. We find distinct patterns when we examine spatial association between poverty and women who were self-employed in the past year. Districts from Arunachal Pradesh, Assam, Nagaland, Meghalaya, Manipur and Himachal Pradesh had high poverty and high percentages of women who were self-employed. In contrast, districts from Andhra Pradesh, Gujarat, Karnataka, and southern Punjab showed low levels of poverty and low proportions of women who worked as self-employed in past 12 months.

The findings further suggest that the districts marked by high levels of rural population and high women’s work and cash earning were concentrated in Madhya Pradesh, Chhattisgarh, Odisha, Arunachal Pradesh, Manipur, Telangana, Andhra Pradesh, Karnataka, Tamil Nadu, from the south region, Maharashtra and Gujarat from the west region. Bivariate LISA between percentage SC/ST and women’s work indicates that high-high clusters of women who worked in the past 12 months and percentage who worked for cash were predominantly found in Chhattisgarh and a few districts of Madhya Pradesh, Maharashtra, Gujarat, Meghalaya, Karnataka, Odisha, Arunachal Pradesh and Manipur. Low-low clusters of SC/ST and past year cash work were found in Uttar Pradesh, Bihar and selected districts of Rajasthan, West Bengal, Haryana, Jharkhand, and Jammu & Kashmir. High-high clusters of districts having higher percentages of SC/ST and self-employed predominantly belonged to North-eastern states and Himachal Pradesh. Low-low clusters of SC/ST and self-employment belonged to the districts from Bihar, eastern Uttar Pradesh, Gujarat, Karnataka and a few districts of Andhra Pradesh.

Interesting patterns emerge when we examine the bivariate LISA between IPI and women who worked in the past 12 months. Districts from Rajasthan, Haryana, Uttar Pradesh, and Bihar–states with high levels of patriarchy—were also the districts where fewer percentage of women worked in the past 12 months. In contrast, districts with low levels of patriarchy and high levels of women’s work were predominantly located in Tamil Nadu, Andhra Pradesh, Telangana, Maharashtra, Chhattisgarh, Meghalaya, Arunachal Pradesh and Manipur. A few districts of Madhya Pradesh bordering Maharashtra and a few districts of Odisha bordering Andhra Pradesh and Chhattisgarh formed a low patriarchy / high women’s work cluster.

When we look at women who worked for cash in the past 12 months, districts from Rajasthan, Haryana, Uttar Pradesh and Bihar formed high patriarchy and low women’s paid work clusters. In contrast, districts with low levels of patriarchy and high levels of women’s paid work were predominantly located in Tamil Nadu, Andhra Pradesh, Telangana, Karnataka, Maharashtra, Chhattisgarh, Meghalaya, Arunachal Pradesh and Manipur.

There were districts that, despite having high patriarchy, had high percentage of women who worked and earned cash in the past 12 months. Such districts were predominantly located in Maharashtra and Karnataka.

Results of OLS and SER are shown in Table 3. While mean age of women, percentage of women exposed to mass media, percentage of rural population and percentage of households who are SC/ST were positively associated with percentage of women who worked in the past 12 months in the OLS, mean number of children under the age of 5 years, percentage of females literate, percentage of Muslim households and percentage of poor households were negatively associated. India patriarchy index was negatively associated with women’s work in the past 12 months. In the SER model, percentage of women exposed to mass media, mean number of children under the age of 5 years and percentage poor households lost its statistical significance. Percentage of married women was positively associated with women’s work in the past 12 months in the SER. When it comes to women’s self-employment, percentage of women exposed to media, mean age of women, mean number of children under age of 5 years and percentage of SC/ST households were positively associated with self-employment. India patriarchy index and percentage of Muslim households were negatively associated with self-employment. In the SER, percentage of female headed households, percentage of married women and mean age of women and percentage of household who are SC/ST were positively associated with percentage of women who worked for cash. India patriarchy index, percentage of females literate and percentage of Muslim households were negatively associated with earning cash. The lag coefficient (λ) was statistically significant for all the three outcomes, indicating that the relationship between the three indicators of women’s work and selected district-level characteristics at the macro-level (districts) may be misleading if spatial clustering is ignored.

**Table 3:** OLS and spatial error model to assess the association between demographic characteristic and women’s work in India, 2019–21.

Variables	Women worked in the past 12 months				Women worked as self-employed				Women earned cash			
	Aspatial OLS		LM spatial error		Aspatial OLS		LM spatial error		Aspatial OLS		LM spatial error	
	Coefficients	Probability	Coefficients	Probability	Coefficients	Probability	Coefficients	Probability	Coefficients	Probability	Coefficients	Probability
Percentage of women exposed to media	0.13	0.00	0.04	0.11	0.04	0.00	0.03	0.00	0.09	0.00	0.01	0.60
Percentage of female headed household	0.10	0.55	0.17	0.15	0.05	0.22	-0.01	0.71	0.18	0.23	0.24	0.02
Percentage of married women	0.26	0.11	0.20	0.06	0.04	0.35	-0.02	0.53	0.26	0.09	0.25	0.01
Mean age of women	1.55	0.00	0.88	0.00	-0.06	0.54	0.21	0.01	1.81	0.00	0.74	0.00
Presence of child under age 5 years	-0.16	0.01	0.02	0.54	0.06	0.00	0.02	0.04	-0.16	0.00	-0.04	0.26
Percentage of female literacy	-0.29	0.00	-0.08	0.01	0.01	0.16	-0.01	0.32	-0.28	0.00	-0.08	0.02
Mean household size	-0.86	0.30	-0.43	0.47	-0.99	0.00	-0.10	0.58	-0.58	0.43	-0.30	0.58
Indian Patriarchy Index	-0.82	0.00	-0.34	0.01	0.05	0.18	-0.07	0.04	-1.10	0.00	-0.48	0.00
Percentage of Muslim households	-0.14	0.00	-0.09	0.00	-0.01	0.03	-0.02	0.01	-0.12	0.00	-0.08	0.00
Percentage of household who are SC/ST	0.11	0.00	0.08	0.00	0.05	0.00	0.03	0.00	0.03	0.12	0.04	0.00
Percentage of poor households	-0.06	0.02	0.01	0.60	-0.01	0.25	-0.01	0.42	-0.07	0.00	-0.01	0.54
Percentage of rural population	0.04	0.05	0.04	0.01	0.00	0.87	0.00	0.87	0.00	0.91	0.01	0.51
Constant	0.00	1.00	-7.11	0.03	-0.01	1.00	0.29	0.78	0.01	1.00	-2.83	0.36
No of observation	640.00				640.00				640.00			
log likelihood	-2304.95		-2071.98		-1406.67		-1260.67		-2235.58		-2018.15	
AIC	4635.91		4169.95		2839.33		2547.35		4497.16		4062.29	
R Square	0.43		0.78		0.34		0.64		0.46		0.78	
Lag Coefficient (lambda)			0.77	0.00			0.66	0.00			0.77	0.00
Breusch-Pagan test for heteroscedasticity	47.55	0.00	68.53	0.00	206.23	0.00	171.33	0.00	87.95	0.00	101.42	0.00
Likelihood Ratio Test			465.95	0.00			291.99	0.00			434.87	0.00

## Discussion

Our study is the first of its kind to provide district level estimates of women’s work in the past 12 months across 640 districts of India. We used area-level small-area estimation models, NFHS-5 survey data, and 2011 Indian Census data to generate these estimates. This research fills a substantial gap in the estimation of women’s work in India, given that estimates of women’s work-related indicators obtained directly from NFHS surveys are not reliable due to small sample sizes. Several diagnostic tests demonstrated the utility of SAE for estimating district-level estimates of women who worked in the past 12 months, women who worked as self-employed in the past 12 months and women who earned cash in the past 12 months in India. Reliable estimates of women’s work at the district-level can help policymakers and program managers to formulate and / or target relevant appropriate policies at more local levels. Unlike other surveys such as, National Sample Survey (NSS), PLFS and IHDS in India, which often provide data on women’s work at the national and state/province level, with no or little data at the local levels, NFHS-5 provides a unique opportunity to estimate women’s work at district-level using SAE. Moreover, every surveyed woman directly responded to the questions related to women’s work in NFHS-5. In contrast, any member of the household reports about the work done by other members of the household in all the labour force surveys conducted in India. Therefore, the likelihood of these surveys failing to capture women’s work is higher compared with NFHS-5. NFHS-5 also provided a description of the work that women usually do apart from their own housework. For example, NFHS-5 informed women that some women take up jobs for which they are paid in cash or kind and others sell things, have a small business or work on the family farm or in the family business. Then the NFHS-5 asked women whether they have done any of these things or any other work. Given the way the questions related to women’s work were designed in NFHS-5, NFHS-5 is less likely to miss unpaid work or self-employment. For the reasons mentioned above, our analyses demonstrate how a timely household survey, which is not a typical labour force survey, can be effectively used to provide reliable estimates of women’s work at more local levels.

In many low- and middle- income countries (LMICs), the primary source of data on economic activity at local levels is the population census, which is usually conducted at long intervals. Other specialized labour force surveys may exist, but may not provide estimates at more local levels. In absence of local level data, many LMICs rely on household survey data. This analysis offers an example of how commonly available household survey data (e.g., the NFHS or the Indian implementation of the Demographic and Health Surveys) can be combined with national population census data to estimate key metrics such as women work force participation, intimate partner violence, and the digital and financial inclusion of women that have been limited to more aggregate assessments.

Low levels of women’s work persist in India. Only 31% of women worked in the past 12 months, 25% of women earned cash in the past 12 months and only 4% of women were self-employed in the past 12 months. We observed significant geographical disparities at the district level. There are several districts within states where women’s work was very low while women’s work in the state as a whole is high. For instance, Madhya Pradesh, where 35% of women worked in the past year, had the highest variation across the districts; the estimated percentage of women who worked in the past 12 months ranged from 24% in Sidhi district to 61% in Alirajpur district. This sub-state heterogeneity underscores the inadequacy of state-level estimates in understanding and responding to the stagnation of women’s work in India.

A key contribution of the present study is to identify low-low and high-high clusters of women’s work. Identifying low-low clusters of districts is important for strategic targeted policies to increase women’s work in such clusters of districts. Identifying high-high clusters of districts offers valuable insights for best practices and interventions to improve women’s work in low-low clusters of districts. The study found that women’s work and cash earnings were lowest in the districts of central, eastern and northern parts of India. Districts from Uttar Pradesh (from central part) and Bihar (from the eastern part) have the lowest workforce participation among women. One of the possible reasons for low level of women’s work in Bihar and Uttar Pradesh may be natural disasters like flood. In these states, agriculture is the primary sector where women work [38]. Studies show that Bihar is the top-ranking state whose State Gross Domestic Product (SDGP) was affected by floods during 1983–2011. Likewise, Uttar Pradesh also ranked in top five states whose SGDP was affected by floods during the same period. In the context of frequent exposure to flood disasters, female farmers are more likely than male farmers to face unemployment because of dependency on agricultural work and lack of alternate employment opportunities due to resource and mobility restriction [39, 40]. Districts of Haryana and Punjab, from the northern region also show low levels of women’s work in the past year. Both states are known for their predominately agrarian economy known for intensive mechanisation; such economies require less labour [41]. In addition, these states are marked by high patriarchy [37]. In our spatial models, high patriarchy was associated with lower levels of women’s work, women’s self-employment, and women earning cash in the past 12 months.

In contrast, districts from southern-, western-, north-eastern- parts of India and Chhattisgarh depict the highest level of women’s work and cash earnings. This finding is in line with the other studies which also shows high workforce participation in these region [42]. In Andhra Pradesh and Telangana, women’s participation in agricultural activity is high compared to male worker [43]. Unlike in north India, characterized by wheat cultivation, large average landholding size, mechanized agriculture, and more limited involvement of women in agriculture, in southern India, agriculture is characterized by more manual labour associated with wet-rice cultivation than cultivation of wheat and other crops, leading to high involvement of women in agriculture [44]. Malhotra, Vanneman and Kishor (1995) used female share of labour force and area under rice cultivation (on the assumption that wet-rice cultivation involves substantial input from women than cultivation of wheat and other crops) as a proxy measure of women’s work [45]. Districts from Chhattisgarh, a less developed state of India, show high levels of women engaging in paid work, plausibly explained by the higher participation of women in MGNREGA scheme in this state [46]. Moreover, the high percentage of SC/ST population in Chhattisgarh could also explain high women’s work in Chhattisgarh. Chhattisgarh has the highest percentage of SC/ST population after the north-eastern states of Mizoram, Meghalaya, Nagaland and Arunachal Pradesh. Studies show that SC/ST women are more likely to work compared with women belonging to other social groups, such as other backward classes (OBC) and those who do not belong to SC/ST or OBC [36]. Women who do not belong to SC/ST are more likely to face restrictive norms than SC/ST women [47]. Our regression results also confirm positive associations between percentage of SC/ST households and all three indicators of women’s work. The Chhattisgarh government has also launched special programmes, such as *Naruva-Garuva-Ghuruva-Badi* to improve rural livelihood in the state. *Naruva-Garuva-Ghuruva-Badi* was launched in 2019 with a focus on water management, composting for soil health, animal husbandry and sustainable agriculture on backyard kitchen gardens [48]. Such programmes are likely to improve opportunity for women’s work in the state. Districts from the southern, western, north-eastern parts of India generally show lower levels of gender inequality compared with districts from the northern and central parts of India [37].

Further, our study shows high-high clusters of self-employed women in districts of Himachal Pradesh and north-eastern states, such as Arunachal Pradesh, Meghalaya, Manipur, Mizoram and Sikkim; all these states are mountainous. Our findings are consistent with the findings of prior studies [49, 50]. Studies have shown that women play a critical role in mountain societies as a significant proportion of workforce in agriculture [51] and managing and sustaining their natural resources and environment [52]. Samal (1993) argued that hill agriculture is totally dependent on women [53]. Recent research conducted in Uttarakhand and Manipur also argued that farming in the hilly areas has always been the domain of women [54, 55]. Women in hilly areas are also involved in a number of other activities, such as forest-based handicrafts, agro-forestry, livestock production, horticulture, and post-harvest activities [53–55]. Apart from being hilly, these states, except for Himachal Pradesh, are tribal dominated. Percent ST ranges from 33% in Sikkim to 94% in Mizoram [28]. Additionally, men from the hilly districts often migrate to the plain districts in search of better job opportunities and livelihood [56, 57].

Women from Kerala, despite high levels of schooling, show low levels of women’s work, including self-employment. High unemployment rates for women in Kerala may largely be due to constraints in skill utilisation, higher wage rates and massive inflow of remittances from migrants to the Gulf countries [58–60]. Past studies have also found U-shaped relationship between household income and female work participation in India [61–64]. When household income is low and agriculture activity dominates, there is high participation of women in the labour force. As household income rises women start withdrawing from the workforce. This withdrawal can be attributed to the ‘income effect’. But as the female education level increases further, the value of substitution effect (for highly educated women it would be costly to give up a paid job and take up an unpaid household work) also increases. As the substitution effect becomes greater than the initial income effect, women start moving back to the paid labour force. The evidence on this hypothesis is mixed and varies with geographical contexts [1, 65]. There is no clear evidence on the U-shaped relationship between economic development and women’s work in Kerala [58, 66].

We also found spatial outliers. For example, bivariate LISA between IPI and women’s work in past 12-months showed several outliers, i.e., districts with high patriarchy and high level of women’s work in the past 12-months. These outliers are primarily from Maharashtra and Karnataka. There were 13 districts from Maharashtra that had high women’s work despite having high levels of patriarchy. Our study is not powered enough to explain why a high proportion of women in these districts are working despite high levels of patriarchy. A study of such spatial outliers may shed light on what factors mitigate the effect of patriarchy when it comes to women’s work. Future studies may consider qualitative methods to research spatial outliers.

Female literacy was negatively associated with two of the three indicators of women’s work in the spatial regression models. A huge majority of women in India are employed in the agricultural sector [67]. With increase in literacy, women tend to withdraw from agriculture related activities and start looking for opportunity in the organized sectors, such as manufacturing, non-manufacturing (mainly construction) and service sectors (modern services) [67]. Chatterjee et al. (2018) also find out that women with increasing educational attainment are more likely to move from family farm or as wage labour to salaried positions [18]. The study further showed that if all or most available jobs were salaried, we could see a positive relationship between women’s education and employment. However, such job opportunities are limited in India [18, 68]. Micro-level studies for India have indicated that women withdraw from labour force with increase in women’s schooling and improvement in the status of the families [17, 18, 68].

Another finding that deserves mention is the negative association between the proportion of district residents who are Muslim and all three indicators of women’s work. Past studies have shown that Muslim women in India face educational, economic, social, and political marginalization, contributing to their low workforce participation in India [69–71]. Another study from India noted that Muslim women are often concentrated in areas of low economic activity due to which their participation in work is limited [72].

A key strength of our study is the use of the most recent population representative household survey, conducted in India in 2019–21, with district-level granularity. None of the other sources of data on women’s work in India offer such granularity. NFHS-5 provided us a unique opportunity to apply area-level SAE techniques to derive reliable estimates of the three indicators of women’s work for the 640 districts of India. Our analysis shows that direct survey estimates for many districts are unreliable due to large standard errors. These are the districts with very small sample sizes. Despite small sample sizes, the model-based estimates of women’s work are reasonable and representative for these districts. The direct and model-based 95% CIs are very close for the districts with reasonably large sample sizes. Another strength of our study is the use of spatial regression models to identify the factors associated with three indicators of women’s work at the district-level. Classical statistical models, such as OLS, often ignore spatial autocorrelation present in the data. Ignoring spatial autocorrelation may lead to underestimation of standard errors resulting in variables that should be statistically insignificant otherwise becoming significant. Spatial models also allowed us to identify clusters that require special attention. These spatial clusters may not always be spread within a state but may also be spread across the borders of neighbouring states. We have several examples where spatial clusters consisted of bordering districts of two or three neighbouring states. Such examples call for policies and programmes implemented jointly by group of neighbouring states. Our study also offers a convenient way to track progress in women’s work at regular intervals at lower administrative levels (such as a district), which cannot be easily done with census data that are collected at an interval of 10 years. As our work is based on NFHS-5 (Demographic and Health Survey (DHS) equivalent in India), our study can be easily replicated in other LMICs where DHS surveys are regularly conducted.

Our study has a few limitations. First, COVID-19 related lockdowns disrupted the collection of data in NFHS-5. Approximately 70% of the fieldwork in NFHS-5 was completed before the COVID-19 pandemic hit India and the rest was completed after the COVID-19 related lockdowns were lifted. This should not be a problem for our estimations as our three indicators of women’s work are based on a 12-months recall period, i.e. whether the woman has worked in the past 12 months. There are no reliable means of estimating the effects of the COVID-19 pandemic on the indicators of women’s work at the district level with NFHS-5 data. While we were able to estimate indicators of women’s work, we could not examine other aspects, such as women’s work-related decision-making, women’s entrepreneurship, etc., due to unavailability of such information in NFHS-5. Despite these limitations, our estimates of women’s work help us in understanding the geography of women’s work in India. Our estimates of women’s work may be used for future research including association of women’s work with intimate partner violence, women’s empowerment, women’s ownership of assets, demographic behaviour, etc.

### Conclusions

This study demonstrated the power of SAE technique to generate robust estimates of women’s work at the district level in India. Combining large scale survey data like NFHS-5 with 2011 population census allow us to produce reliable districts level estimates of women’s work and documented high intrastate variation in all three outcomes–percentage of women who worked in the past 12 months, women who earned cash in the past 12 months and women who were self-employed in the past 12 months. We also conducted spatial analyses and identified the cluster of districts with low and high women’s work. Our study indicates a clear north-south divide in indicators of women’s work in India. Our study demonstrates that women’s work varies substantially at the subnational level and having only state-level estimates may not be enough to address the issue of low women’s work in India. The insights from our current study may help in the formulation and implementation of policies that increase the returns on women’s work in India.

## Supporting information

S1 Text Definition of auxiliary variables. (DOCX)

S2 Text SAE methodology. (DOCX)

S1 Table Percentage variation explained by auxiliary variables for each outcome variable (Results based on OLS). (DOCX)

S1 Fig Model diagnostic plot showing the distribution of the district level residuals for women’s work in the past 12 months in India, 2019–21. (DOCX)

S2 Fig Plots comparing the ordinary least square regression line (dash line) and y = x (solid line), India, 2019–21. (DOCX)

S3 Fig District wise coefficient of variation for women’s work in the past 12 months in India, 2019–21. (DOCX)

S4 Fig District wise 95% CI for women’s work in the past 12 months in India, 2019–21. (DOCX)

## Abbreviation

CI: confidence intervals
CV: coefficient of variation
DHS: Demographic and Health Survey
GLMM: Generalized Linear Mixed Models
IHDS: India Human Development Survey
IPI: India Patriarchy Index
LISA: Local Indicator of Spatial Association
LMICs: low- and middle- income countries
MGNREGA: Mahatma Gandhi National Rural Employment Guarantee Act
MoSPI: Ministry of Statistics and Programme Implementation
NFHS: National Family Health Survey
NSO: National Statistical Office
NSS: National Sample Survey
NSSO: National Sample Survey Organization
OBC: other backward classes
OLS: ordinary least square
PLFS: Periodic Labour Force Survey
SAE: Small-area estimation
SC/ST: scheduled caste/ tribes
SDGP: State Gross Domestic Product
SER: Spatial Error Regression
SES: Socio-economic status
