# Supplementary material for: Mapping women’s work in India: An application of small area estimation
Source: PLoS One. 2025 Feb 19;20(2):e0317783. doi: 10.1371/journal.pone.0317783 (PMC11838883; doi:10.1371/journal.pone.0317783)
Supplement: S1 Text — (DOCX) [file pone.0317783.s001.docx]

**S1 Text**

**Definition of auxiliary variables**

Auxiliary variables were derived from the 2011 Indian Census (latest available Indian Census). The details of the auxiliary variables are given below:

1. Female literacy: Percentage of women age 15-49 who were literate
2. Gender gap relative to male literacy: The difference between percentage of male and female literates
3. Female headed household: Percentage of households headed by a female member
4. Female mean age at marriage
5. Birth of a male child in last one year: Percentage of male births in last one year
6. Mean household size in a district
7. Urban residence: Percentage of population residing in urban areas
8. Scheduled castes/tribes: Percentage of households belonging to the scheduled castes/tribes
9. Hindu: Percentage of households belonging to Hindu religion
10. Socio-economic status (SES)
11. Male migration in past 12 months: Percentage of male migrants in the last 12 months

**Estimation of district-level socio-economic status**

To construct district-specific SES, we used consumer durables (bicycle, motorcycle, car, telephone) and housing characteristics (type of wall, roof, floor, availability of electricity, sources of drinking water, toilet facility, availability of separate kitchen, clean fuel for cooking)^1^. Scores from these items were derived using principal components analysis at district-level, and further categorized into deciles, from lowest to highest; the lowest 10% of districts out of 640 districts are categorized as being in the lowest SES, second 10% of the districts being in the second lowest SES, and the highest 10% of districts are in the highest SES.

**References**

1. Filmer D, Pritchett LH. Estimating wealth effects without expenditure data—or tears: an application to educational enrollments in states of India. Demography. 2001; 38:115-32.
