# Supplementary material for: Mapping women’s work in India: An application of small area estimation
Source: PLoS One. 2025 Feb 19;20(2):e0317783. doi: 10.1371/journal.pone.0317783 (PMC11838883; doi:10.1371/journal.pone.0317783)
Supplement: S2 Text — (DOCX) [file pone.0317783.s002.docx]

**S2 Text**

**SAE Methodology**

We used generalized linear mixed model between district-level outcome variables and set of auxiliary variables. The model follows the binomial distribution with the logit link function. The present study also endorses that sampling weight should not be ignored while doing SAE analysis; otherwise, it may underestimate the prevalence of outcome variables.

***Theoretical illustration of SAE***

Present study illustrates the theoretical framework which is used to generate small area estimates of women’ s work and their method of precision across the district of India by following the approaches mentioned in previous studies^1^. Let $N_{d}$ and $n_{d}$ is the population and sample size in the district d (d=1, 2, 3 …. D), where D=640, is the number of the district in the population. The total number of units in the population is $N=\sum_{i=1}^{d} N_{i}$ with corresponding total sample size $n=\sum_{i=1}^{d} n_{i}$.

We used two subscript ‘s’ and ‘r’ to represent sample and non-sample population. It is y_sd_ and y_rd_, where y_sd_ is for sampled women in d^th^ district, and y_rd_ is for the non-sample women in d^th^ district. Here, the subscript d denotes the quantities belonging to district d. Then the outcome variable y_sd_ follows a binomial distribution with parameter n_d_ and π_d._ Where π_d_ is the probability of working/cash earning/self-employed in district d. y_sd_ and y_rd_ are assumed as the independent binomial variables with success probability π_d._

y_sd_ ~ Bin (n_d_, π_d_)

y_rd_ ~ Bin (N_d_-n_d_, π_d_)

Let x_d_ be the k vector of the covariates for the district d. The model linking this success probability with the covariates is the logistic linear mixed model of the form-

$logit\left( \pi d \right)=\left\{ \frac{\pi d}{1-\pi d} \right\}=\eta d=x_{d}^{'}\beta+u_{d}$ ………… (1)

d=1, 2, 3……., 640

Here, π_d_ =exp (ηd) {1+exp (ηd)}^-1^ and β is the k vector of unknown fixed-effects parameters. u_d_~N (0, ϕ) is the random effect that accounts for between district variability that explained by the covariates included in the model. Here we observe that model (1) shows direct estimates from the survey to the districts level covariates. This type of model is called as ‘area-level’ model in SAE terminology^2,3^ . But the Fay and Herriot method was based on the area level linear mixed model, and their approach applies to a continuous variable. In contrast, here the model (1) is the special case of a generalized linear mixed model (GLMM) with logit link function which is suitable for binary outcome variable^4^. Saei and Chambers have described this model in the context of SAE^5^. Therefore, the means of Y_sd ,_ Y_rd_ given u_d_ under model (1) are-

${E(Y\mathrm{sd}}/{u_{d})}=n_{d}\left[ exp\left( x_{d}^{'}\beta+u_{d} \right)\left\{ 1+exp\left( x_{d}^{'}\beta+u_{d} \right) \right\}^{-1} \right]$ …………... (2)

${E(Y\mathrm{rd}}/{u_{d})}=\left( N_{d}-n_{d} \right)\left[ exp\left( x_{d}^{'}\beta+u_{d} \right)\left\{ 1+exp\left( x_{d}^{'}\beta+u_{d} \right) \right\}^{-1} \right]$ ………… (3)

Let T_d_ is the total number of women in district d, then

$T_{d}=y_{sd}+y_{rd}$ (d=1, 2....640)

The first term $y_{sd}$ is the sample count (i.e., direct estimates from the survey) whereas the second term $y_{rd}$ is the nonsample count.Thus, an estimate $T_{d}^{^}$ of the total number of working women/cash earning/self-employed in district *d*, which is obtained by replacing $y_{rd}$ by its predicted value under model (1). That is-

$$T_{d}^{^}=y_{sd}+ y_{rd}^{^}=y_{sd}+\left( N_{d}-n_{d} \right)\left[ exp\left( x_{d}^{'}\beta+u_{d} \right)\left\{ 1+exp\left( x_{d}^{'}\beta+u_{d} \right) \right\}^{-1} \right]$$

…….……. (4)

The proportion ($p_{d}$) of working women/cash earning/self-employed in a district d is obtained as the total number of women within the district. Thus, an estimate of $p_{d}$ is-

$p_{d}^{^}=\frac{T_{d}^{^}}{N_{d}}$ …….……. (5)

Equation (1) is derived from unweighted sample which assumes that sampling design within areas is non-informative. Therefore, equation (4) ignores the complex survey design used in NFHS. If the sampling design is informative and survey weighted counts are available, there are two main difficulties. First, the values for the weighted sample counts will not necessarily be the integers 0, 1, 2, 3…. n_d_; rather they will take a value from a finite set of unequally-spaced numbers determined by the survey weights of the sample cases in area *d*. Second, the estimated sampling variance of the weighted sample counts, y_sd_ implied by the binomial distribution, i.e., V (y_sdw_) ≈ n_d_ P_iw_ (1-P_iw_) will be incorrect. Several studies suggested that one should use the ‘effective sample size’ (ESS) instead of actual sample size in model while analysing area level estimates as a binomial proportion^6^. We uses a subscript (v) in all the quantities linked with the ESS. We address the above two issues by defining an ESS n_d (e)_ and an “effective sample count” (ESC) y_sd (e)_ such that-

$y_{\mathrm{sd}\left( v \right)}=n_{d} \left( v \right)*P_{iw}$ …….……. (6)

This leads to $P_{iw}$ with its corresponding estimator of variance estimate v ($P_{iw}$). Then the model equation 1 applied ESC $y_{sd(e)}$in district d follows the binomial distribution-

$y_{sd(v)}=Bin (n_{d\left( v \right)}\pi_{d})$ …….……. (7)

**References**

1. Amoako Johnson F, Chandra H, Brown JJ, Padmadas SS. Estimating district-level births attended by skilled attendants in Ghana using demographic health survey and census data: An application of small area estimation technique. Journal of Official Statistics. 2010; 26(2): 341–359.
2. Fay RE, & Herriot RA. Estimates of income for small places: an application of James-Stein procedures to census data. Journal of the American Statistical Association.1979; 74(366a): 269-277
3. Rao JN. Small area estimation. John Wiley & Sons. 2005; 331.
4. Breslow NE, Clayton DG. Approximate inference in generalized linear mixed models. Journal of the American Statistical Association. 1993; 88(421): 9-25.
5. Saei A, Chambers R. Small area estimation under linear and generalized linear mixed models with time and area effects. Southampton Statistical Sciences Research Institute, University of Southampton. 2003; S3RI Methodology Working Papers, M03/15: 31
6. Korn EL, Graubard BI. Confidence intervals for proportions with small expected number of positive counts estimated from survey data. Survey Methodology. 1998; 24: 193-201.
