# Supplementary material for: Mapping women’s work in India: An application of small area estimation
Source: PLoS One. 2025 Feb 19;20(2):e0317783. doi: 10.1371/journal.pone.0317783 (PMC11838883; doi:10.1371/journal.pone.0317783)
Supplement: S1 Table — (DOCX) [file pone.0317783.s003.docx]

| **S1 Table.** Percentage variation explained by auxiliary variables for each outcome variable (Results based on OLS) | |
| --- | --- |
|  | **R-square (%)** |
| Percentage of women worked in the past 12 months | 74.1 |
| Percentage of women earned cash in the past 12 months | 75.9 |
| Percentage of women were self-employed in the past 12 months | 56.8 |
