# Supplementary material for: Mapping women’s work in India: An application of small area estimation
Source: PLoS One. 2025 Feb 19;20(2):e0317783. doi: 10.1371/journal.pone.0317783 (PMC11838883; doi:10.1371/journal.pone.0317783)
Supplement: S1 Fig — (DOCX) [file pone.0317783.s004.docx]

| **S1 Fig.** Model diagnostic plot showing the distribution of the district level residuals for women’s work in the past 12 months in India, 2019-21 | | |
| --- | --- | --- |
| 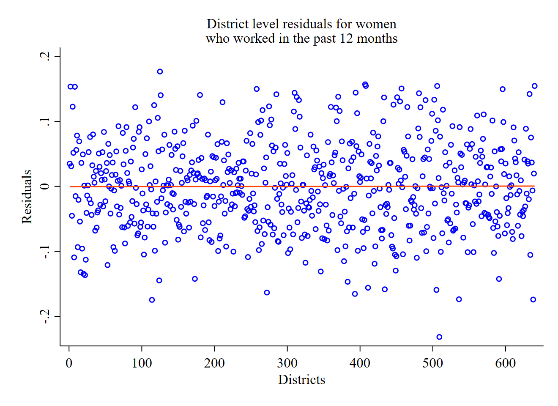 | 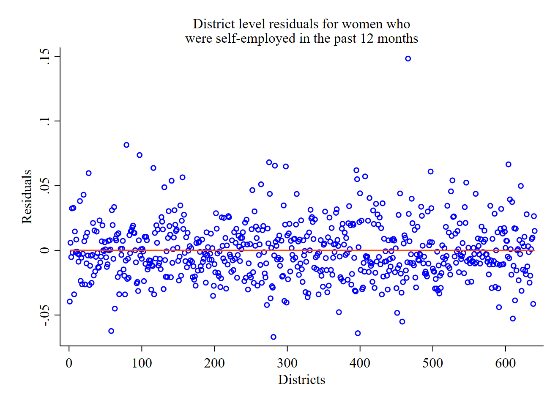 | 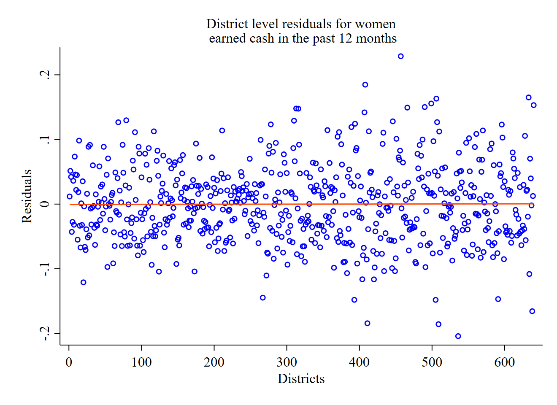 |
