# Supplementary material for: Mapping women’s work in India: An application of small area estimation
Source: PLoS One. 2025 Feb 19;20(2):e0317783. doi: 10.1371/journal.pone.0317783 (PMC11838883; doi:10.1371/journal.pone.0317783)
Supplement: S2 Fig — (DOCX) [file pone.0317783.s005.docx]

| **S2 Fig.** Plots comparing the ordinary least square regression line (dash line) and y=x (solid line), India, 2019-21 | | |
| --- | --- | --- |
| 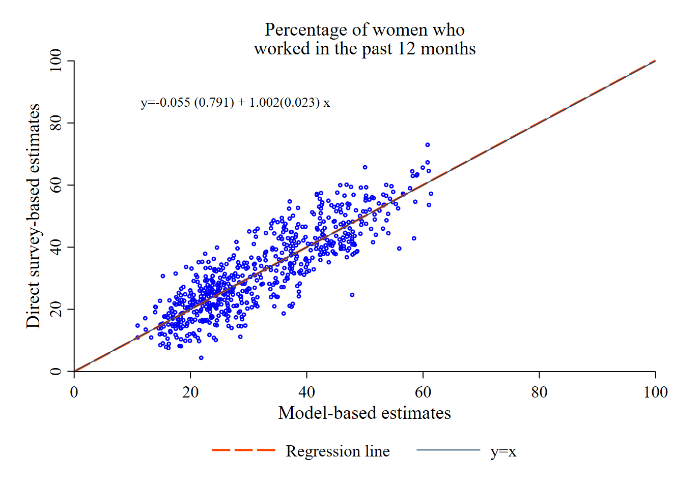 | 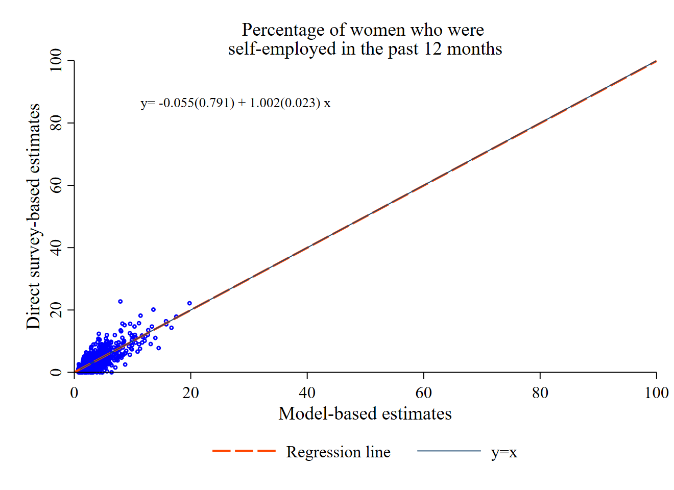 | 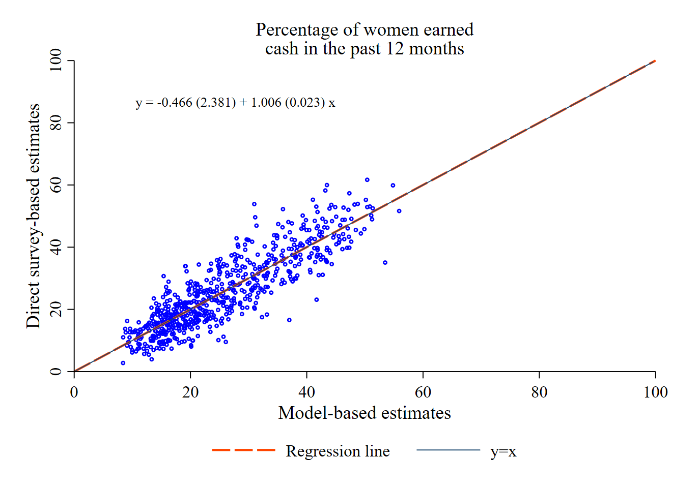 |
