# Supplementary figures and images for: Mapping women’s work in India: An application of small area estimation
Source: PLoS One. 2025 Feb 19;20(2):e0317783. doi: 10.1371/journal.pone.0317783 (PMC11838883; doi:10.1371/journal.pone.0317783)

| **S3 Fig.** District wise coefficient of variation for women’s work in the past 12 months in India, 2019-21 | | |
| --- | --- | --- |
| 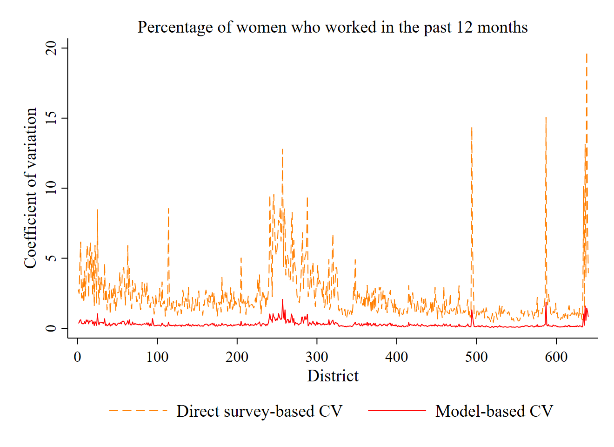 | 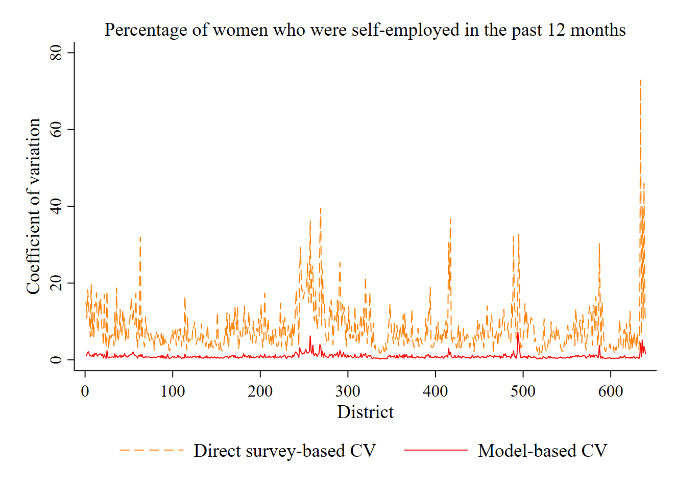 | 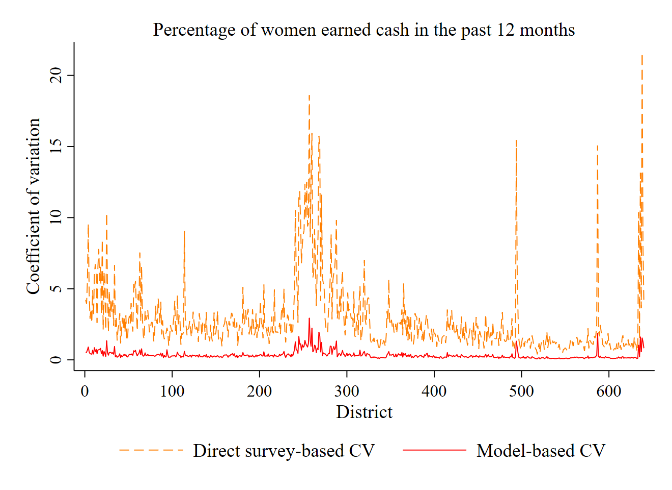 |

Supplement: S3 Fig — (DOCX) [file pone.0317783.s006.docx]

| **S4 Fig.** District wise 95% CI for women’s work in the past 12 months in India, 2019-21 | | |
| --- | --- | --- |
| 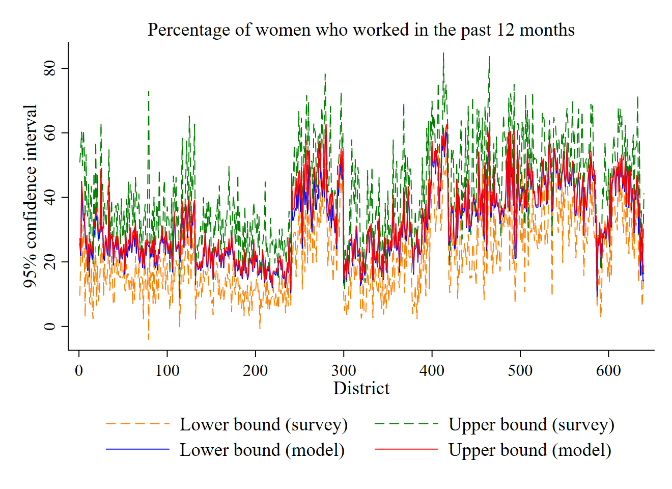 | 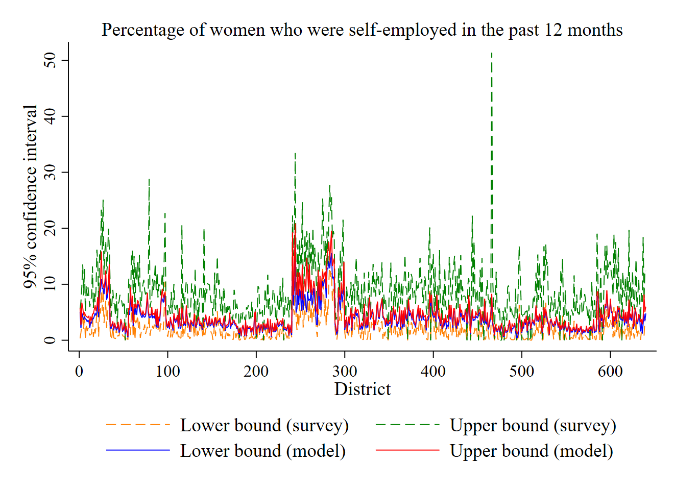 | 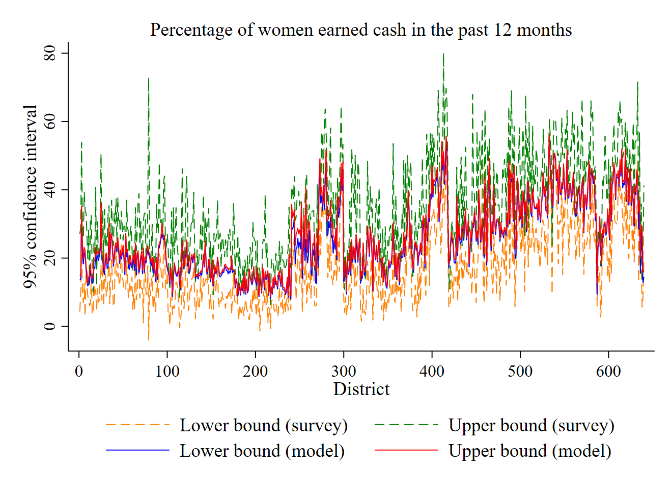 |

Supplement: S4 Fig — (DOCX) [file pone.0317783.s007.docx]
